# An Unequal Clustering and EPPR-Initialized Ant Colony Multihop Routing Protocol for Wireless Sensor Networks

**DOI:** 10.3390/s26216823

**Published:** 2026-10-28

**Authors:** Xingye Qu, Huiqing Cui

**Affiliations:** College of Engineering, Yanbian University, Yanji 133002, China; 2024050060@ybu.edu.cn

**Keywords:** wireless sensor networks, unequal clustering, link communication-energy-weighted personalized PageRank, ant colony optimization, dynamic reclustering

## Abstract

Wireless sensor network operation is constrained by uneven cluster loads, intercluster communication costs, and maintenance that does not reflect node condition. UEAD combines distance-adaptive unequal clustering, EPPR-initialized ant colony optimization (ACO), and health-driven maintenance. Smaller clusters near the base station limit member-data reception loads, while larger distant clusters reduce the number of remote source heads. The base-station-personalized EPPR prior uses radio-link communication costs to initialize ACO pheromones, and cluster health determines local head rotation or global reclustering. MATLAB R2025b simulations evaluate route quality, network lifetime, and control and computational overhead. At five iterations, the complete EPPR–ACO implementation reduced mean route energy from 2.348 to 1.940 mJ; ACO was slightly lower at 20–100 iterations. Equal-total-time tests, including EPPR preprocessing, showed an EPPR–ACO advantage at short budgets and similar energy at longer budgets. In the lifetime comparisons, UEAD delayed node loss and retained more residual energy than Standard LEACH and DUCISCA. Fixed-area, 50-round tests with 200–1000 nodes showed lower modeled control energy for UEAD under the stated accounting models, but substantially higher runtime at 1000 nodes.

## 1. Introduction

### 1.1. Research Background and Motivation

Wireless sensor networks (WSNs) connect physical environments to information systems by collecting and transmitting measurements from distributed sensor nodes. They support applications ranging from environmental monitoring and smart agriculture to industrial control, intelligent transportation, and military reconnaissance. Each node integrates sensing, computation, and wireless communication, often with limited battery capacity and processing resources. In remote or inaccessible deployments, replacing or recharging batteries may be impractical. Energy-efficient data collection is therefore essential to sustaining network operation.

Communication is a major source of energy consumption in WSNs, and its cost depends on transmission distance, traffic distribution, and network topology. These factors also determine how unevenly energy is depleted across nodes. Nodes that transmit over long distances or repeatedly relay traffic may exhaust their batteries while substantial energy remains elsewhere in the network. Such localized depletion can create energy holes and disrupt data collection. Extending network lifetime therefore requires both reducing communication costs and limiting persistent load imbalances.

Cluster-based routing addresses these concerns by organizing data collection around cluster heads. Low-Energy Adaptive Clustering Hierarchy (LEACH) uses probabilistic cluster-head election and periodic rotation to distribute aggregation and transmission duties [1,2]. Members send data to their cluster head, which aggregates the received data and transmits it directly to the base station. However, random election does not explicitly coordinate cluster sizes or head locations, distant heads still incur high transmission costs, and periodic reconstruction does not respond directly to energy degradation. These limitations motivate the unequal-clustering energy-aware dynamic routing protocol (UEAD) proposed here. UEAD uses distance-adaptive unequal clustering to regulate cluster sizes and source-head distribution. Energy-Aware Personalized PageRank (EPPR) initializes ant colony optimization (ACO) pheromones using first-order radio-link communication costs and base-station personalization. Here, “energy-aware” refers only to link communication cost; node residual energy does not directly enter EPPR transition weights. Cluster health and the proportion of unhealthy clusters determine whether maintenance takes the form of local cluster-head rotation or global reclustering. Together, these mechanisms coordinate cluster formation, intercluster routing, and topology maintenance as node energy declines.

### 1.2. The LEACH Protocol

LEACH organizes network operation into rounds, each comprising a setup phase and a steady-state phase [1,2]. Clusters are formed during setup, followed by scheduled data collection and transmission. The cluster-head role rotates among nodes to distribute the energy cost of receiving, aggregating, and forwarding data.

At the beginning of round r, each eligible node n draws a random number uniformly from $\left[0,\;1\right]$. The node becomes a cluster head if this number is below the threshold $T\left(n\right)$, defined as [1]:

(1) $$T\left(n\right)=\left\{\begin{array}{cc} \frac{p}{1-p\left(r\;\mathrm{mod}\left(\frac{1}{p}\right)\right)}, & n\in G\\ 0, & n\notin G \end{array}\right.$$

Here, p is the desired proportion of cluster heads, r is the current round index starting from zero, and G is the set of nodes that have not yet served as cluster heads in the current election epoch of 1/p rounds. The operator mod denotes the remainder operation. In this formulation, 1/p is assumed to be an integer. Once elected, a node is excluded from further cluster-head election for the remainder of the epoch. The threshold increases for the remaining eligible nodes as the epoch progresses, and eligibility is reset at the beginning of the next epoch.

The elected cluster heads broadcast advertisement (ADV) messages using nonpersistent carrier-sense multiple access (CSMA). Each message identifies the transmitting cluster head. Non-cluster-head nodes select a cluster based on the received signal strength of these advertisements and send a join-request message to the selected head.

Each cluster head then creates and distributes a time-division multiple access (TDMA) schedule for its members. During the steady-state phase, members transmit in their assigned slots and can switch off their radios outside those slots to conserve energy. The cluster head remains active to receive member data, aggregates the collected measurements, and transmits the resulting packet directly to the base station. Data collection continues over successive TDMA frames until the steady-state phase ends, after which a new round begins with cluster-head election and cluster formation.

### 1.3. Main Contributions

The main contributions of this study are summarized as follows:

(1) A distance-adaptive unequal-clustering method with deterministic greedy coverage forms smaller clusters near the base station and larger clusters farther away.

(2) A base-station-personalized EPPR prior based on radio-link communication costs initializes ACO pheromones for multihop routing under range, loop-avoidance, node-role, and exclusive-relay constraints.

(3) A health-driven maintenance mechanism selects local cluster-head rotation or global reclustering according to cluster condition, followed by the required route updates.

The remainder of this paper is organized as follows. Section 2 reviews related work and identifies the research gaps. Section 3 defines the network scenario, objectives, and constraints. Section 4 presents the UEAD protocol. Section 5 evaluates parameter sensitivity, clustering and routing behavior, EPPR guidance, module contributions, overall protocol performance, and base-station location sensitivity. Section 6 discusses limitations and future work, and Section 7 concludes the paper.

## 2. Related Work

### 2.1. Distributed Weighted Cluster-Head Election

LEACH uses a probabilistic threshold for autonomous cluster-head election and periodically rotates the role among nodes. It therefore avoids centralized cluster-head selection at the base station, keeping the election procedure relatively simple [1,2]. T-LEACH uses a residual-energy threshold to reduce unnecessary cluster-head reselection [3]. In basic LEACH, the election threshold depends on the desired cluster-head proportion and election history, without explicitly accounting for residual energy, position, or neighborhood conditions. Nodes with limited energy or unfavorable locations may consequently become cluster heads, and the elected heads may be unevenly distributed. Distributed weighted election extends this approach by using node and neighborhood information to adjust election probabilities, competition priorities, or replacement decisions.

Residual energy is a common basis for these adjustments. Distributed Energy-Efficient Clustering (DEEC) determines election probabilities from node energy relative to the estimated network average, adapting cluster-head selection to heterogeneous energy resources [4]. Other methods incorporate residual energy, initial energy, and the desired cluster-head count to favor candidates with greater energy reserves [5]. Statistical approaches use first- and second-order energy statistics to adjust election thresholds across different energy states [6]. Energy weighting can reduce the burden placed on depleted nodes, but energy reserves alone do not indicate whether a candidate offers short communication links or a suitable position within the cluster. Methods that rely on network-average energy estimates also depend on the accuracy of those estimates.

Distance and communication cost provide additional selection criteria. Multidimensional LEACH variants combine residual energy, base-station distance, and regional position in cluster-head election, while also considering distance and direction in intercluster forwarding [7]. Hybrid Energy-Efficient Distributed clustering (HEED) uses residual energy as its primary selection criterion and intracluster communication cost as a secondary criterion [8]. Other distributed scheduling methods combine received signal strength indicator (RSSI) measurements with residual energy to account for communication conditions [9]. These criteria address different costs: base-station distance is relevant to long-range transmission, whereas neighborhood communication cost favors compact clusters. When several indicators are combined through weighted scores, their normalization and relative weights can influence the resulting head distribution as deployment conditions change.

Further extensions incorporate neighborhood density and previous cluster-head assignments. EACR-LEACH combines residual energy, neighbor count, base-station distance, and historical cluster-head frequency to account for energy availability, local connectivity, transmission cost, and role distribution [10]. Unequal clustering methods also use energy and spatial information to adjust competition radii or election priorities [11,12]. Such criteria distinguish candidates more fully than an energy threshold alone. Nevertheless, local candidate scores do not necessarily determine how well the selected heads jointly cover the network. Depending on the deployment and competition rules, favorable candidates may be concentrated in dense regions, while sparse regions receive less effective coverage.

Distributed weighted election limits the need for centralized optimization, but its effectiveness depends on the information available locally and the rules used to combine it. Selecting suitable individual heads does not by itself ensure balanced cluster sizes or account for the forwarding load generated by the complete cluster configuration. Where periodic reclustering is retained, repeated advertisements and membership updates also remain part of the control overhead. These considerations motivate closer coordination between cluster formation, intercluster routing, and maintenance.

UEAD addresses this coordination by combining distance-adaptive competition radii with deterministic candidate ordering and greedy coverage. The construction considers the coverage of the selected heads collectively, linking cluster membership and source-head distribution to subsequent multihop routing through ordinary nodes. Health-driven maintenance then updates the structure as energy is depleted. The greedy procedure provides a practical way to construct the cluster set, although it does not guarantee a globally optimal partition.

### 2.2. Centralized Cluster-Head Optimization

Centralized clustering uses the base station’s computing resources to organize nodes from a network-wide view. Nodes report information such as position, residual energy, and connectivity, allowing the controller to coordinate cluster-head selection and membership assignment. Access to global information can reduce head concentration and improve the distribution of cluster sizes and intracluster distances. These benefits depend, however, on state collection and the dissemination of clustering decisions, both of which generate control traffic. Storage and computation requirements also grow with network size and the complexity of the optimization procedure.

LEACH-C provides an early example of this approach. During setup, nodes report their positions and residual energy to the base station, which constructs clusters and broadcasts the selected heads and memberships [2]. Centralized selection allows head locations and member assignments to be considered together, reducing the spatial variability associated with autonomous random election. Periodic reconstruction nevertheless requires repeated state reporting and coordination. The update interval therefore involves a tradeoff between control overhead and the relevance of the information used to form clusters.

Distance-based methods such as K-means treat clustering as a geometric partitioning problem. Cluster centers and memberships are updated iteratively to reduce within-cluster squared distances. LEACH variants combine K-means with stochastic optimization to form compact groups and select sensor nodes as cluster heads [13]. Grid-based K-means restricts the regions considered during clustering to reduce the computational burden [14], while threshold-driven sector K-means uses node counts to subdivide the deployment and considers residual energy and communication distance when selecting heads [15]. These methods provide explicit control over spatial partitioning, but their results depend on choices such as initialization and cluster count. Geometric centers must also be mapped to actual sensor nodes. Moreover, minimizing intracluster distance does not directly account for the cost of forwarding aggregated data to the base station.

Fuzzy and soft clustering allow more flexible membership assignments. Improved fuzzy C-means uses node density to estimate cluster count and combines membership information with node-to-head distance [16]. Soft K-means uses probabilistic membership, density-based initialization, and multiple cluster heads to improve load distribution [17]. Other centralized schemes use interval type-2 fuzzy evaluation to score candidates and construct unequal clusters [18]. A Canopy–K-means approach selects primary heads using residual energy and distance to the cluster center, while considering energy and base-station distance for backup heads [19]. These designs incorporate additional energy and spatial information, although their performance remains sensitive to the membership rules, selection criteria, and update procedures employed.

The main advantage of centralized clustering is its ability to coordinate decisions across the network. Its practical effectiveness depends on the accuracy and timeliness of reported state, the cost of global computation, and continued access to the controller. Spatially compact clusters also need not remain energy-balanced over time. If the clustering objective excludes intercluster forwarding, savings on member-to-head links may be offset by higher transmission or relay costs elsewhere. This makes the relationship between cluster formation, route selection, and subsequent maintenance important to lifetime-oriented design.

UEAD also adopts centralized planning. It constructs unequal clusters by combining base-station-distance-dependent competition radii with deterministic ordering and greedy coverage, selecting actual sensor nodes directly as cluster heads. Smaller competition radii near the base station constrain local coverage, while the resulting head distribution defines the sources for subsequent intercluster routing. Cluster health then determines when local rotation or global reconstruction is required. The approach links cluster formation to routing and energy-dependent maintenance, while retaining the information requirements of centralized control and providing no guarantee of global optimality.

### 2.3. Bio-Inspired and Hybrid Intelligent Optimization

Cluster-head selection and routing involve large discrete search spaces and competing objectives, including transmission cost, residual energy, hop count, and load distribution. Exhaustive search becomes increasingly costly as the number of candidates and constraints grows. Bio-inspired metaheuristics explore these spaces through stochastic search and iterative evaluation, seeking useful solutions within a limited computational budget [20,21,22]. Their effectiveness depends on how candidate solutions, objectives, and feasibility constraints are represented, as well as on parameter settings and termination criteria.

Particle swarm optimization (PSO) and genetic algorithms (GAs) are widely used to search for cluster-head configurations. PSO updates candidates using individual and swarm search histories, with fitness functions incorporating energy and spatial information. Energy-aware and discrete PSO approaches optimize head selection or cluster formation [20,23], while ESO-LEACH and Enhanced LEACH use PSO to improve on random election [24,25]. GAs explore head combinations through selection, crossover, and mutation. GABEEC rotates heads within static clusters [26], whereas EEWC considers cluster compactness, separation, and head count [21]. Simulated annealing (SA) permits probabilistic acceptance of worse candidates to broaden exploration and has been combined with GAs for clustering and multisink routing [27,28]. These methods accommodate several objectives, but their search behavior depends on the encoding and algorithm-specific parameters. Approaches that optimize continuous head positions also require a mapping to actual sensor nodes.

ACO constructs routes sequentially on a candidate graph, making its solution representation well suited to multihop routing. Next-hop selection combines pheromone values with local heuristic information, and pheromone updates reinforce favorable paths. Routing criteria such as distance, residual energy, hop count, and link quality can be incorporated into these components [22,29]. Applications include energy-aware data delivery, intercluster transmission in dense networks, and multihop routing over cluster-head graphs [30,31]. ACO has also been used to plan mobile-sink trajectories [32], although the constraints and objectives of mobile collection differ from those of static intercluster routing. For the present problem, its main advantages are explicit path construction and a pheromone representation that can incorporate information about the candidate network before iterative search begins.

The same mechanisms introduce several limitations. Uniform initial pheromones provide no prior preference among candidate edges, leaving early path selection to the heuristic and stochastic exploration. Strong reinforcement can subsequently reduce search diversity and favor premature convergence [22,29,30,31,33]. Performance also depends on evaporation, pheromone and heuristic weights, and the number of ants. Heuristics based only on distance may overlook relay energy, while pheromone values accumulated under an earlier network state may become less useful as nodes deplete or routes fail. Adaptive evaporation and multiobjective heuristics have therefore been investigated to improve search behavior [33]. Even when these modifications reduce route cost, their contribution to network lifetime depends on how routes are reused and updated.

Hybrid methods assign different parts of the optimization process to complementary algorithms. SA–GA combines annealing with genetic search [27,28], while PSO-ECSM couples cluster-head selection with mobile-sink management [34]. Such designs can coordinate decisions that would otherwise be optimized separately, but they also introduce additional parameters and potentially different objectives across stages. Their evaluation should distinguish route or clustering improvements from the costs of computation, state reporting, and repeated optimization. Module ablations are also needed to determine which component contributes to the observed protocol-level gains.

Graph-based node evaluation provides another source of information for route search. Studies of node importance in WSNs combine connectivity with energy fields or multiple node attributes to characterize contributions to network connectivity and forwarding [35,36]. This motivates examining whether information propagated through the candidate graph can improve ACO initialization beyond local link heuristics. The specific question considered here is whether communication-cost-based transitions and base-station personalization can provide a useful prior for constrained intercluster routing.

UEAD uses EPPR to compute this prior on a candidate graph containing the source cluster head, surviving ordinary nodes, and the base station. The EPPR transition weights reflect per-link communication energy computed from the first-order radio model, while base-station personalization incorporates the collection destination into the propagation process. The resulting node scores initialize ACO pheromones; ACO then constructs and refines routes under the specified feasibility constraints. EPPR is link communication-cost-aware rather than residual-node-energy-aware. Residual energy affects the active topology indirectly through node death, cluster-health evaluation, cluster-head rotation, and global reclustering, rather than through the EPPR transition weights.

### 2.4. Multihop and Mobile-Sink Extensions

In LEACH, cluster heads transmit aggregated data directly to the base station, avoiding the need to maintain intercluster routes [2]. This arrangement becomes costly when heads are far from the base station, particularly in large deployment areas or with a base station near the boundary. Two approaches address this distance-related cost: multihop forwarding to a static base station and data collection using mobile sinks. Multihop routing introduces relay selection and load-management requirements, whereas mobile collection adds movement, scheduling, and delay constraints.

Intercluster multihop routing replaces a long transmission with a sequence of shorter links. Relay selection may consider distance, residual energy, progress toward the base station, and link quality. Energy-aware schemes use forwarding backbones and residual-energy constraints to distribute traffic [37], while graph-based methods incorporate spatial and energy information into next-hop decisions [31]. Multihop transmission saves energy only when the reduction in amplifier consumption exceeds the additional transmission, reception, and control costs. Its benefit therefore depends on link lengths, radio parameters, traffic volume, and the number of relay stages.

These savings can be unevenly distributed. Nodes near a static base station may repeatedly forward traffic from distant sources, depleting their energy and creating hotspots or energy holes [11,38]. Distance- or hop-based objectives may concentrate traffic on a small set of relays, while unnecessarily long paths incur additional reception and retransmission costs. Node failures and changing energy states also require route updates, with associated discovery and signaling overhead. Route quality must therefore be assessed in terms of both communication cost and the load accumulated by relays over time.

Unequal clustering addresses part of this imbalance during cluster formation. In EEUC and related protocols where cluster heads also relay intercluster traffic, smaller clusters near the base station reduce member-data collection costs and preserve head energy for forwarding [11,38]. Other approaches coordinate competition ranges and routing in circular networks [39] or jointly adjust intracluster and intercluster decisions through software-defined fuzzy control [40]. However, cluster geometry alone does not determine how forwarding traffic is distributed. The resulting energy balance depends on the interaction between cluster formation, relay selection, and the timing of structural updates.

Mobile sinks alter the collection geometry by approaching cluster heads, rendezvous points, or selected regions. This can shorten wireless links and redistribute the forwarding burden associated with a fixed destination. Proposed methods plan collection paths from node locations [41], adapt trajectories to energy and distance conditions [42], or combine cluster-head selection with sink movement [34]. These benefits must be balanced against travel time and intermittent contact. Nodes may need to buffer data until the sink arrives, and reducing the number of collection stops can leave some sources dependent on multihop forwarding.

Metaheuristic trajectory planning typically optimizes collection locations, visiting order, and travel distance. Multiobjective PSO considers both travel distance and sensor energy [43], while enhanced ACO has been applied to mobile-sink path planning [32]. These problems differ from static next-hop selection because route feasibility also depends on movement and contact schedules. Practical performance consequently depends on constraints such as sink speed, dwell time, obstacles, and positioning accuracy, in addition to wireless communication costs.

UEAD focuses on multihop collection with a static base station. Its unequal clustering regulates member-data reception loads near the base station and the distribution of distant source heads. Cluster heads transmit only their own aggregated data, while surviving ordinary nodes provide intercluster relaying. EPPR initializes ACO pheromones from candidate link costs, connectivity, and base-station personalization. ACO then constructs feasible routes under node-role and exclusive-relay-use constraints. Cluster health governs local rotation or global reclustering, and affected routes are updated as the network state changes.

Recent studies have examined complementary aspects of efficiency in sensing and communication systems. EFSRNet combines multi-scale exposure normalization with dual-branch feature aggregation for overexposed face super-resolution [44]. In distributed learning, an adaptive federated-learning scheme integrates adaptive optimization and differential privacy while accounting for communication costs during collaborative training [45]. At the medium-access layer, an interference-aware, collision-free MAC protocol addresses channel contention in underwater wireless sensor networks [46]. These studies highlight the roles of data processing, communication-aware collaboration, and channel access in system-level efficiency. Complementing these efforts at the network layer, UEAD coordinates unequal clustering, EPPR-guided multihop routing, and energy-driven topology maintenance.

### 2.5. Summary and Research Gaps

Existing WSN protocols address energy consumption through cluster-head selection, spatial organization, route optimization, and collection scheduling. Fuzzy-based clustering has also been combined with machine-learning-based data reduction to improve WSN lifetime [47]. Unequal clustering, multihop routing, and mobile sinks provide different ways to reduce long-distance transmission costs and concentrated forwarding loads [11,31,34,39]. Their lifetime benefits depend on how these decisions interact as node energy declines. The preceding review motivates three design questions concerning clustering–routing coordination, route-search initialization, and the scope of maintenance.

First, improvements in cluster formation do not necessarily translate into lower network-wide communication costs. Several methods emphasize candidate scoring or spatial partitioning [10,14,15,16,17,18,19,47], while others primarily optimize forwarding paths [22,29,30,31,33,37]. Compact clusters can reduce member-to-head transmission costs yet leave distant heads dependent on costly routes. Conversely, route optimization has limited scope to correct an unfavorable distribution of source heads and members. Cluster construction and route planning should therefore be assessed together, including their effects on both intracluster traffic and intercluster relay loads.

Second, ACO initialization offers an opportunity to incorporate information beyond local link heuristics. Uniform initial pheromones do not distinguish candidate edges by their positions in the network, although subsequent decisions can use distance, energy, or link-quality information [22,29,30,31,33]. This motivates evaluating whether graph propagation, informed by communication costs and the base-station location, can improve route quality under a limited ACO iteration budget. Such a prior can guide initial exploration while leaving route construction and refinement to ACO. Its value must be established through comparison with uniform initialization under otherwise identical conditions.

Third, maintenance must respond to energy degradation at an appropriate spatial scale. Periodic reconstruction and energy-triggered head replacement involve different tradeoffs between signaling overhead and response time [2,3]. A head’s residual energy alone does not capture energy conditions across its cluster. Local replacement can address isolated degradation while preserving memberships, but it may be insufficient when many clusters deteriorate. Global reclustering permits broader reorganization at the cost of more extensive coordination. Cluster-level state can provide a basis for deciding when each action is warranted.

UEAD combines these considerations within a centralized protocol for static WSNs. Distance-adaptive competition radii and greedy coverage regulate cluster memberships and the spatial distribution of source heads. EPPR computes node scores from radio link costs and base-station personalization on a candidate graph containing the source head, surviving ordinary nodes, and the base station. These scores initialize ACO pheromones for constrained multihop routing through ordinary relays. Residual energy informs cluster health and subsequent topology maintenance rather than directly weighting EPPR transitions. When the proportion of unhealthy clusters reaches the global threshold, UEAD performs global reclustering and route planning; otherwise, unhealthy clusters undergo local head rotation and affected-route updates. This design links initial cluster formation and route search to maintenance throughout network operation.

## 3. Materials and Methods: System Model and Problem Formulation

### 3.1. Network Scenario and Assumptions

We consider a two-dimensional static WSN comprising a base station and energy-constrained sensor nodes. The monitoring region is:

(2) $$\Omega =\left[0,X\right]\times \left[0,Y\right],$$

The sensor-node set is:

(3) $$V=\left\{v_{1},v_{2},\ldots ,v_{N}\right\},$$

where N is the number of sensor nodes and $v_{\mathrm{BS}}$ denotes the base station. The positions of node $v_{i}$ and the base station are $p_{i}=\left(x_{i},y_{i}\right)$ and $p_{\mathrm{BS}}=\left(x_{\mathrm{BS}},y_{\mathrm{BS}}\right)$, respectively.

The Euclidean distance between sensor nodes $v_{i}$ and $v_{j}$ and the distance from node $v_{i}$ to the base station are:

(4) $$d_{ij}=\sqrt{{\left(x_{i}-x_{j}\right)}^{2}+{\left(y_{i}-y_{j}\right)}^{2}},\;d_{i,\mathrm{BS}}=\sqrt{{\left(x_{i}-x_{\mathrm{BS}}\right)}^{2}+{\left(y_{i}-y_{\mathrm{BS}}\right)}^{2}}.$$

Operation is divided into consecutive transmission rounds. Let $E_{i}\left(r\right)$ denote the residual energy of node $v_{i}$ at the beginning of round r. The surviving-node set in that round is:

(5) $$V_{a}\left(r\right)=\left\{v_{i}\in V|E_{i}\left(r\right)>0\right\}.$$

A node whose residual energy reaches zero is marked dead at the end of the round and excluded from subsequent head selection, membership assignment, and route planning.

The following assumptions describe the proposed UEAD protocol.

1. Nodes remain stationary after deployment, have unique identifiers, and can obtain their own positions and residual energy.
2. Ordinary sensor nodes have identical initial capabilities and communication functions but limited batteries. The base station has a stable power supply and greater computation and storage capacity. Its energy consumption is excluded from sensor-network energy accounting.
3. Nodes establish wireless links to reachable nodes within the permitted range and adjust transmit power according to distance. Links remain stable within a transmission round.
4. The network uses clustered data collection. Ordinary nodes send sensed data to their heads, which aggregate the data and forward them to the base station along multihop paths formed by ordinary nodes. Each surviving node belongs to exactly one cluster per round.

Topology changes as nodes consume energy and die. The protocol must determine clusters and head-to-base-station paths from spatial distribution and energy state and adjust failed or degraded local topology during operation.

### 3.2. Optimization Objectives and Constraints

Let $V_{a}^{r}$ be the surviving-node set in round r, $C_{r}$ the cluster partition, and $P_{r}$ the set of head-to-base-station paths. Maintenance action $u_{r}$ takes values 0, 1, and 2 for retaining the structure, local head rotation, and global reclustering, respectively. These variables are determined jointly as energy evolves. Stable operation, midlife service, tail lifetime, and control cost define the multiobjective evaluation vector:

(6) $$maxF=\left(R_{FND},R_{HND},R_{LND},-E_{ctrl}\right)$$

Here, $R_{FND}$, $R_{HND}$, and $R_{LND}$ denote the rounds of first node death (FND), half node death (HND), and last node death (LND), respectively; $E_{ctrl}$ is cumulative control energy. This expression describes interrelated, potentially competing objectives without assigning FND an absolute, noncompensable priority. Parameter selection first considers stability, then HND, LND, and control energy. Protocol comparisons report all metrics, without treating any single result as global optimality.

Node energy obeys round-by-round conservation. Let $E_{i}^{\mathrm{data}}\left(r\right)$ and $E_{i}^{\mathrm{ctrl}}\left(r\right)$ be the energy deducted for data- and control-packet transmission and reception by node i in round r, respectively. Then:

(7) $$E_{i}\left(r+1\right)=\max\left\{0,\;E_{i}\left(r\right)-E_{i}^{\mathrm{data}}\left(r\right)-E_{i}^{\mathrm{ctrl}}\left(r\right)\right\},\;i\in V_{a}\left(r\right)$$

Base-station computation and transmission are excluded from this sensor-node energy ledger. If residual energy cannot cover the current packet, the node is allowed to complete it, its energy is clipped to zero at the round end, and it leaves the network. The resulting deficit is recorded separately and is not transferred to other nodes.

Each surviving sensor must belong to exactly one cluster per round. Let $x_{ic}\left(r\right)$ indicate whether node i belongs to cluster c. Clustering feasibility requires:

(8) $$\underset{c\in C\left(r\right)}{\sum }x_{ic}\left(r\right)=1,\;x_{ic}\left(r\right)\in \left\{0,1\right\},\;i\in V_{a}\left(r\right)$$

For any source head s, path $p_{s}$ must comprise physically reachable links. Let $R_{\mathrm{comm}}$ be the effective physical communication radius and $C_{uv}\left(r\right)$ the sensor communication cost of link $\left(u,v\right)$ in round r. Each hop satisfies:

(9) $$d_{uv}\leq R_{\mathrm{comm}},\;\left(u,v\right)\in p_{s},\;p_{s}\in P\left(r\right)$$

Among reachable path sets, intercluster routing locally minimizes the link communication costs of all source heads:

(10) $$\underset{P\left(r\right)}{\min}J_{\mathrm{route}}\left(r\right)=\underset{p_{s}\in P\left(r\right)}{\sum }\underset{\left(u,v\right)\in p_{s}}{\sum }C_{uv}\left(r\right)$$

Let $H\left(r\right)$ denote the current head set and $I\left(p_{s}\right)$ the intermediate-node set of path $p_{s}$. UEAD prohibits other heads from relaying and requires loop-free paths terminating at the base station. Thus:

(11) $$I\left(p_{s}\right)\subseteq V_{a}\left(r\right),\;I\left(p_{s}\right)\cap H\left(r\right)=⌀$$

To prevent load concentration from shared ordinary relays, exclusive relay use requires:

(12) $$\underset{p_{s}\in P\left(r\right)}{\sum }1\left[v\in I\left(p_{s}\right)\right]\leq 1,\;v\in V_{a}\left(r\right),\;v\notin H\left(r\right)$$

Maintenance depends on the unhealthy-cluster set $L\left(r\right)=\{c\in C\left(r\right)|H_{c}\left(r\right)<T_{H}\}$ and its proportion $P_{\mathrm{low}}\left(r\right)$. To make local rotation and global reclustering mutually exclusive at a check and avoid indefinitely postponing global maintenance, the action is:

(13) $$u\left(r\right)=\left\{\begin{array}{cc} 2, & P_{\mathrm{low}}\left(r\right)\geq T_{P},\\ 1, & P_{\mathrm{low}}\left(r\right)<T_{P}\;\wedge \;L\left(r\right)\neq ⌀,\\ 0, & L\left(r\right)=⌀. \end{array}\right.$$

Here, $T_{P}$ is the unhealthy-cluster proportion threshold. Global reclustering has priority when this threshold is reached. Local rotation occurs only below the global threshold when unhealthy clusters exist; otherwise, the structure is retained. Failure handling is distinct from this health decision: a dead CH is replaced before the next round’s member-to-head data collection, whereas an ordinary relay failure triggers repair only of paths using that relay. Neither response waits for the next scheduled health check.

## 4. UEAD Protocol Design

Cluster-head selection, intercluster routing, and maintenance affect different components of network energy consumption, but their effects accumulate over successive transmission rounds. In large WSNs with a base station at the boundary or outside the deployment area, differences in transmission distance and directional traffic convergence can place sustained demands on particular nodes. Improving cluster geometry or individual routes alone may therefore leave persistent load imbalances as the network ages.

UEAD coordinates these decisions through centralized planning at the base station. Distance-adaptive unequal clustering determines cluster memberships and the distribution of source heads, while EPPR-initialized ACO constructs multihop routes through ordinary relay nodes. During operation, cluster health and the proportion of unhealthy clusters govern local cluster-head rotation or global reclustering, with corresponding route updates. This feedback links cluster formation and routing to changes in node energy, allowing the scope of maintenance to follow the extent of degradation.

### 4.1. Design Objectives and Overall Framework

#### 4.1.1. Design Objectives

Energy consumption in a clustered WSN depends on member-to-head transmission, data reception and aggregation at cluster heads, forwarding by ordinary relays, and control exchanges. These costs vary across nodes and accumulate over successive rounds. A reduction in one component may therefore be offset by increased demand elsewhere. UEAD addresses this interaction through spatially unequal clustering, informed route initialization, and health-driven maintenance.

Distance-adaptive competition radii regulate cluster coverage according to base-station distance. Smaller radii near the base station limit the coverage of individual heads and are intended to reduce their member-data reception loads. Larger radii farther away allow each head to cover a wider area, reducing the need for multiple distant source heads. The resulting memberships depend on node placement and greedy coverage. Unequal clustering thus shapes intracluster loads and the distribution of source traffic, while relay selection is handled separately during routing.

Route quality under a limited iteration budget also depends on how ACO begins its search. Uniform initial pheromones provide no differentiated prior, although local heuristics can still distinguish links by cost or other attributes. UEAD supplements these heuristics with EPPR scores derived from first-order radio link costs and base-station personalization. The steady-state scores are normalized and mapped to initial pheromone values, incorporating information from the candidate graph before route exploration begins. ACO then constructs paths and updates pheromones. This initialization is intended to improve early search quality when routes must be replanned after cluster-head rotation, global reclustering, or node failure.

During operation, differences in collection and forwarding loads cause clusters to deplete energy at different rates. UEAD evaluates cluster health using mean residual energy and energy dispersion. At each health check, an unhealthy-cluster proportion at or above the global threshold triggers global reclustering. Below that threshold, local cluster-head rotation is applied to unhealthy clusters; if no unhealthy clusters are present, the cluster structure is retained. Routes invalidated by node death are repaired independently in the next round. These rules adapt maintenance scope to the observed degradation while keeping local rotation and global reclustering mutually exclusive at each check.

UEAD has the following objectives:

1. Adapt cluster coverage to node-to-base-station distance, reducing member-data reception loads at nearby heads and limiting the number of distant source flows.
2. Incorporate topology and communication-cost information into ACO initialization through EPPR to improve route quality under a limited iteration budget.
3. Construct cluster-head-to-base-station routes that satisfy communication-range constraints, use only ordinary nodes as relays, prohibit relay sharing across active routes, and contain no loops.
4. Apply mutually exclusive rules for local cluster-head rotation, global reclustering, and structure retention, with priority given to global reclustering when its threshold is reached, while repairing failed routes on demand.
5. Perform clustering, route planning, and maintenance decisions at the base station while accounting for modeled sensor-node energy costs associated with state reporting, notification reception, and data communication.

#### 4.1.2. Overall UEAD Architecture

**Figure 1:**
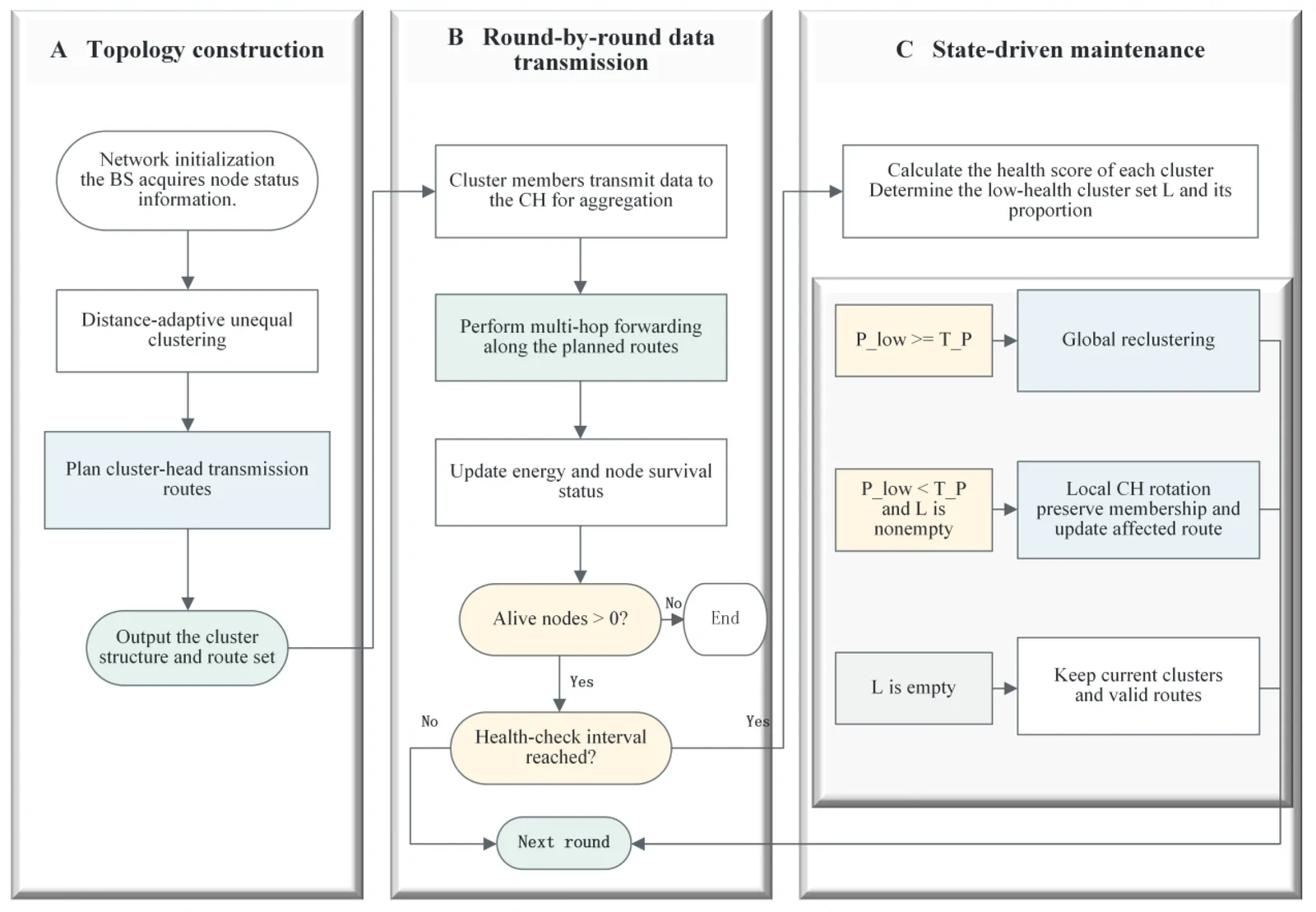
UEAD operation framework. Arrows indicate the direction of processing and control flow. Box colors visually distinguish workflow elements and do not encode numerical values.

UEAD comprises unequal clustering, EPPR–ACO route planning, and dual-threshold maintenance. During initialization, the base station obtains node identifiers, positions, initial energy, and connectivity information and establishes the surviving-node set. It then computes distance-dependent competition radii and forms clusters through deterministic greedy coverage. Candidates are considered in descending order of competition radius. Each selected candidate becomes a cluster head, and unassigned nodes within its coverage join the cluster. The procedure continues until every surviving node has been assigned. Figure 1 summarizes the UEAD operation framework.

The base station next plans collection routes for the selected heads. For each source head, it constructs a candidate graph containing that head, all surviving ordinary nodes, and the base station. Links are defined by the communication-range constraint and assigned radio energy costs. EPPR uses base-station personalization to compute steady-state node scores, which initialize ACO pheromones. ACO then constructs routes subject to communication-range, node-role, exclusive-relay-use, and loop-free constraints. A head may transmit directly when the base station is within range; otherwise, its route uses ordinary relay nodes.

Once clusters and routes are established, the network enters steady-state transmission. In each round, ordinary members send their measurements to their assigned heads. Each head receives and aggregates the data, then sends its aggregated packet along the planned route, with intermediate ordinary nodes performing reception and retransmission. Sensor nodes execute the data and control exchanges, while the base station performs EPPR computation, ACO search, health evaluation, and maintenance planning.

At scheduled health checks, the base station evaluates cluster health and the proportion of unhealthy clusters. If that proportion reaches the global threshold, all surviving nodes are reclustered and the routes are replanned. Otherwise, unhealthy clusters undergo local head rotation, retaining their memberships and updating only the affected routes. If no clusters are unhealthy, the current cluster structure is retained.

Failure handling is event-driven and separate from the 50-round health checks. When a CH dies at the end of a round, the base station immediately invokes the local replacement procedure in Section 4.4.2 before the next round’s member-to-head data collection. The surviving members therefore send their data to a live replacement CH without waiting for the next health check. The former CH’s route and any other paths that use the replacement as an ordinary relay are invalidated; the relay reservations associated with these paths are released, unaffected paths are retained, and the new CH’s route and all affected paths are replanned before the next round. By contrast, the death of an ordinary relay leaves cluster-head and membership assignments unchanged: only paths containing that relay are invalidated and repaired. CH replacement restores intra-cluster data collection, whereas the no-route rule governs subsequent forwarding from a live CH to the BS. Thus, if a valid route cannot be restored, the replacement CH can still collect and aggregate member data, but its aggregate is not forwarded to the BS during that round. If a scheduled health check coincides with a CH failure, global reclustering, when triggered, supersedes local replacement; otherwise, local replacement is performed at most once at that round boundary.

Unequal clustering adjusts intracluster loads across spatial regions during topology construction. EPPR–ACO reduces intercluster costs during periodic collection. Dual thresholds determine maintenance scope from runtime state. The first two modules govern how energy is consumed, while lifetime maintenance determines when the topology is updated. Together, they form a closed-loop energy-management mechanism.

### 4.2. Unequal Clustering with Distance-Adaptive Radii

UEAD adjusts competition radii according to node-to-base-station distance to regulate intracluster collection loads and the number of distant traffic sources. Nearby nodes use smaller radii to restrict the coverage of individual heads, while distant nodes use larger radii to collect data over wider areas. This establishes the spatial distribution of cluster heads and members before relay selection and route planning. The competition radius is defined as:

(14) $$R_{i}=R_{\min}+\left(R_{\max}-R_{\min}\right){\left(\frac{d_{i}}{d_{\max}}\right)}^{\gamma }$$

Here,$\;R_{i}$ is the competition radius of node $v_{i}$, and $R_{\min}$ and $R_{\max}$ are its lower and upper bounds. The parameter γ controls the nonlinear relationship between distance and radius. The distance $d_{i}=d_{i,\mathrm{BS}}$ is measured from node $v_{i}$ to the base station, and $d_{\max}$ is the maximum base-station distance among surviving nodes participating in the current clustering operation.

The radius increases with normalized distance. Smaller coverage near the base station limits member-data reception loads at individual heads, while larger coverage farther away reduces the need for multiple distant heads to transmit separate aggregated packets. The resulting cluster sizes depend on both node distribution and the greedy coverage procedure.

UEAD constructs clusters through deterministic greedy coverage. Initially, all surviving nodes are unassigned. The unassigned node with the largest competition radius becomes a cluster head, and unassigned nodes within its coverage join that cluster. Assigned nodes are then removed from further consideration. This procedure repeats until all surviving nodes have been assigned. Ties in competition radius are resolved by greater base-station distance, followed by node identifier.

UEAD uses deterministic greedy coverage to assign surviving nodes to clusters. A node not covered by any previously selected CH remains in the candidate set and can subsequently be selected as a CH; if it covers no other unassigned node, it forms a singleton cluster. Thus, every surviving node is assigned, although the procedure does not guarantee a globally optimal partition or a minimum number of CHs. Under the deployment and competition-radius settings evaluated in this study, no singleton clusters were observed, indicating that the clustering procedure achieved complete coverage without producing singleton clusters in the tested scenarios. This result is specific to the evaluated deployments: under more strongly non-uniform spatial distributions, sparse pockets may still produce singleton or very small clusters, whereas a CH may cover several nodes in denser areas. Such variations can change the number of source CHs and affect routing and maintenance overhead. UEAD does not currently include a post-clustering merge or refinement step. Route connectivity is assessed separately during route planning under the communication-range and relay constraints; a CH for which the route planner finds no feasible path is marked as route-infeasible. Density-aware radius adaptation and post-clustering refinement are potential extensions.

**Figure 2:**
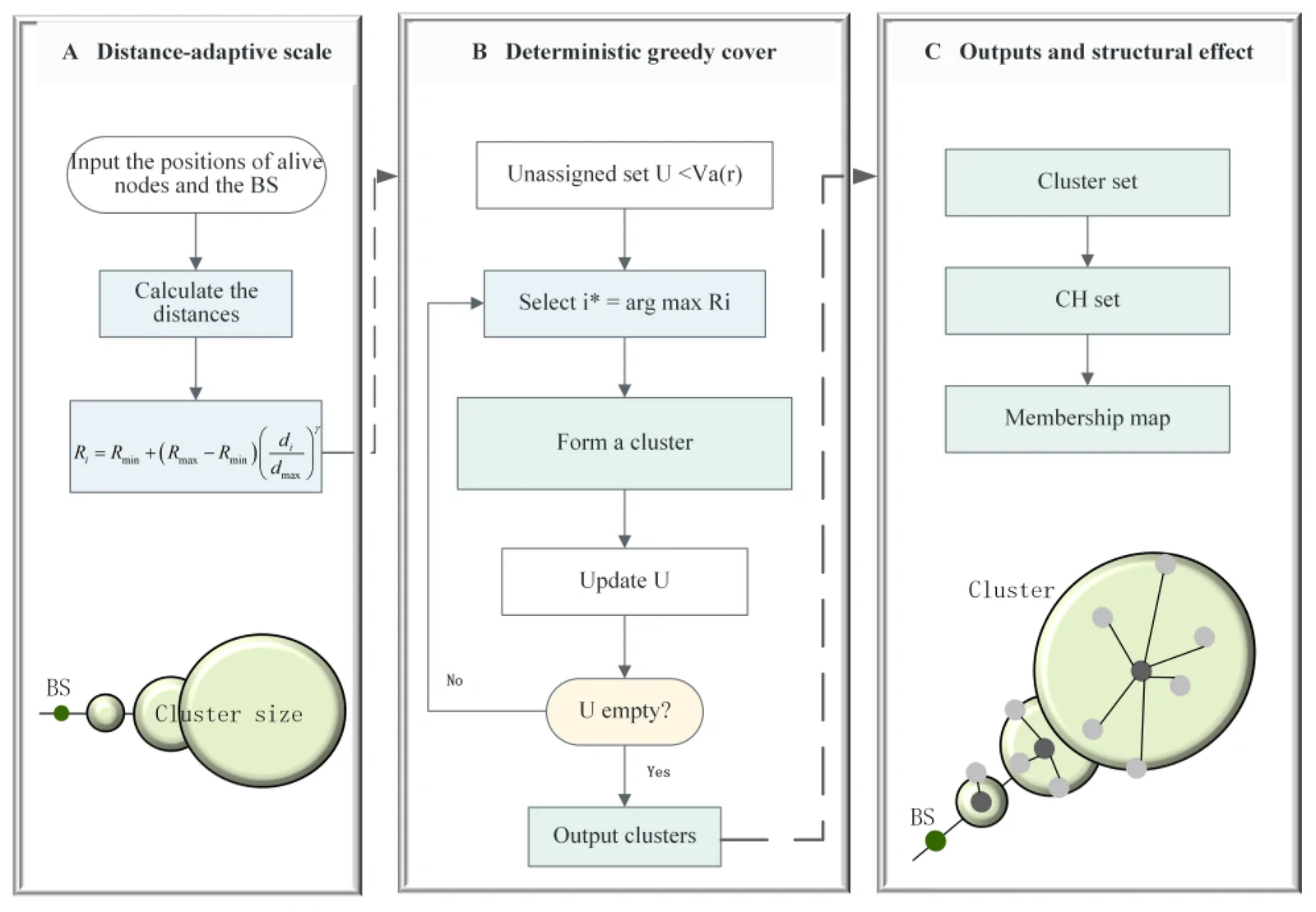
Distance-adaptive unequal clustering. The asterisk in i* identifies the node selected as the cluster head by maximizing the competition radius over the unassigned nodes. Arrows indicate the direction of processing and control flow. Box colors visually distinguish workflow elements and do not encode numerical values.

The base station performs the clustering procedure centrally and determines the cluster-head set and memberships. Unequal clustering establishes aggregation locations and cluster coverage, while EPPR–ACO subsequently selects ordinary relay nodes and constructs routes for the resulting source heads. Figure 2 illustrates the distance-adaptive clustering procedure.

### 4.3. EPPR-Initialized ACO Intercluster Multihop Routing

After cluster formation, each head aggregates member data for delivery to the base station. To reduce the communication cost of distant heads, UEAD uses EPPR-initialized ACO to construct multihop routes through ordinary relay nodes. EPPR derives node scores from the candidate topology, radio link costs, and base-station personalization. These scores initialize ACO pheromones to guide early exploration toward low-cost routes under a limited iteration budget.

#### 4.3.1. Candidate Routing Graph and Link Costs

For each source cluster head, UEAD constructs a candidate routing graph containing that head, all surviving ordinary nodes, and the base station. The source head transmits only its own aggregated data, ordinary nodes serve as candidate relays, and the base station is the destination. Other cluster heads are excluded from the graph to prevent them from handling intercluster forwarding in addition to their own data collection and aggregation duties.

Candidate links must satisfy the effective communication-range constraint. A source head or ordinary node within range of the base station can transmit to it directly. This physical communication radius determines which routing links are available, whereas the competition radius in Section 4.2 governs coverage during cluster formation.

For node i and a candidate next-hop sensor node j, the link communication cost is defined as:

(15) $$C_{ij}=E_{\mathrm{TX}}\left(l,d_{ij}\right)+E_{\mathrm{RX}}\left(l\right),$$

where l is the packet length in bits, $d_{ij}$ is the distance between nodes i and j, and $E_{TX}$ and $E_{RX}$ denote transmission and reception energy, respectively. These costs are calculated using the first-order radio model in Section 5.1.2. A smaller $C_{ij}$ indicates a lower communication energy cost for the link.

#### 4.3.2. EPPR Node-Importance Computation

Uniform initial pheromones do not encode differences in network structure among candidate links, leaving early route selection to local heuristics and stochastic exploration. UEAD incorporates additional information by computing EPPR scores on the candidate graph and mapping them to initial pheromone values. This initialization uses topology and communication costs to guide route exploration under a limited ACO iteration budget.

The transition probability from node i to candidate node j is defined as:

(16) $$P_{ij}=\frac{C_{ij}^{-1}}{\sum_{k\in N\left(i\right)}C_{ik}^{-1}},$$

where $N_{i}$ is the set of candidate next-hop nodes for node i. Links with lower communication energy costs are assigned higher transition probabilities.

EPPR is link communication-cost-aware rather than residual-node-energy-aware. Its transition probabilities are computed from the transmission and reception costs of each candidate link under the first-order radio model, which depend on packet length and link distance. Residual node energy does not enter the transition matrix. Dead nodes are excluded from the candidate graph, and residual energy changes the active topology through cluster-health evaluation, cluster-head rotation, and global reclustering, as described in Section 4.4.

Using a personalization vector concentrated at the base station, EPPR updates the node scores as follows:

(17) $$r_{j}^{\left(t+1\right)}=\delta \underset{i}{\sum }P_{ij}r_{i}^{\left(t\right)}+\left(1-\delta \right)v_{j},$$

EPPR uses a bidirectional auxiliary adjacency graph corresponding to physical reachability, allowing base-station personalization to propagate to ordinary nodes along low-cost connections. Reverse adjacency from the base station is used only for centrality computation; it does not represent data-phase transmissions or incur base-station communication costs. The personalization vector is one at the base station and zero elsewhere and also initializes the score vector. A row with no available neighbor is replaced by the personalization vector. Iteration stops when the L1 difference between consecutive score vectors is at most the convergence threshold, or the preset iteration limit is reached.

In the simulations, the damping factor was set to δ = 0.8. EPPR iteration stopped when the L1 difference between consecutive score vectors fell below 1 × 10^−9^ or when 200 iterations had been reached, whichever occurred first.

Here, $r_{j}^{\left(t\right)}$ is node j’s EPPR score at iteration t, δ is the damping factor, and $v_{j}$ denotes the personalization vector component. The base-station component is weighted more heavily, while other components are zero. At convergence, higher-scoring nodes generally lie within low-cost forwarding structures connected to the base station. EPPR supplies a prior rather than final routes. The initial pheromone on link i, j is:

(18) $$\tau_{ij}\left(0\right)=\tau_{0}r_{j},$$

where $\tau_{0}$ is the baseline pheromone intensity. This initialization assigns higher pheromone values to links leading to higher-scoring nodes, increasing their initial selection probabilities when other factors are equal. EPPR thus provides a graph-based prior that complements the local heuristic during early route exploration.

#### 4.3.3. ACO Path Construction and Pheromone Updates

When ant k is at node i, its probability of selecting next-hop node j from feasible set $A_{i}^{k}$ is:

(19) $$p_{ij}^{k}=\frac{\tau_{ij}^{\alpha }\eta_{ij}^{\beta }}{\sum_{s\in A_{i}^{k}}\tau_{is}^{\alpha }\eta_{is}^{\beta }}$$

Here, α and β control the influence of pheromones and heuristic information, respectively, and $\eta_{ij}=1/C_{ij}$ is the link-energy heuristic. Candidates must satisfy distance, node-role, and loop-free constraints. After a search iteration, pheromones are updated by:

(20) $$\tau_{ij}^{\left(t+1\right)}\;=\;\left(1-\rho \right)\tau_{ij}^{t}\;+\;\Delta \tau_{ij}^{t}$$

Here, ρ is the evaporation coefficient. At iteration t, the pheromone increment is Δτ_ij(t) = 1/C_best(t) if link (i,j) belongs to the best-so-far feasible route P_best(t), and Δτ_ij(t) = 0 otherwise. P_best(t) is the lowest-cost feasible route found up to iteration t, and C_best(t) is its total route cost under the link-cost model in Section 4.3.1, including the final transmission from the last sensor node to the BS. Thus, each update reinforces the best-so-far route; pheromone is not deposited independently by every ant.

#### 4.3.4. Multipath Planning Constraints

To limit the concentration of forwarding traffic, UEAD plans routes in descending order of cluster-head-to-base-station distance and assigns ordinary relays exclusively to individual source routes. An ordinary node allocated to one active route cannot relay for another cluster head. Nodes cannot be revisited within a route, ensuring loop-free path construction.

For each source CH, the planner performs at most four EPPR–ACO searches in total: one initial search and up to three restarts. The default lifetime configuration uses 20 ants and 50 iterations per search, giving an upper bound of 4000 ant route constructions per source CH (4 × 20 × 50). A restart is attempted only when the preceding search finds no feasible route, and planning stops when a search returns a feasible route. The iteration-budget and equal-total-time experiments in Section 5.4.5 and Section 5.4.6 use the budgets stated for those experiments.

**Figure 3:**
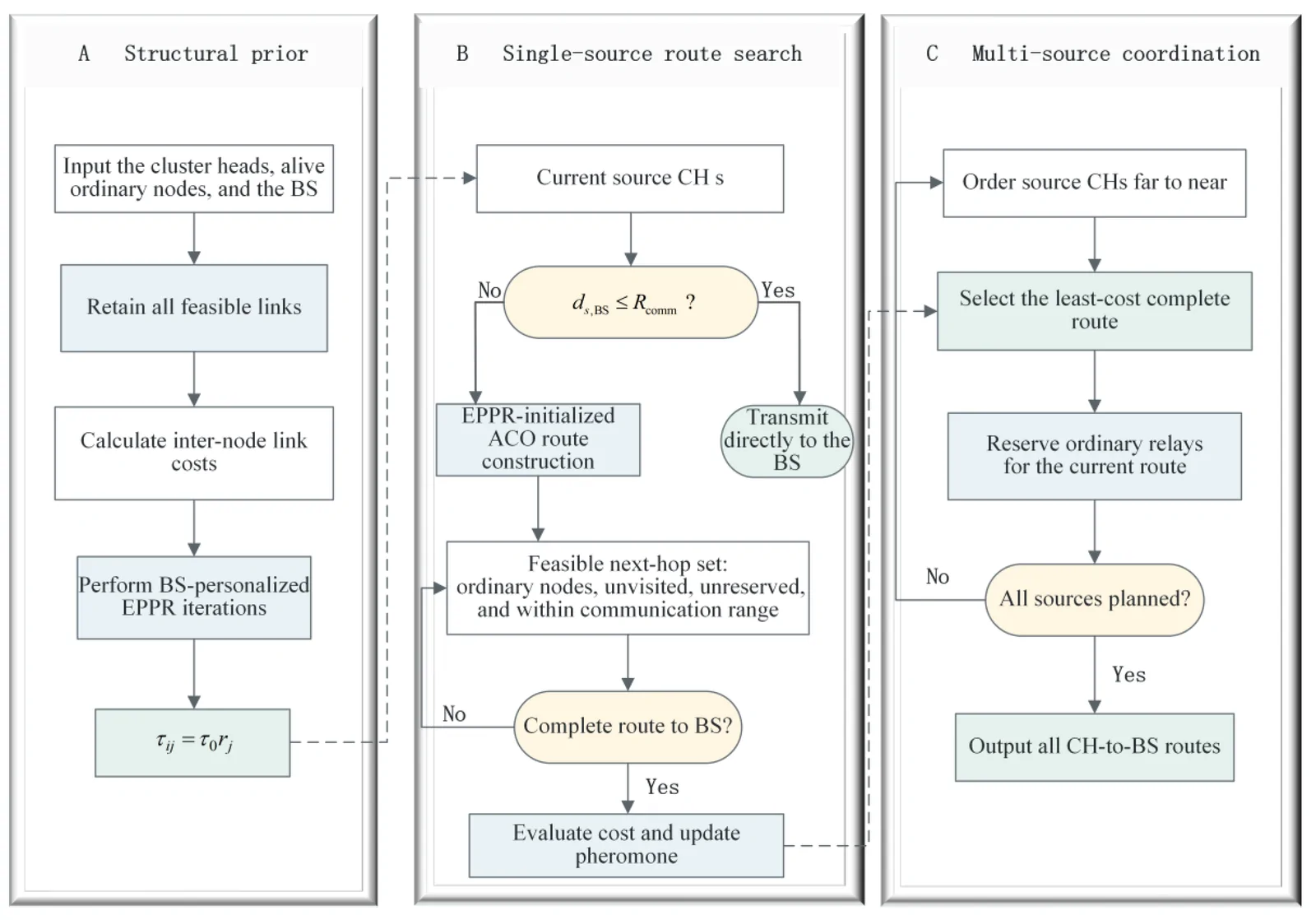
EPPR-initialized ACO multihop routing. Arrows indicate the direction of processing and control flow. Box colors visually distinguish workflow elements and do not encode numerical values.

If all four fail for any source, initialization stops and the complete strict route set is marked infeasible. No out-of-range link or path violating node-role, loop-free, or relay-exclusivity constraints is inserted. Only in the larger-area test in Section 5.8, a BFS fallback is applied after strict failure. It preserves link-range, ordinary-node relay, cluster-head exclusion, and loop-free constraints, but allows relay reuse; if BFS also fails, the topology is recorded as lacking a complete route set. This fallback is not used in standard lifetime results. Figure 3 summarizes EPPR-initialized ACO route construction.

### 4.4. Dual-Threshold Cluster-Head Rotation and Dynamic Reclustering

Frequent reclustering incurs control overhead, while retaining the same clusters for too long can place sustained loads on particular heads. UEAD combines periodic health checks with state-driven maintenance to address this tradeoff. A cluster-health threshold identifies degraded clusters, and an unhealthy-cluster proportion threshold determines whether to rotate their heads locally or rebuild the network’s cluster structure.

#### 4.4.1. Cluster-Health Computation

During steady-state operation, cluster members periodically report their residual energy to their heads, which summarize the cluster state and forward it to the base station. The base station evaluates cluster health using the mean residual energy and its dispersion among surviving nodes. For cluster c, the health metric is defined as:

(21) $$H_{c}=\frac{{\bar{E}}_{c}}{E_{\mathrm{ref}}}\exp\left(-\frac{\sigma_{c}}{{\bar{E}}_{c}}\right),$$

where ${\bar{E}}_{c}$ is the mean residual energy of surviving nodes in cluster c, $\sigma_{c}$ is the standard deviation of their residual energy, and $E_{\mathrm{ref}}$ is the reference energy.

The metric captures both the cluster’s remaining energy and its distribution among surviving nodes. $H_{c}$ is higher when mean residual energy is high and relative energy dispersion is low. It decreases as mean energy declines or relative dispersion increases, providing a broader assessment of cluster condition than the cluster head’s residual energy alone.

#### 4.4.2. Local Cluster-Head Rotation

Let $T_{H}$ denote the cluster-health threshold. A cluster c satisfying $H_{c}<T_{H}$ is added to the unhealthy-cluster set at the current check. Rotation is deferred until the base station evaluates the global maintenance criterion. If global reclustering is not triggered, the surviving ordinary member with the highest residual energy is selected as the replacement head. Ties are resolved first by shorter distance to the base station and then by smaller node identifier.

Local rotation replaces the cluster head while preserving cluster membership. If the former head remains alive, it becomes an ordinary member. Before promoting the replacement, the base station checks whether that member is currently assigned as an ordinary relay on another active source route. Because cluster heads cannot serve as relays, the former head’s route and every other route that uses the replacement as an intermediate relay are invalidated, and the relay reservations for those routes are released. Routes that do not use the replacement are retained, with their relay reservations preserved. The base station then promotes the replacement and replans the new head’s route and all invalidated source routes under the updated node roles and exclusive-relay constraint, processing them in descending order of source-head-to-base-station distance. If a route cannot be reconstructed, it remains invalid and its aggregated data are omitted from forwarding to the base station for that round, while member-to-head collection can continue; the route is retried at the next route-repair step. The base station notifies surviving cluster members and sends scheduling notifications for the reconfigured routes. The energy consumed in receiving these notifications is included in control energy. When a CH dies, this same replacement procedure is invoked before the next round’s member-data collection, independently of the periodic health-check schedule; the dead former CH is excluded from the surviving-member selection.

When several clusters require local rotation, they are processed in ascending order of health, with ties resolved by cluster identifier. If a cluster has no surviving ordinary member eligible to replace its head, it is dissolved and its surviving nodes join the nearest valid cluster. The receiving cluster is identified by the minimum distance from the former head’s position to any surviving node in another valid cluster. Any resulting membership or source-head changes trigger updates to the affected routes, while other valid clusters and routes remain unchanged.

#### 4.4.3. Global Dynamic Reclustering

Local rotation addresses degradation confined to a limited number of clusters. When many clusters are unhealthy, replacing their heads individually may be insufficient to address the broader load imbalance. The base station therefore calculates the proportion of unhealthy clusters before initiating any local action and uses it to determine whether local rotation or global reclustering is required:

(22) $$P_{\mathrm{low}}=\frac{N_{\mathrm{low}}}{N_{c}}$$

Here, $N_{\mathrm{low}}$ is the number of clusters satisfying $H_{c}<T_{H}$, and $N_{c}$ is the current number of valid clusters. Let $T_{P}$ denote the global maintenance threshold. When $P_{\mathrm{low}}\geq T_{P}$, the base station initiates global reclustering and cancels any pending local rotations at that check. All existing routes and relay allocations are released, surviving nodes are marked unassigned, and unequal clustering and EPPR–ACO route planning are repeated.

**Figure 4:**
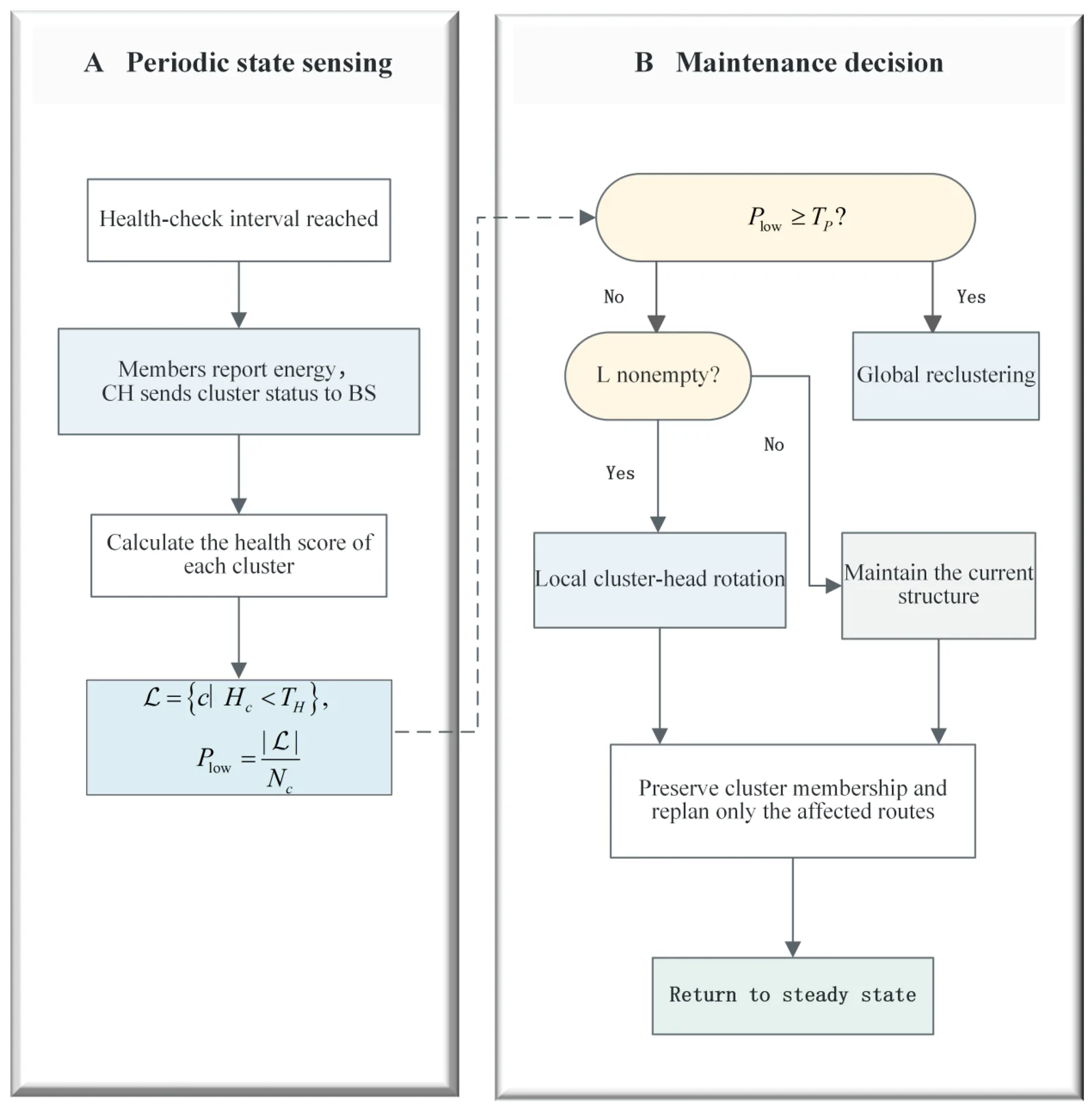
Dual-threshold network maintenance. Arrows indicate the direction of processing and control flow. Box colors visually distinguish workflow elements and do not encode numerical values.

Local rotation and failure-driven repair update the affected routes, whereas global reclustering rebuilds the complete path set. These actions adapt the scope of topology maintenance to the observed network state and limit unnecessary updates to otherwise valid structures. Figure 4 summarizes the dual-threshold maintenance procedure.

### 4.5. UEAD Operating Procedure

UEAD combines distance-driven unequal clustering, EPPR-initialized ACO multihop routing, and dual-threshold maintenance. Algorithm 1 summarizes its operation.
**Algorithm 1.**  **UEAD operating procedure**Input: node set, base-station position, clustering and communication parameters, EPPR–ACO parameters, health-check interval, and dual thresholds.Output: cluster structure, head-to-base-station paths, and node energy state in each round.01      Initialize node positions, residual energies, and survival states.02      Compute node-to-base-station distances and unequal competition radii.03      Construct unequal clusters using deterministic greedy coverage.04      Determine cluster heads, memberships, and the surviving ordinary-node set.05      Construct candidate routing graphs subject to communication-range, node-role, and loop-free constraints.06      Compute EPPR scores from first-order radio-model link communication costs.07      Initialize ACO pheromones using normalized EPPR scores.08      **for each source CH in descending order of distance to the BS do**    09      routeFound ← false    10      **for attempt ← 1 to 4 do**      11      Run one EPPR–ACO search using the current relay reservations.      12      **if a feasible route is found then**        13      Commit the route and reserve its ordinary relay nodes.        14      routeFound ← true        15      **break**      16      **end if**    17      **end for**    18      **if routeFound = false then**      19      Stop strict initialization and mark the complete exclusive-relay route set infeasible.      20      **return**    21      **end if**22      **end for**23      **while at least one sensor node remains alive do**    24      Complete pending failure-triggered role and route updates before member-data transmission.    25      Transmit member data to the corresponding CHs.    26      **for each live CH do**      27      Aggregate the received member data.      28      **if a valid route to the BS is available then**        29      Transmit the aggregate along the established route.      30      **else**        31      Omit this source from BS forwarding for the current round.      32      **end if**    33      **end for**    34      Update energy and survival states; energy-deficient nodes complete the current packet and die at round end.    35      At round end, identify dead nodes and classify each failure as a CH failure or an ordinary-relay failure.    36      globalReclustering ← false    37      **if a scheduled health check is due then**      38      Evaluate cluster health and compute the proportion of unhealthy clusters.      39      **if the proportion reaches the global threshold then**        40      Recluster globally and rebuild all routes.        41      globalReclustering ← true        42      **else**          43      Apply health-driven local rotation in unhealthy clusters using the Section 4.4.2 replacement procedure.        44      **end if**    45      **end if**    46      **if globalReclustering = false then**      47      **for each failed CH not already replaced by health-driven rotation do**        48      Apply local replacement before the next round’s member-data collection.      49      **end for**      50      **for each dead ordinary relay do**        51      Invalidate only paths containing the relay; preserve cluster-head and membership assignments.      52      **end for**      53      Release reservations for affected paths; retain unaffected paths and reservations.      54      Replan the new-head and invalidated routes before the next round.      55      **if a route cannot be restored then**        56      Keep the route invalid; omit that source only from BS forwarding for this round.      57      **end if**    58      **end if**59      **end while**

Initialization comprises unequal clustering, EPPR computation, and ACO route search. Local maintenance replans affected routes, whereas global reclustering repeats cluster formation and route planning for all source heads. The base station performs these computations; sensor-node energy accounting includes the associated reporting and notification exchanges, as specified in Section 5.1.2.

### 4.6. Protocol Summary

UEAD coordinates cluster formation, intercluster routing, and maintenance throughout network operation. Distance-adaptive clustering determines memberships and source heads, EPPR initializes constrained ACO route search, and cluster health determines the scope of subsequent structural updates. Failure-driven repairs operate independently of periodic health checks. Section 5 evaluates these mechanisms and the complete protocol under the stated simulation assumptions.

## 5. Results and Discussion

The evaluation first examines parameter selection and visualizes clustering and route construction. Route-quality tests compare ACO and EPPR–ACO under equal iteration and total planning-time budgets. Module ablations and comparisons with Standard LEACH and DUCISCA assess lifetime performance. Further experiments examine base-station placement, expanded-area routing with relay-sharing fallback, network-size effects on runtime and control energy, and sensitivity to no, 50% partial, and full aggregation.

### 5.1. Simulation Environment and Experimental Setup

#### 5.1.1. Simulation Environment and Network Model

Experiments were conducted in MATLAB R2025b using 200 sensor nodes randomly deployed in a 200 m×200 m area. Unless otherwise specified in the base-station location experiments, the base station was positioned at the lower-left corner $\left(0,\;0\right)$. All nodes had identical hardware capabilities and an initial energy of 1 J. They remained stationary throughout each simulation and received no external energy replenishment.

The primary lifetime comparisons used 200 sensor nodes in a 200 m × 200 m area. The expanded-area feasibility test in Section 5.8 used 1000 nodes in a 447.2 m × 447.2 m area across five topology seeds, preserving approximately the same nominal node density. Its relay-sharing fallback and route-set results are reported separately from the lifetime comparisons.

#### 5.1.2. Wireless Communication and Energy Model

A classical first-order radio model describes sensor transmission and reception. Transmitting an l-bit packet over distance d consumes electronics and amplifier energy. Depending on the channel-distance threshold $d_{0}$, transmission energy is:

(23) $$E_{\mathrm{TX}}\left(l,\;d\right)=\left\{\begin{array}{cc} lE_{\mathrm{elec}}+l\varepsilon_{\mathrm{fs}}d^{2}, & d<d_{0},\\ lE_{\mathrm{elec}}+l\varepsilon_{\mathrm{mp}}d^{4}, & d\geq d_{0}, \end{array}\right.$$

Here, $E_{\mathrm{elec}}$ is electronics energy per bit, and $\varepsilon_{\mathrm{fs}}$ and $\varepsilon_{\mathrm{mp}}$ are amplifier coefficients for free-space and multipath channels, respectively. The crossover distance is:

(24) $$d_{0}=\sqrt{\frac{\varepsilon_{\mathrm{fs}}}{\varepsilon_{\mathrm{mp}}}}.$$

Under the selected parameters, $d_{0}$ is approximately 87.706 m. Because UEAD limits single-hop distances to the effective communication radius, most intra- and intercluster links operate in the free-space region, avoiding the multipath amplifier cost that grows with $d^{4}$.

Receiving an l-bit packet consumes:

(25) $$E_{\mathrm{RX}}\left(l\right)=lE_{\mathrm{elec}}.$$

Data packets contain 4000 bits, and control packets contain 100 bits. Under the default full-aggregation setting used in the three-protocol comparisons, each cluster head combines the readings received from its members with its own reading into one 4000-bit output packet and sends it along the planned route. Ordinary relays incur both reception and retransmission costs. The radio cost of each transmission and reception is calculated using the packet payload specified for that aggregation condition. Aggregation-computation energy is not charged separately; this convention is used consistently across aggregation conditions and the baseline protocols. Base-station reception and computation, including EPPR, ACO search, and maintenance decisions, are excluded from sensor-node energy accounting. Sensor-node communication costs associated with health reporting and the reception of rotation, reclustering, and route-scheduling notifications are included in control energy.

The simulation adopts a packet-completion convention when a node has insufficient residual energy for an ongoing communication operation. The operation is allowed to finish, the node’s recorded energy is set to zero at the end of the round, and the node is excluded from subsequent rounds. The difference between the modeled communication cost and the energy actually deducted is recorded separately as an energy deficit. This convention defines packet handling in the simulation and does not imply that a physical node can operate beyond its available energy.

For the aggregation-sensitivity analysis, each source generates a 4000-bit data packet. Under no aggregation, the cluster head forwards the packets from all sources in its cluster. Under 50% partial aggregation, half of the member-data payload is removed before forwarding. Under full aggregation, all readings are combined into a single 4000-bit packet. Member-to-cluster-head transmissions and receptions use the original 4000-bit packets, whereas inter-cluster transmission and relay costs are calculated using the payload size after aggregation. Aggregation-computation energy is not charged separately.

#### 5.1.3. UEAD Parameters and Control Settings

UEAD uses distance-dependent competition radii with lower and upper bounds of 55 and 65 m, respectively. Radii increase with distance to the base station. Smaller coverage near the base station limits member-data reception loads at individual heads, while broader coverage farther away reduces the need for multiple distant source heads. Each head transmits only its own aggregated data. Ordinary relays forward intercluster traffic. Standard lifetime simulations use strict route assignment, in which an ordinary relay is reserved for at most one active route. The separate Section 5.8 larger-area feasibility test applies relay sharing only as a fallback after strict EPPR–ACO search fails.

Intercluster routing uses EPPR-initialized ACO with pheromone weight 0.6, heuristic weight 2.0, and evaporation coefficient 0.6. The EPPR model and bounded route-search procedure are defined in Section 4.3. Each source head receives at most four searches: one initial attempt and up to three restarts. Table 1 lists the default ant and iteration budgets; Section 5.4.5 and Section 5.4.6 specify the budgets used in the time- and iteration-constrained comparisons.

Health-driven maintenance uses the cluster-health threshold $T_{H}$ and the unhealthy-cluster proportion threshold $T_{P}$. The sensitivity experiments led to the following settings:

(26) $$T_{H}=0.8,\;T_{P}=0.5.$$

Cluster health is evaluated every 50 rounds. At each check, the base station first determines the proportion of clusters with health below $T_{H}$. If this proportion reaches or exceeds $T_{P}$, global reclustering rebuilds the clusters and routes for all surviving nodes. Otherwise, local rotation is performed only in unhealthy clusters, replacing their heads and updating the affected routes while preserving memberships. If no clusters are unhealthy, the current structure is retained. These mutually exclusive rules give priority to global reclustering and limit local maintenance to the affected clusters. This 50-round interval governs energy-based health assessment; failure-triggered CH replacement and ordinary-relay route repair are completed before the next round and are not deferred to this schedule.

Table 1 summarizes the main simulation parameters.

#### 5.1.4. Baselines and Evaluation Metrics

Standard LEACH and DUCISCA are used as baseline protocols. Standard LEACH employs probabilistic cluster-head election and direct transmission from cluster heads to the base station. DUCISCA is implemented using the same radio energy model, packet lengths, and node-death rules as UEAD and Standard LEACH. For the three-protocol comparisons, UEAD, Standard LEACH, and DUCISCA use the same full-aggregation setting: each cluster head forwards one 4000-bit packet per collection round, and no aggregation-computation energy is charged. No, partial, and full aggregation are varied only in the separate UEAD sensitivity analysis. All comparisons use identical node deployments and initial energy settings. Control energy is accounted for separately for each protocol according to the control messages transmitted and received during execution.

All control packets are assumed to contain 100 bits. In UEAD, residual-energy reports are sent at each health check, which occurs every 50 rounds. Notifications for cluster-head rotation, global reclustering, route scheduling, and route repair are counted only when the corresponding event occurs. In Standard LEACH, cluster-head advertisements, member join requests, and TDMA schedules are counted during cluster formation in each round. For DUCISCA, event-based accounting includes candidate-head competition broadcasts, base-station cluster-head selection messages, winning-head broadcasts, cluster-head announcements, and member join requests when clustering occurs; no DUCISCA control-message cost is assigned to rounds without clustering. Because the adapted DUCISCA implementation selects cluster heads centrally, these events are reconstructed from its candidate-head, selected-head, and membership states.

Four protocol variants are used for module ablation:

1. UC-ACO-D: unequal clustering, ordinary ACO multihop routing, and dynamic reclustering;
2. UC-EA-F: unequal clustering, EPPR–ACO multihop routing, and fixed-period reclustering;
3. UEAD: unequal clustering, EPPR–ACO multihop routing, and dynamic reclustering;
4. UEAD-Uniform: uniform clustering, EPPR–ACO multihop routing, and dynamic reclustering.

UC-ACO-D replaces EPPR-based pheromone initialization with uniform initialization to isolate the contribution of the graph-based prior. UC-EA-F replaces health-driven maintenance with full-network reclustering every 50 rounds. This uses the same 50-round cadence as the UEAD health assessment, but triggers global reconstruction on schedule rather than according to the health thresholds. UEAD-Uniform replaces distance-adaptive unequal clustering with uniform clustering using a fixed 60 m competition radius. The complete UEAD protocol combines distance-adaptive unequal clustering, EPPR-initialized ACO routing, and dual-threshold maintenance.

Network lifetime is characterized by first-node death (FND), half-node death (HND), and last-node death (LND). FND marks the end of the period during which all nodes remain alive. For the 200-node network, HND is the round in which the 100th node dies, indicating the loss of half the initial node population. LND is the round in which the final surviving node dies. These metrics describe the onset, midpoint, and completion of node loss and are defined as follows:

(27) $$R_{\mathrm{FND}}=\min\left\{r:N_{\mathrm{dead}}\left(r\right)\geq 1\right\}$$

(28) $$R_{\mathrm{HND}}=\min\left\{r:N_{\mathrm{dead}}\left(r\right)\geq N/2\right\}$$

(29) $$R_{\mathrm{LND}}=\min\left\{r:N_{\mathrm{dead}}\left(r\right)=N\right\}$$

where $N_{\mathrm{dead}}\left(r\right)$ is the cumulative number of dead nodes at the end of round r.

Additional metrics include total residual energy, mean hop count, total path length, complete-routing success rate, mean transmission energy, control energy, and the number of global reclustering events. Energy dispersion among surviving nodes is measured by the coefficient of variation of their residual energy, defined as follows:

(30) $${\mathrm{CV}}_{E}\left(r\right)=\frac{\sigma_{E}\left(r\right)}{\mu_{E}\left(r\right)},$$

where $\mu_{E}\left(r\right)$ and $\sigma_{E}\left(r\right)$ are the mean and standard deviation of residual energy among surviving nodes in round r, respectively. A lower ${\mathrm{CV}}_{E}\left(r\right)$ indicates less relative dispersion and thus a more balanced distribution of residual energy among those nodes.

### 5.2. Parameter Sensitivity and Selection

UEAD parameters influence routing feasibility, communication energy, network lifetime, and control overhead. Parameter selection therefore proceeds in two stages. First, ACO settings are evaluated using complete-routing success rate and mean transmission energy, with energy costs compared among settings that satisfy the routing feasibility criterion. Cluster-health and unhealthy-cluster proportion thresholds are then assessed jointly using FND, HND, LND, control energy, and the number of global reclustering events. This procedure considers both routing performance and the lifetime–overhead tradeoff in maintenance.

#### 5.2.1. EPPR–ACO Routing-Parameter Sensitivity

ACO search behavior depends on the pheromone weight α, heuristic weight β, and evaporation coefficient ρ. The parameter α controls the influence of pheromone values on next-hop selection, β determines the influence of heuristic information, and ρ governs pheromone decay between iterations.

To examine their interactions, response surfaces are constructed for the α−β, α−ρ, and β−ρ parameter pairs, with the third parameter held fixed in each analysis. Complete-routing success indicates whether valid routes are obtained for every cluster head requiring route planning. Mean transmission energy measures the communication cost of successfully constructed path sets.

**Figure 5:**
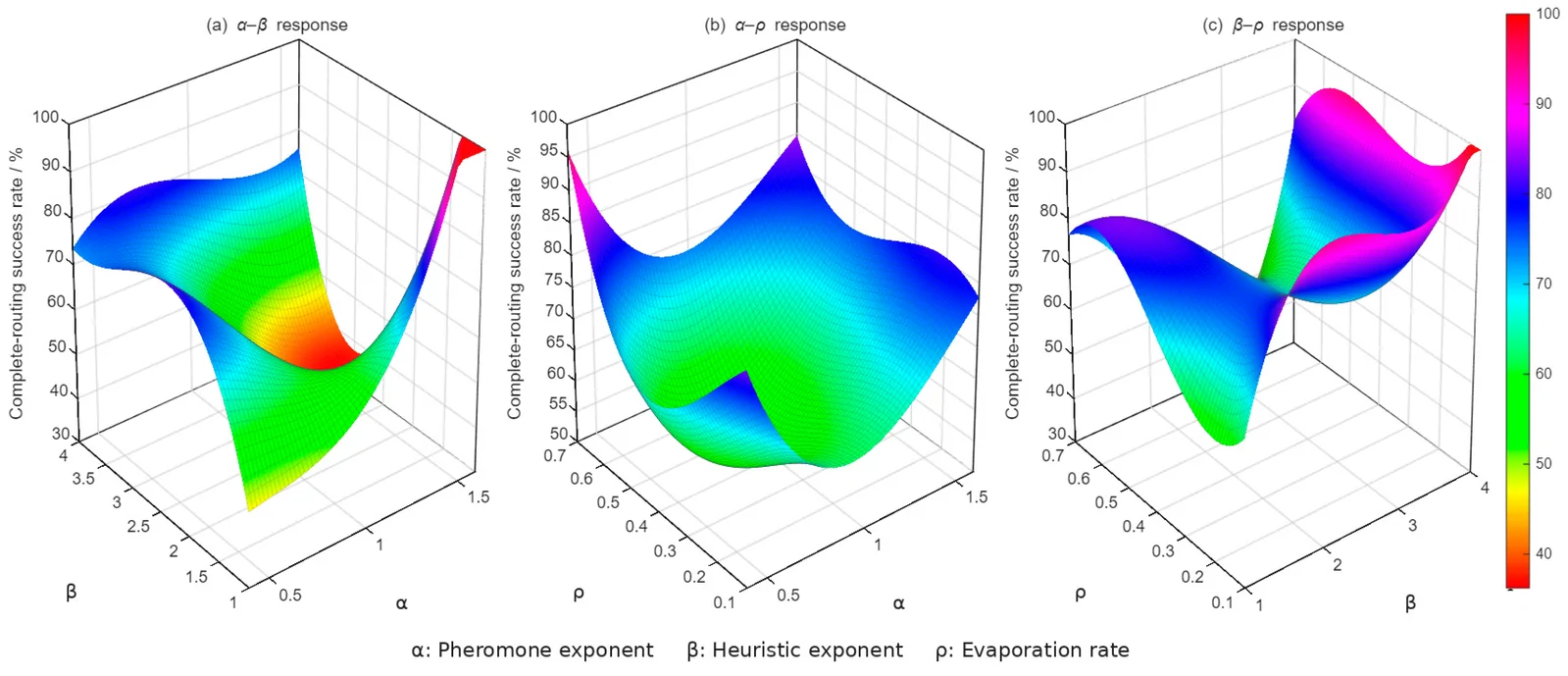
Routing success across EPPR–ACO parameter settings.

Figure 5 shows that complete-routing success varies with the combination of pheromone weight, heuristic weight, and evaporation coefficient. Lower success rates in some parameter regions suggest that an imbalance between reinforcement and exploration can limit the search for feasible routes. Strong preferences for particular candidates may reduce the exploration of alternatives within the available iteration budget.

A large α strengthens the influence of existing pheromone differences and may reinforce early path preferences. A large β places greater emphasis on local link costs, which do not fully capture downstream connectivity or cumulative relay load. With a small ρ, pheromone values persist longer and may slow adaptation; with a large ρ, useful pheromone information decays more rapidly. Parameter selection must therefore balance these effects to make effective use of EPPR initialization while preserving exploration.

**Figure 6:**
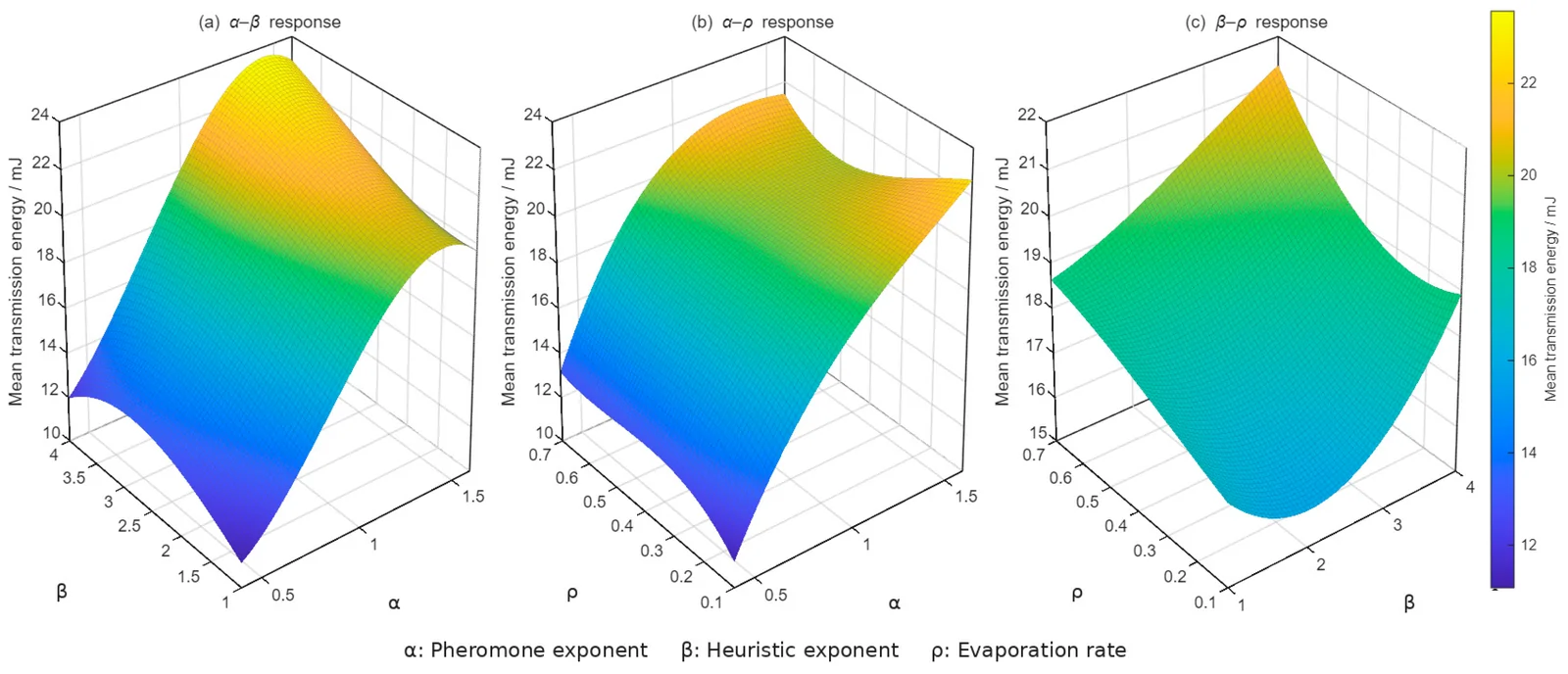
Transmission energy across EPPR–ACO parameter settings.

Figure 6 shows that mean transmission energy varies nonlinearly with α, β, and ρ. Increasing the heuristic weight can favor lower-cost links, but strong local preferences do not necessarily produce a low-cost complete route. Pheromone reinforcement helps retain favorable path choices, although excessive reliance on those choices may restrict exploration of alternatives.

Parameter selection first prioritizes complete routing. Among settings that satisfy this criterion, communication energy is used to identify the preferred combination. The selected pheromone weight, heuristic weight, and evaporation coefficient are:

(31) α=0.6,  β=2.0,  ρ=0.6.

This combination lies within a parameter region with consistently high complete-routing success and low mean transmission energy, meeting the routing feasibility criterion while keeping communication costs low. These settings are retained for subsequent mechanism validation, ablation experiments, and protocol comparisons.

#### 5.2.2. Effects of Dual Thresholds on Lifetime

UEAD coordinates local cluster-head rotation and global reclustering through $T_{H}$ and $T_{P}$. The cluster-health threshold $T_{H}$ identifies unhealthy clusters, while the proportion threshold $T_{P}$ determines when global reclustering takes priority over local rotation. A higher $T_{H}$ classifies clusters as unhealthy earlier and can increase maintenance frequency; a lower value may delay intervention. In contrast, a lower $T_{P}$ triggers global reclustering at a smaller unhealthy-cluster proportion, whereas a higher value favors local maintenance until more clusters deteriorate. The two thresholds therefore jointly determine maintenance timing, scope, and control overhead.

The following threshold values are considered:

(32) $$T_{H}\in \left\{0.5,\;0.6,\;0.7,\;0.8,\;0.9\right\}$$

(33) $$T_{P}\in \left\{0.2,\;0.35,\;0.5,\;0.65,\;0.8\right\}$$

**Figure 7:**
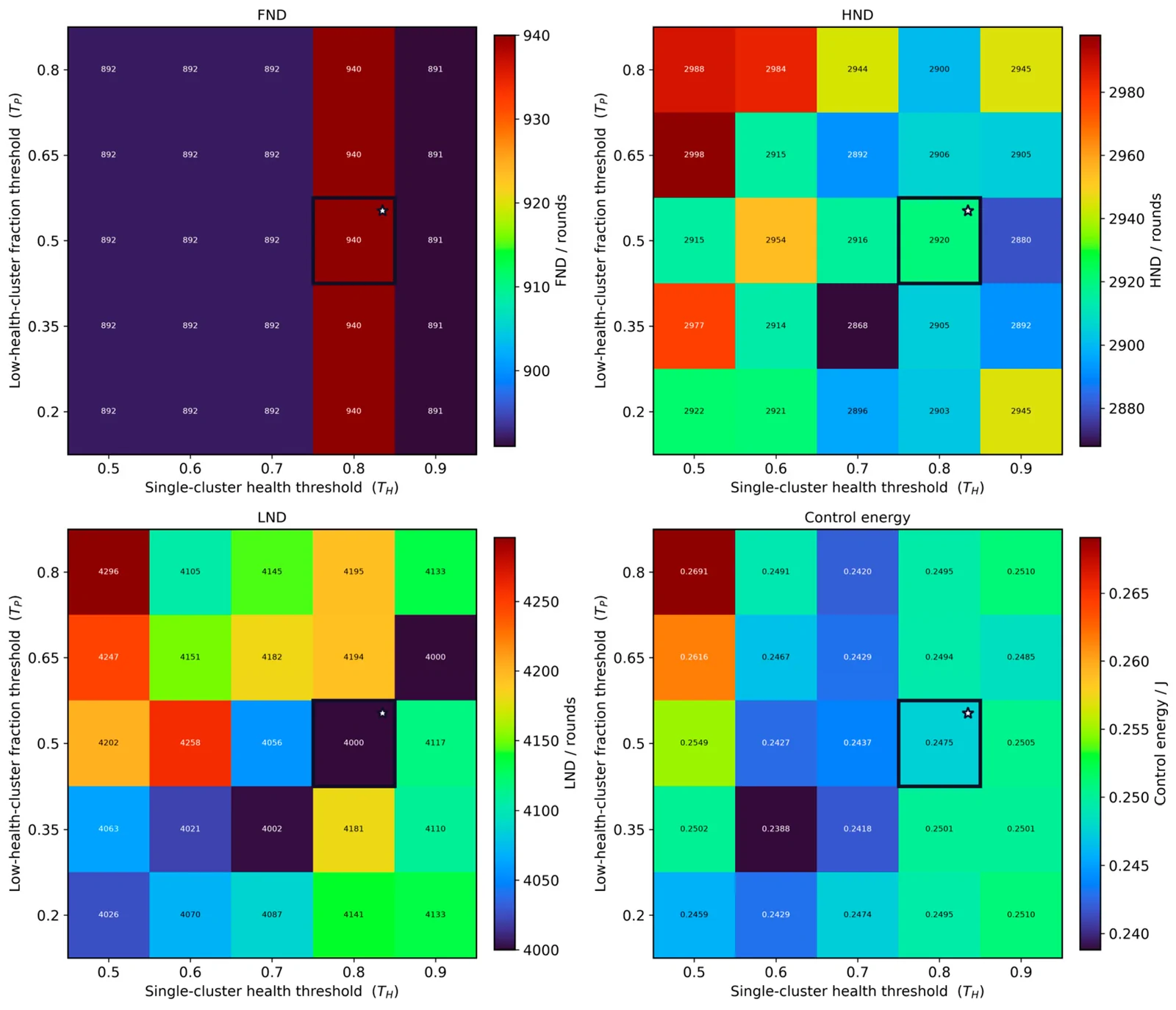
Lifetime and control energy across maintenance thresholds. The star and outlined cell identify the selected threshold combination, *T_H_* = 0.8 and *T_P_* = 0.5. Cell colors and numerical labels represent the metric values indicated by the color bars.

The two sets of threshold values yield 25 combinations. FND, HND, LND, and cumulative control energy are recorded for each combination and presented in Figure 7.

**Figure 8:**
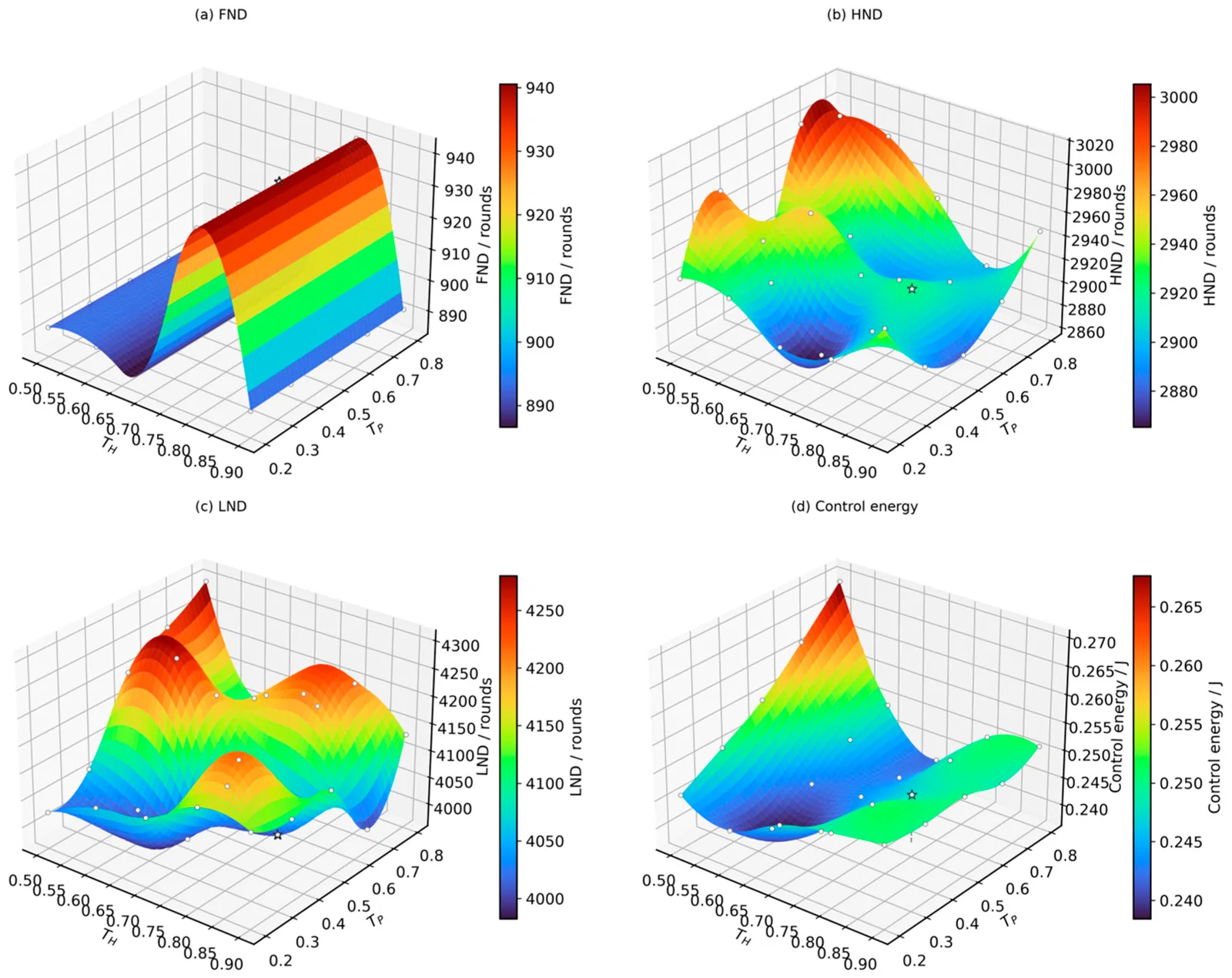
Response surfaces for maintenance thresholds. Circular markers indicate the evaluated threshold combinations, and the star marks the selected setting, *T_H_* = 0.8 and *T_P_* = 0.5. Surface colors represent the metric values indicated by the color bars.

Within the tested range, FND is more sensitive to the cluster-health threshold $T_{H}$ than to the unhealthy-cluster proportion threshold $T_{P}$. FND remains near 892 rounds for $T_{H}$ values from 0.5 to 0.7. At $T_{H}=0.8$, it reaches 940 rounds for every tested $T_{P}$, but falls to 891 rounds when $T_{H}$ increases to 0.9. This pattern suggests that earlier detection can help prevent premature node loss, while further increases in maintenance frequency may introduce control and route-update costs that offset the benefit. Figure 8 presents the response surfaces for these threshold combinations.

The limited variation in FND across $T_{P}$ values is consistent with early degradation being concentrated in a small number of clusters. Under these conditions, local rotation can affect the first death more directly than global reconstruction. The proportion threshold has a greater influence on HND and LND, suggesting that the choice between local and global maintenance becomes more consequential as degradation spreads.

HND ranges from 2868 to 2998 rounds. The maximum occurs at $T_{H}=0.5$ and $T_{P}=0.65$, although FND is only 892 rounds for this combination. Among settings with $T_{H}=0.8$, $T_{P}=0.5$ produces the highest HND, at 2920 rounds. This combination therefore provides the longest midlife survival among the settings that achieve the maximum FND.

LND ranges from 4000 to 4296 rounds. Its maximum occurs at $T_{H}=0.5$ and $T_{P}=0.8$, again with an FND of 892 rounds. The different settings that maximize FND and LND reveal a tradeoff between delaying the first failure and extending the survival of the final nodes. A longer tail does not necessarily indicate better preservation of the initial sensing population.

For periodic sensing, early node loss can reduce spatial coverage or data completeness, depending on deployment redundancy. Parameter selection therefore gives precedence to the period during which all nodes remain alive, then considers HND alongside control overhead and tail lifetime. LND is retained as an evaluation metric but does not determine the selected thresholds on its own.

#### 5.2.3. Effects on Control Overhead and Reclustering Frequency

The thresholds affect maintenance overhead as well as network lifetime. Figure 7 shows cumulative control energy ranging from 0.2388 to 0.2691 J. Although these values are small relative to the initial network energy, they reveal differences in maintenance costs across threshold combinations.

**Figure 9:**
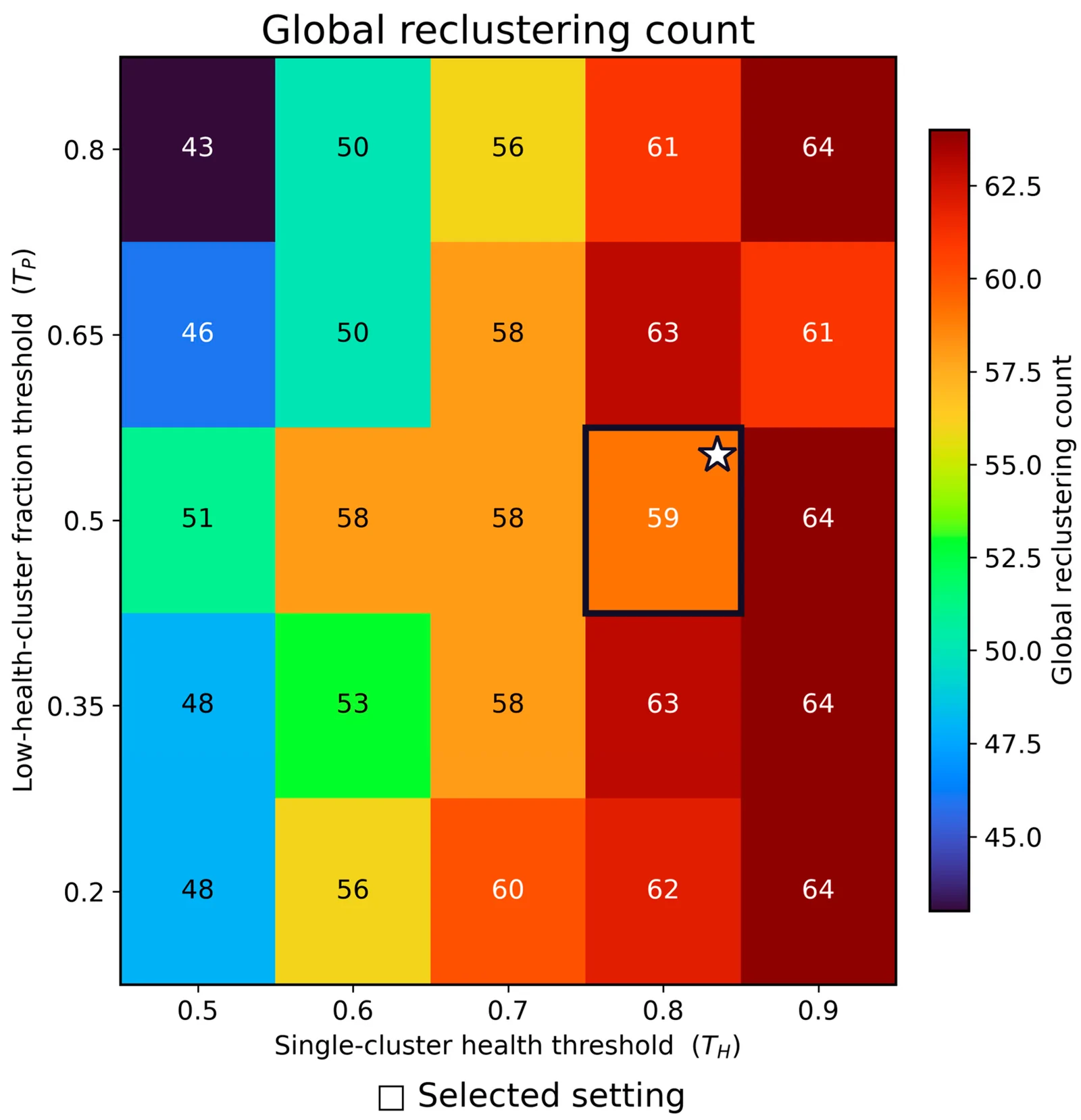
Global reclustering counts across maintenance thresholds. The star and outlined cell mark the selected setting, *T_H_* = 0.8 and *T_P_* = 0.5. Cell colors and numerical labels indicate the number of global reclustering events.

The combination with the lowest control energy, 0.2388 J, yields an FND of 892 rounds, showing that minimizing overhead does not necessarily extend the all-node survival period. The selected setting, $T_{H}=0.8$ and $T_{P}=0.5$, consumes 0.2475 J while achieving an FND of 940 rounds. It therefore improves early survival with control energy in the lower part of the tested range. Figure 9 reports global reclustering counts to clarify the source of control-energy changes.

The number of global reclustering events ranges from 43 to 64 and generally increases with $T_{H}$. A higher health threshold classifies clusters as unhealthy earlier, making it more likely that their proportion reaches $T_{P}$. The response to $T_{P}$ is not strictly monotonic, reflecting the interaction between local rotation, node deaths, and changes in cluster health after reconstruction.

The selected combination, $T_{H}=0.8$ and $T_{P}=0.5$, produces 59 global reclustering events. Although it does not minimize reconstruction frequency, it achieves an FND of 940 rounds and the highest HND among the tested settings with $T_{H}=0.8$. Its cumulative control energy is 0.2475 J, indicating that these lifetime results are achieved with comparatively low maintenance overhead within the tested range.

Considering lifetime and control overhead together, the thresholds are set to:

(34) $$T_{H}=0.8,\;T_{P}=0.5.$$

This combination gives precedence to the all-node survival period while retaining favorable midlife survival and control-energy performance. These thresholds are used in the subsequent validation, ablation, protocol-comparison, and base-station location experiments.

### 5.3. Visualization of UEAD Clustering and Multihop Routing

With the operating parameters selected, we examine how UEAD forms clusters and constructs collection routes. Visualizations of unequal clustering and EPPR–ACO planning on the same fixed node deployment illustrate the resulting structure and route feasibility, linking competition radii, cluster memberships, and forwarding paths to the mechanisms underlying the lifetime results.

#### 5.3.1. Unequal Clustering Process

**Figure 10:**
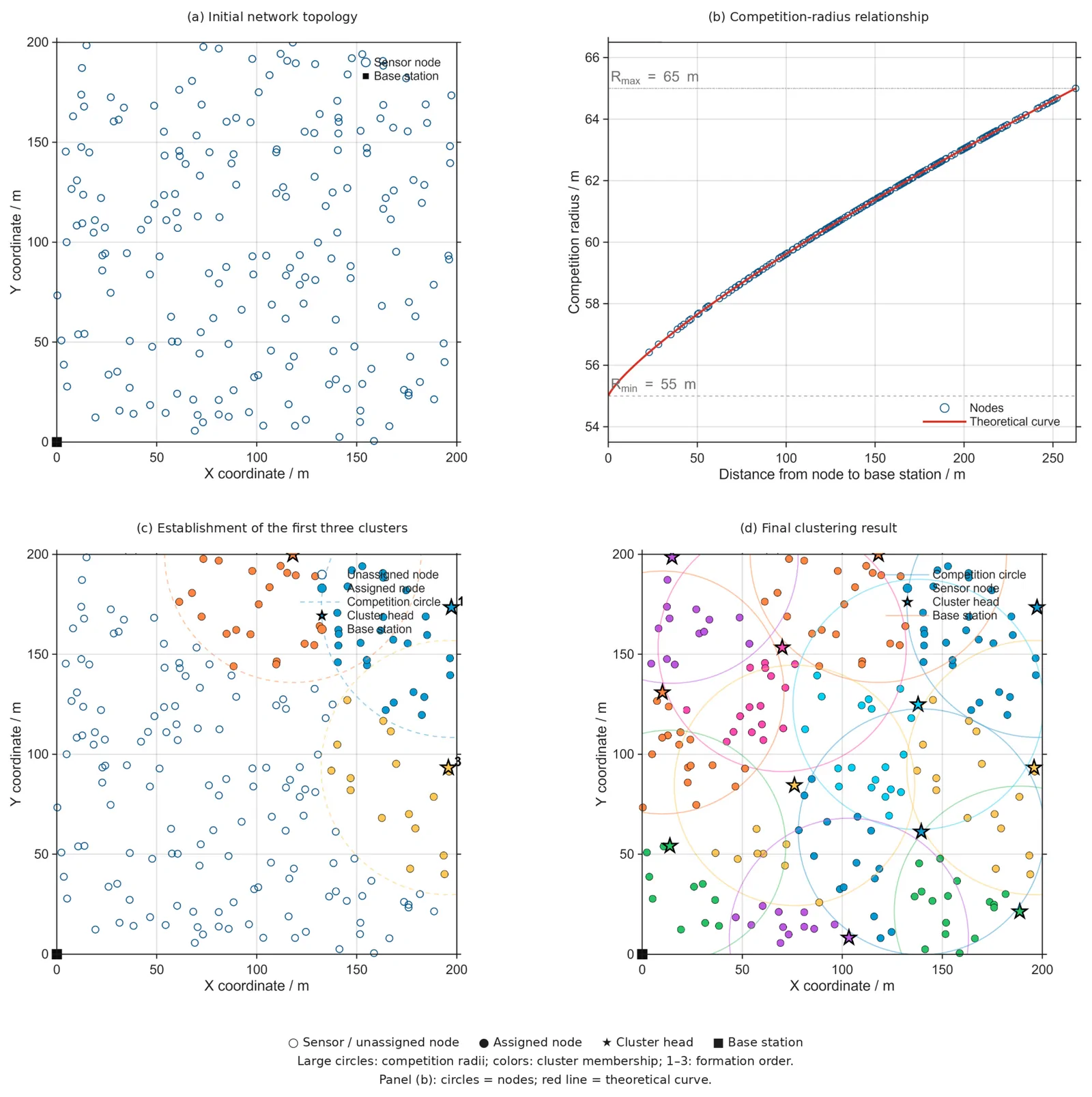
Unequal-cluster formation. In panels (**a**,**c**,**d**), dots denote sensor nodes and the square denotes the base station. In panels (**c**,**d**), stars denote cluster heads, colors distinguish cluster memberships, and large circles show competition radii. The numbers in panel (**c**) indicate cluster-formation order. Coverage circles extending beyond the deployment area are clipped at the plot boundary.

Figure 10 illustrates the clustering process for 200 randomly deployed nodes in a 200 m × 200 m area, with the base station at (0, 0). All four panels use the same node coordinates to show successive stages on a fixed deployment.

Figure 10a shows the initial node deployment before cluster formation, when all sensors have the same role. With the base station at the lower-left corner, node-to-base-station distances vary considerably across the area. A fixed competition radius does not account for these differences in collection distance. UEAD uses distance-dependent radii to regulate member-data reception loads and the spatial distribution of source heads. Intercluster forwarding is subsequently assigned to ordinary nodes during route planning.

Figure 10b shows how the competition radius varies with node-to-base-station distance. The radius increases with distance within the following bounds:

(35) $$R_{\min}\leq R_{i}\leq R_{\max},$$

where $R_{\min}=55$ m and $R_{\max}=65$ m. Nearby candidates have smaller competition radii, while more distant candidates cover larger areas.

Figure 10c illustrates the sequential formation of the first three clusters. Once a head is selected, unassigned nodes within its competition radius join the cluster and are removed from further consideration. The procedure continues until all nodes are assigned. Distance-dependent radii and deterministic candidate ordering govern the head distribution, while greedy coverage prevents assigned nodes from participating in subsequent head selection.

Figure 10d shows the resulting clusters. Colors distinguish cluster memberships, stars mark cluster heads, and circles indicate their competition radii. The coverage areas are spatially unequal, with smaller radii near the base station and larger radii farther away. The number of members in each cluster also depends on local node density and the order of formation.

The circles represent coverage during cluster formation. After local head rotation, memberships are retained, so the original circles need not describe coverage around the replacement heads. Global reclustering establishes new heads and memberships. Figure 10 therefore depicts one clustering configuration within an evolving network structure.

#### 5.3.2. EPPR–ACO Intercluster Multihop Planning

**Figure 11:**
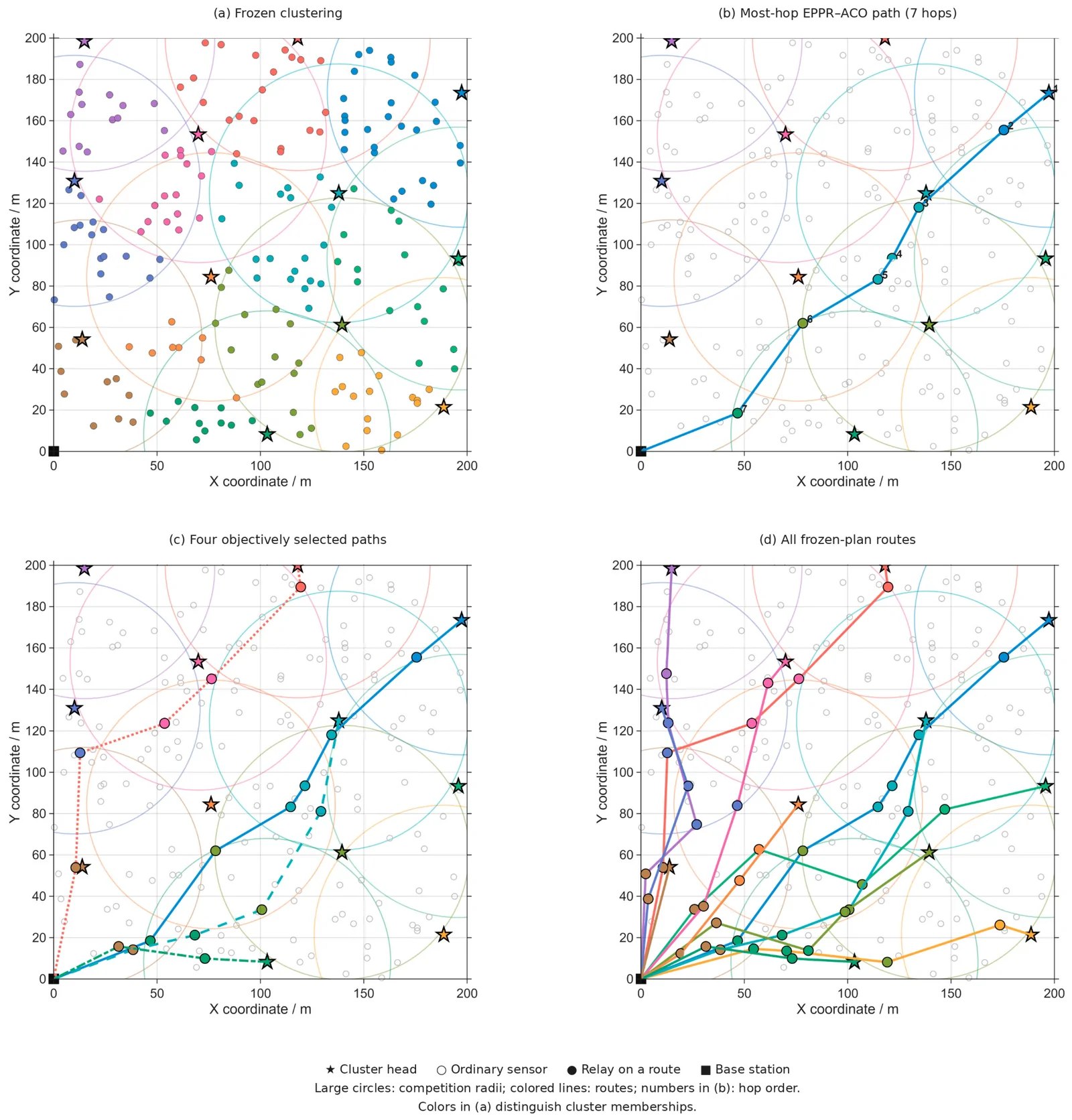
EPPR–ACO route construction. Stars denote cluster heads, small circles denote sensor nodes, and the square denotes the base station. Large circles show competition radii. Colors distinguish cluster memberships in (**a**) and routes in (**c**,**d**); highlighted circles on a route denote relay nodes. In (**b**), the numbers label successive hops along the highlighted route. Overlapping routes and coverage circles reflect their spatial geometry.

After cluster formation, each head delivers its aggregated data to the base station. For distant heads, direct transmission can incur substantial amplifier energy costs. UEAD uses EPPR-initialized ACO to construct multihop routes through ordinary relay nodes, as illustrated in Figure 11.

Figure 11a shows the cluster configuration before route planning. Colors distinguish cluster memberships, stars mark the heads, and circles indicate competition radii. Heads and memberships remain fixed while intercluster routes are constructed.

Figure 11b highlights the route with the largest hop count in this configuration. A distant head reaches the base station in seven hops through ordinary relay nodes. Each link satisfies the communication-range constraint, and no node is revisited. This route illustrates how a distant source can reach the base station without a single long-range transmission. Other cluster heads are excluded from relaying, keeping their duties limited to receiving, aggregating, and transmitting their own cluster data.

Figure 11c presents four representative routes covering short, intermediate, and long source-to-base-station distances, together with the maximum-hop route. Hop counts vary with source location, available relays, and link costs. Nearby heads can use direct or short multihop routes, whereas distant heads may require additional relays to satisfy the communication-range constraint.

Figure 11d shows the complete route set for the illustrated topology, with every source cluster head connected to the base station. The routes need not be geometric shortest paths because construction combines radio-link communication costs, EPPR-based pheromone initialization, and feasibility constraints.

Routes are planned from distant to nearby source heads under the strict assignment rules in Section 4.3.4. Each ordinary relay is reserved for at most one active source route, and its reservation is released when that route is invalidated. This prevents simultaneous relay sharing without guaranteeing balanced cumulative relay use over time.

### 5.4. Validation of EPPR Guidance

We compare the complete uniformly initialized ACO and EPPR–ACO implementations on paired tasks with the same node topology, source heads, and available relay nodes. The equal-iteration experiments use matched iteration counts and routing feasibility conditions, but the original implementations also differ in search rules beyond pheromone initialization; their differences therefore cannot be attributed to EPPR initialization alone. In a separate diagnostic, switching between uniform and EPPR initialization within each fixed search core did not reduce mean energy in either core on the tested tasks. The equal-total-time comparison in Section 5.4.5 evaluates the complete implementations and includes EPPR preprocessing.

#### 5.4.1. Path Structures Under an Equal Iteration Budget

**Figure 12:**
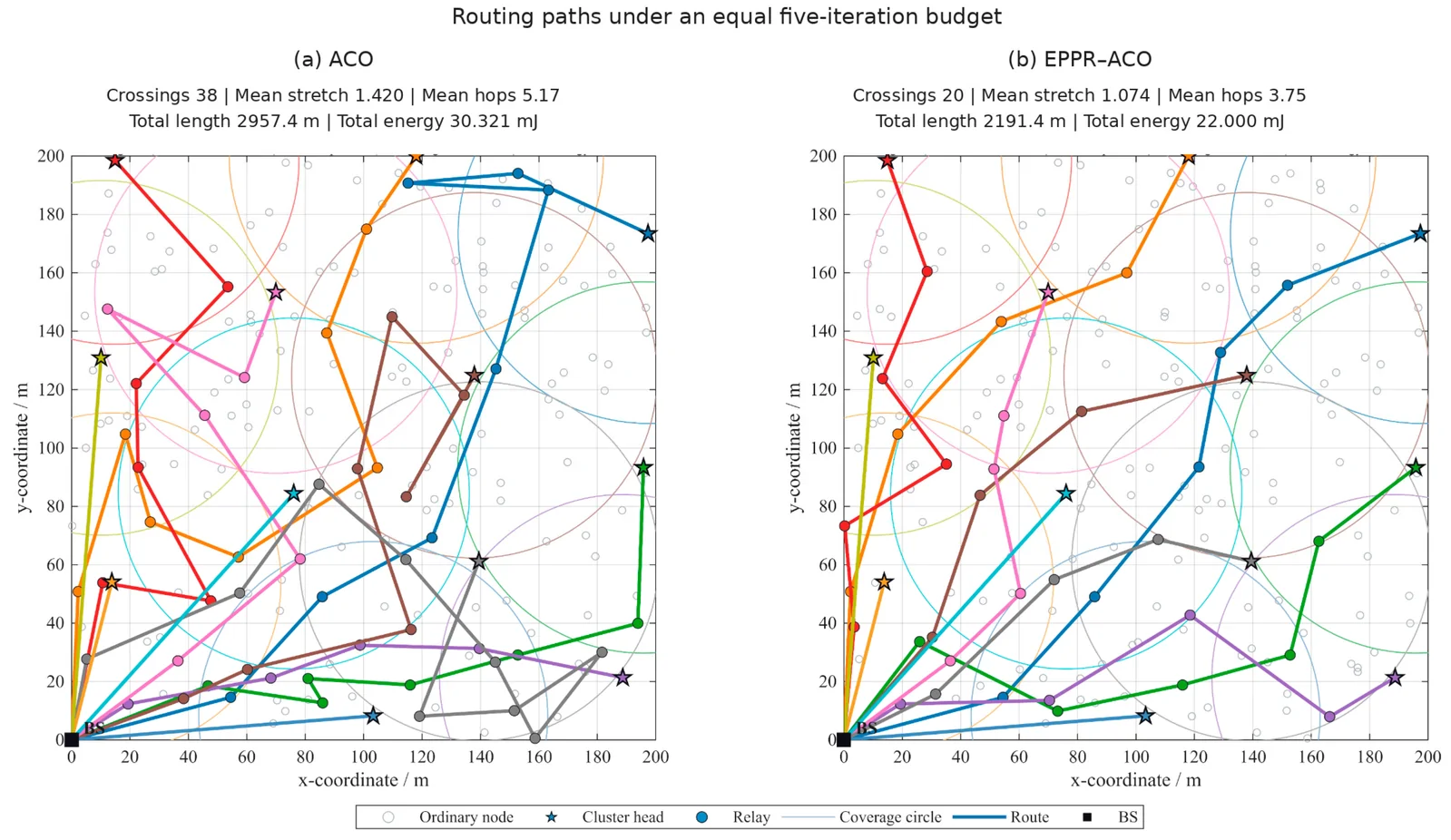
ACO and EPPR–ACO routes after five iterations. Stars denote cluster heads, hollow circles denote ordinary nodes, filled circles on routes denote relays, and the square denotes the base station. Thin circles show coverage regions; thick colored lines distinguish routes. Geometric crossings of routes and coverage circles are retained.

Figure 12 compares the complete intercluster route sets obtained by the two methods after five ACO iterations. Both use the same cluster configuration and candidate relays, allowing route differences to be assessed under matched conditions.

Figure 12a shows the routes obtained with uniformly initialized ACO. Early next-hop choices depend on local heuristics and stochastic exploration, without a differentiated pheromone prior. After five iterations, several routes contain detours and geometric crossings, and some distant heads reach the base station through additional relays.

Figure 12b shows the routes generated by EPPR–ACO. EPPR supplies a nonuniform pheromone prior derived from base-station personalization and link communication costs before ACO constructs the routes. Several displayed routes are less circuitous and shorter than their counterparts in Figure 12a, illustrating the route-planning performance of the complete EPPR–ACO implementation.

#### 5.4.2. Convergence Analysis

**Figure 13:**
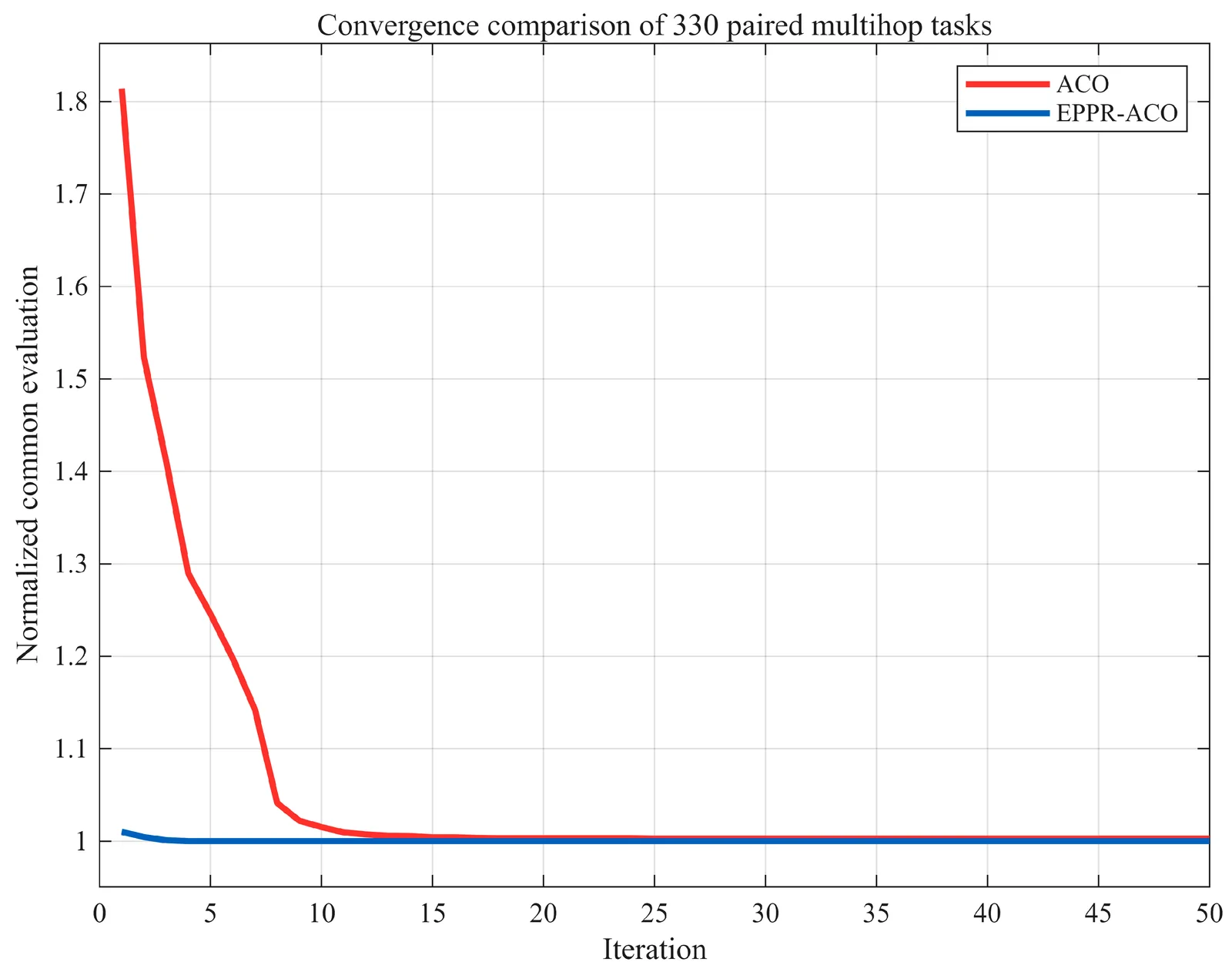
Normalized convergence of ACO and EPPR–ACO.

The common evaluation score combines communication energy, path stretch weighted by $10^{-4}$, and the squared number of ordinary relays weighted by $10^{-5}$. The 330 paired observations comprise 11 multihop routing contexts, each evaluated in 30 paired repetitions. For each observation, both methods’ scores at every iteration are divided by the lowest finite score across both methods and all recorded iterations; Figure 13 reports the median normalized score at each iteration. Thus, normalization uses a shared task-specific reference rather than either method’s final score. EPPR–ACO stabilizes after approximately three iterations, compared with approximately 15 iterations for ACO, highlighting its faster convergence under a limited iteration budget. Section 5.4.6 reports absolute communication energy: EPPR–ACO is lower at five iterations, whereas ACO is marginally lower at 20, 50, and 100 iterations. Figure 13 excludes EPPR preprocessing; the equal-time comparison in Section 5.4.5 includes it.

#### 5.4.3. Per-Cluster Route Energy

**Figure 14:**
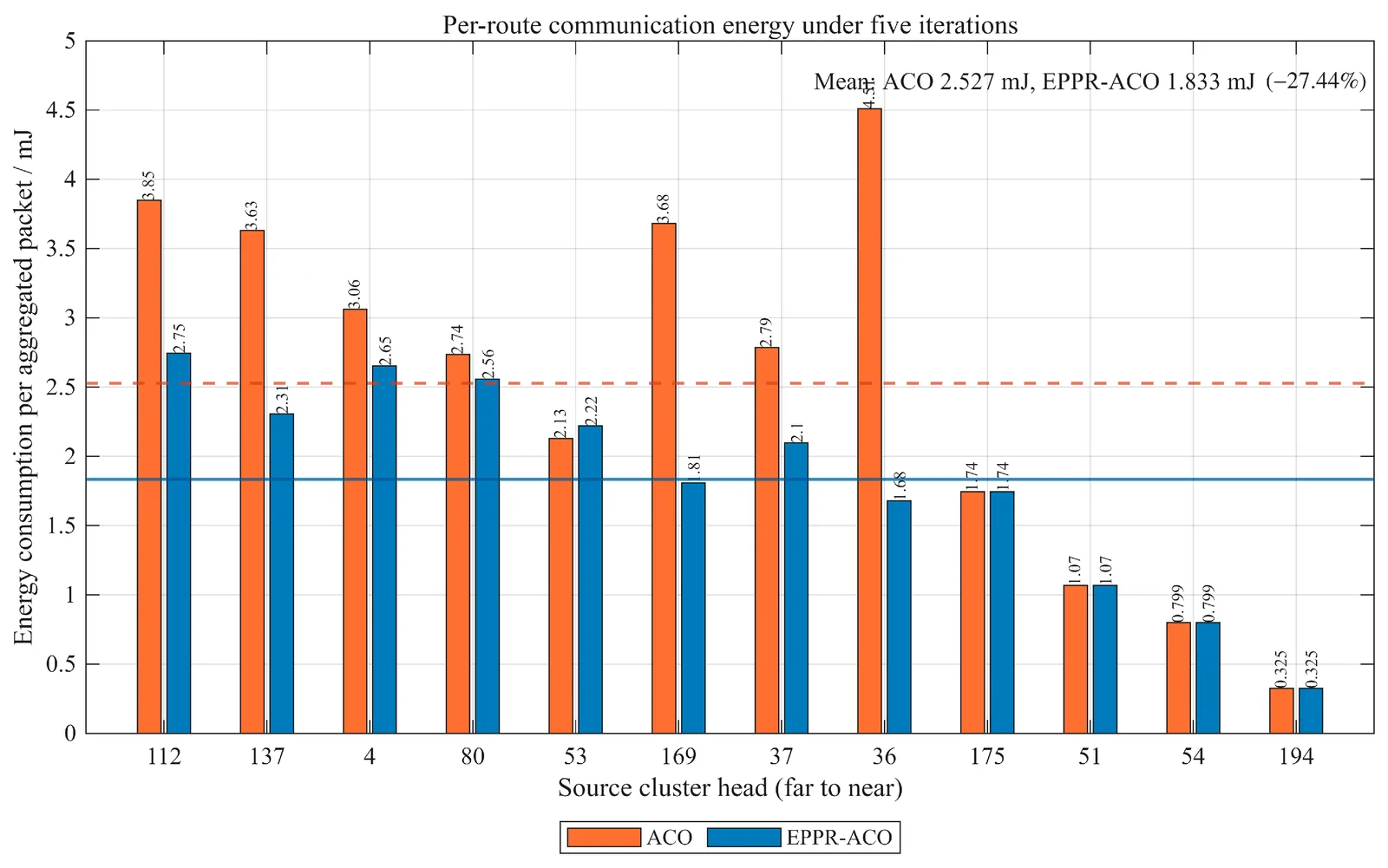
Per-cluster route energy after five iterations.

To examine whether the total energy reduction is concentrated in a few costly routes, Figure 14 compares the communication energy required to deliver one aggregated packet from each of the 12 source heads to the base station. Heads are ordered by decreasing distance to the base station, and the labels above the bars report the calculated energy for each route.

Mean route energy decreases from 2.527 mJ with uniformly initialized ACO to 1.833 mJ with EPPR–ACO, which is a reduction of 27.44%. Because both methods serve the same 12 source heads, the relative reduction is identical for mean and total route energy.

The complete EPPR–ACO implementation has lower route energy for heads 112, 137, 169, and 36 in this route-set comparison. This source-specific pattern is descriptive and cannot be attributed to pheromone initialization alone.

Heads 175, 51, 54, and 194 have identical or similar route costs under the two methods, whereas head 53 has slightly higher energy consumption under EPPR–ACO. The aggregate energy reduction therefore does not imply an advantage for every source.

#### 5.4.4. Energy Components and Path Complexity

**Figure 15:**
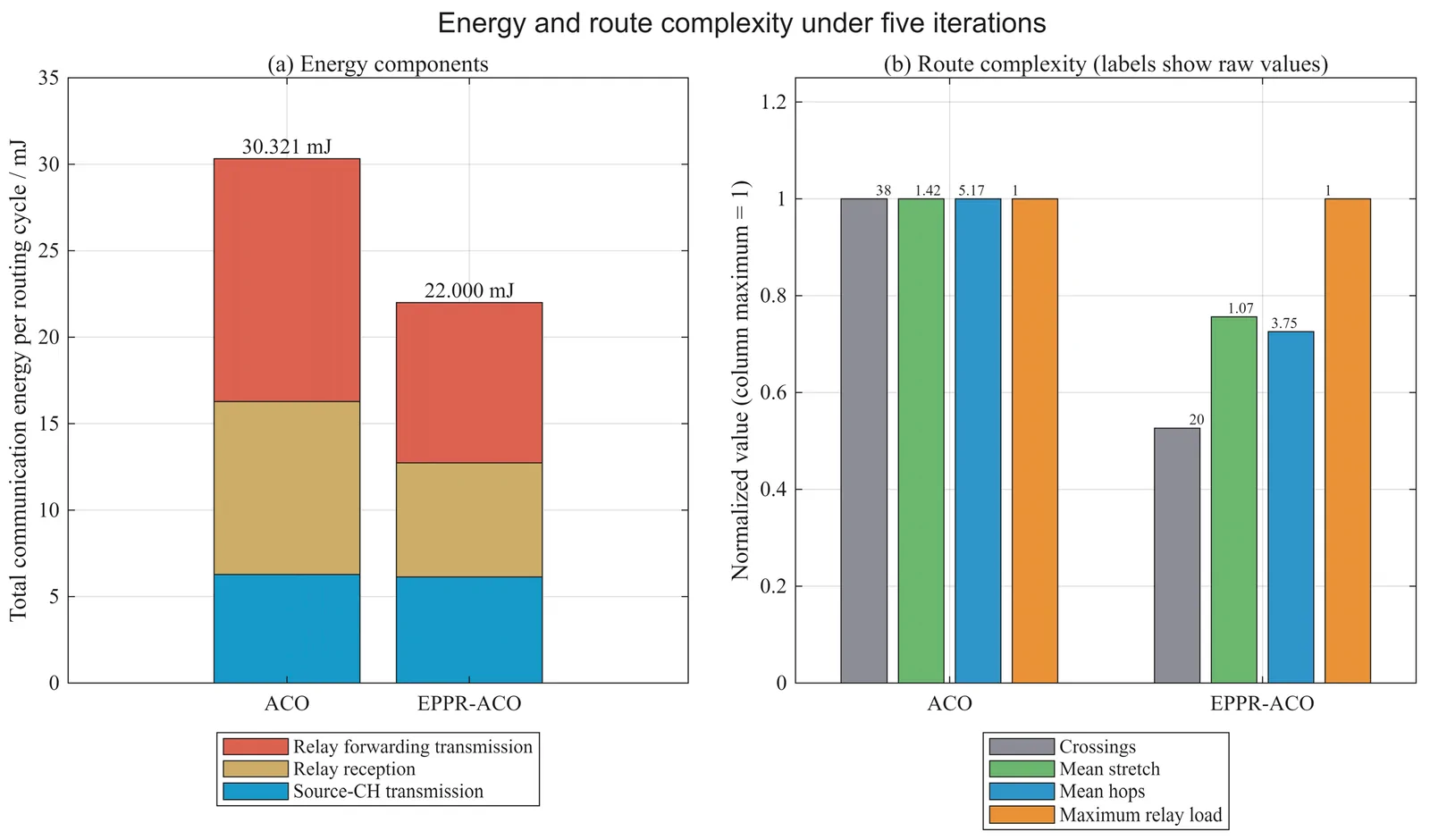
Route-energy components and path complexity.

Figure 15 compares communication-energy components and route structure. Energy consumption is separated into source-head transmission, relay reception, and relay retransmission. Route structure is characterized by crossing count, mean path stretch, mean hop count, and the maximum number of source routes sharing a relay.

Figure 15a shows a relatively small difference in source-head transmission energy, with larger reductions in relay reception and retransmission energy under EPPR–ACO. This pattern is consistent with the decrease in mean hop count from 5.17 to 3.75. Fewer relay stages reduce the number of reception and retransmission operations, contributing to the lower total communication cost.

Figure 15b presents normalized structural metrics, with their original values labeled above the bars. EPPR–ACO produces fewer crossings, lower mean path stretch, and fewer hops. The maximum number of routes sharing a relay is identical for both methods because both enforce exclusive relay use. This equality reflects a shared constraint rather than an independent improvement in load balance. It describes simultaneous route occupancy, not the cumulative forwarding burden on individual nodes over time.

Table 2 compares the route metrics.

On the tested topology, EPPR–ACO reduces the number of route crossings from 38 to 20, a decrease of 47.37%. This indicates fewer geometric intersections, although crossing count alone does not establish an energy benefit. Energy savings are assessed through route length, hop count, and the associated transmission and reception costs.

Mean path stretch decreases from 1.420 to 1.074, indicating that routes more closely approach the direct source-to-base-station distances. Mean hop count falls from 5.17 to 3.75, while total path length decreases from 2957.4 to 2191.4 m. The complete EPPR–ACO implementation therefore produces routes with fewer detours and relay stages under the tested iteration budget.

These route changes are accompanied by a reduction in total communication energy from 30.321 to 22.000 mJ, or 27.44%. With source heads and packet lengths held constant, the savings reflect shorter routes, fewer relay stages, and the resulting changes in transmission and reception costs.

Figure 12, Figure 13, Figure 14 and Figure 15 characterize the two complete implementations under an equal iteration budget. Section 5.4.5 evaluates performance under matched total planning time, including EPPR preprocessing.

#### 5.4.5. Route Quality Under Equal Total Planning-Time Budgets

To account for EPPR preprocessing overhead, the total-time budget starts before EPPR preprocessing and pheromone setup and includes route search; file loading and plotting are excluded. We tested budgets of 62, 149, 300, and 541 ms on one frozen topology, using 330 paired tasks from 11 source-head contexts with 30 stochastic repetitions per context. Ant-search calls are nonpreemptive and may slightly exceed a budget. Success counts use all 330 tasks; mean route energy is calculated over tasks for which both methods succeeded. Table 3 reports the success counts and mean route communication energy for these budgets.

At 62 and 149 ms, EPPR–ACO has lower mean route energy on the jointly successful tasks. At 300 and 541 ms, the methods are close and ACO is slightly lower. Thus, EPPR–ACO does not outperform ACO at every tested budget. At 541 ms, the measured mean total planning times were 541.54 ms for EPPR–ACO and 541.59 ms for ACO; EPPR preprocessing averaged 4.72 ms per source. These machine-specific measurements include EPPR preprocessing in the end-to-end comparison. The comparison evaluates the complete implementations, whose search rules differ beyond pheromone initialization.

These per-task planning times do not represent the duration of network-wide route reconstruction.

#### 5.4.6. Performance Across ACO Iteration Budgets

To test whether the five-iteration result depended on a short search budget, we compared the complete uniformly initialized ACO and EPPR–ACO implementations at 5, 20, 50, and 100 iterations on the same 330 paired routing tasks (11 source-head contexts × 30 repetitions) from one frozen topology. Table 4 reports mean ± sample standard deviation for route communication energy, path length, and hop count, together with successful-path counts. Both methods completed all 330 tasks at every budget. The standard deviations describe repeated task outcomes within this topology and do not represent variation across independent deployments.

**Figure 16:**
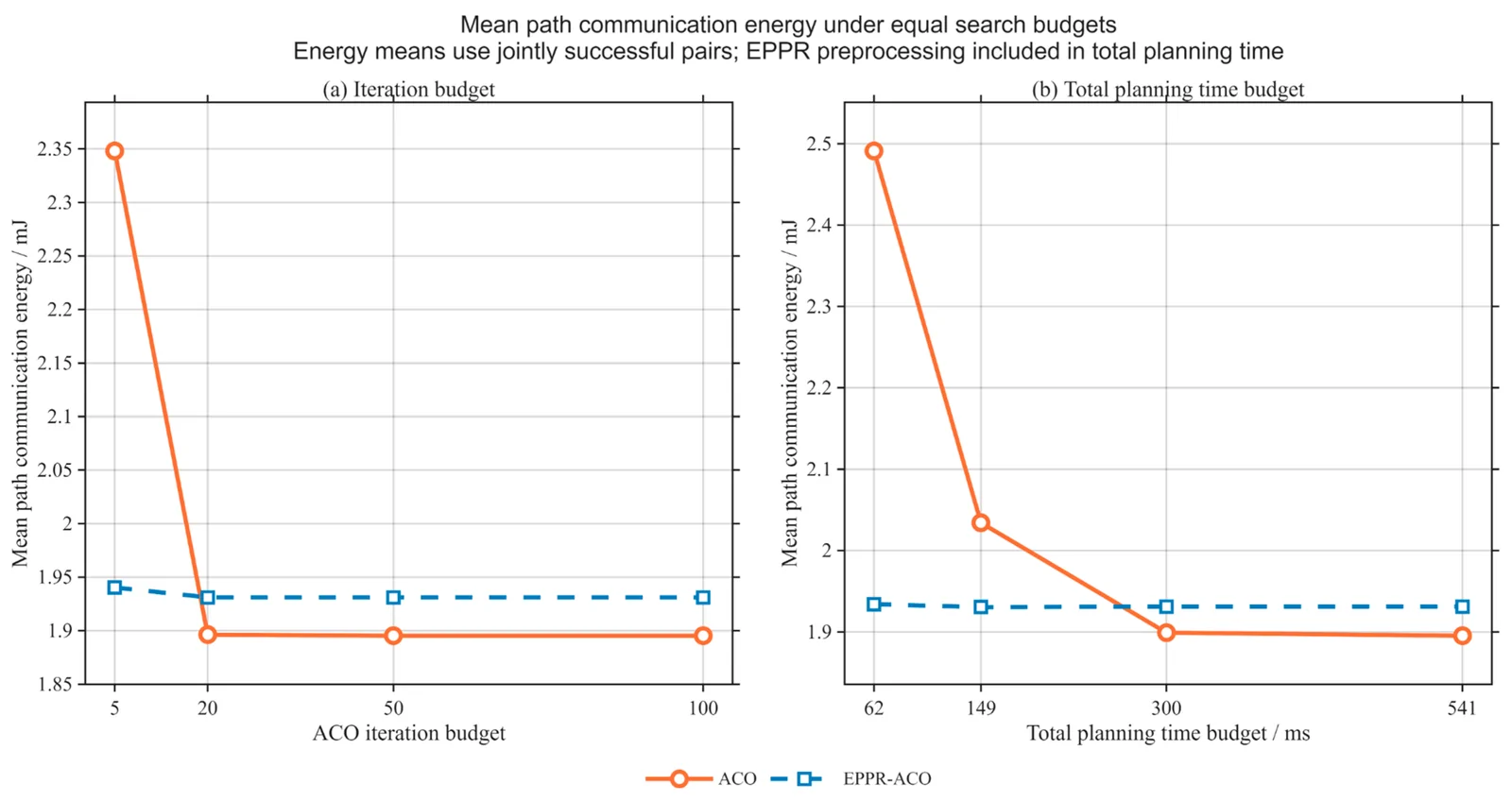
Route energy under equal iteration and time budgets.

At five iterations, EPPR–ACO reduced mean route energy from 2.348 to 1.940 mJ. At 20, 50, and 100 iterations, uniformly initialized ACO achieved slightly lower mean energy (1.896, 1.895, and 1.895 mJ, respectively) than EPPR–ACO (1.931 mJ). The energy and hop-count results changed little after 20 iterations. EPPR–ACO nevertheless retained shorter mean paths at 20–100 iterations (189.2 m versus about 198.7 m), whereas ACO used slightly fewer mean hops (4.26–4.27 versus 4.48). Thus, the five-iteration energy result does not extend uniformly to larger iteration budgets: on these tasks, ordinary ACO matched and slightly surpassed EPPR–ACO in path communication energy at the longer budgets, while EPPR–ACO produced shorter geometric paths. Because the two implementations also differ in search rules, this comparison evaluates their complete procedures and does not isolate an EPPR-initialization effect. End-to-end planning-time results, including EPPR preprocessing, are reported in Section 5.4.5. Figure 16 summarizes the energy results under both budget types.

### 5.5. Module Ablation Experiments

Route-quality experiments compare the complete ACO and EPPR–ACO implementations under equal iteration and total planning-time budgets, while network lifetime also depends on cluster formation and maintenance. To assess these mechanisms, UC-ACO-D, UC-EA-F, UEAD-Uniform, and the complete UEAD protocol are compared using the same node topology, energy model, and evaluation criteria.

Table 5 summarizes the four variants.

The variants follow the configurations in Section 5.1.4: UC-ACO-D changes pheromone initialization, UC-EA-F uses scheduled global reclustering every 50 rounds, and UEAD-Uniform uses a fixed 60 m competition radius. Table 5 identifies the modules retained in each variant.

#### 5.5.1. Surviving-Node Evolution Across Variants

**Figure 17:**
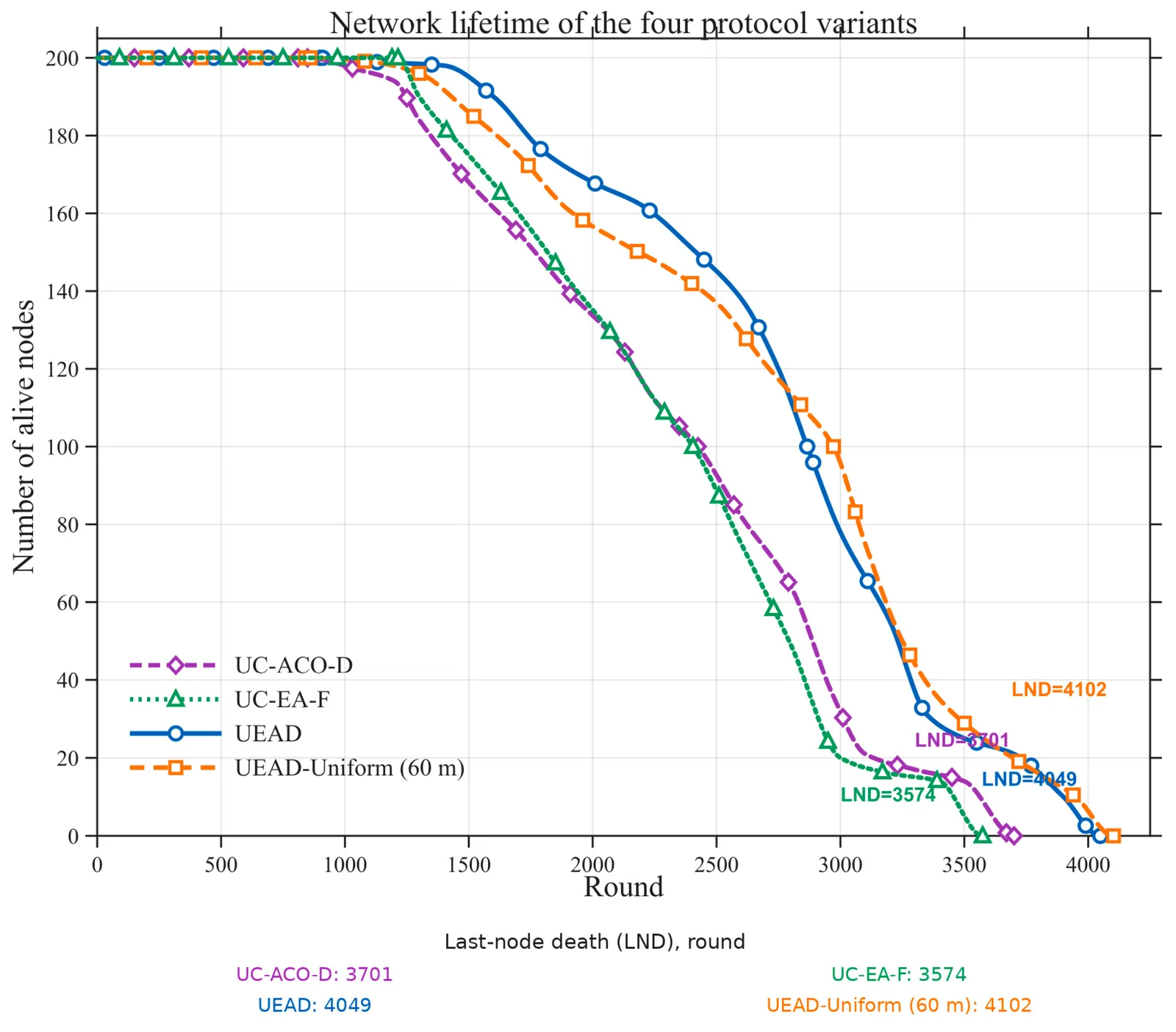
Surviving nodes across protocol variants.

Figure 17 shows how the number of surviving nodes changes over successive rounds for each protocol variant. Unlike individual lifetime metrics, the complete survival curves reveal differences in the onset of node death and in the subsequent patterns of network decline.

Once node deaths begin, the four variants exhibit distinct survival patterns. The curves for UC-ACO-D and UC-EA-F remain relatively close and intersect several times during the middle stage. UEAD and UEAD-Uniform retain more surviving nodes through much of the simulation, consistent with the combined contribution of EPPR initialization and health-driven maintenance to mid- and late-stage survival.

UC-ACO-D uses uniformly initialized ACO while retaining unequal clustering and dynamic maintenance. Without the EPPR prior, route construction under a limited iteration budget depends more heavily on local heuristics and stochastic exploration. The resulting routes may contain additional relays or detours, whose communication costs accumulate over repeated rounds. UC-ACO-D reaches LND at round 3701, but its survival curve remains below that of UEAD through much of the middle and late stages.

UC-EA-F retains EPPR–ACO routing but replaces health-driven maintenance with full-network reclustering every 50 rounds. UEAD checks cluster health on the same 50-round cadence but performs local rotation or global reclustering according to the measured thresholds. The fixed schedule therefore rebuilds clusters and routes even when the health criteria would not request a global update; it also periodically reassigns cluster-head roles, which may redistribute early communication loads. This provides a plausible explanation for the later FND of UC-EA-F in this run (1214 rounds, compared with 899 rounds for UEAD), but the lifetime results do not quantify wall-clock computational overhead. UC-EA-F preserves nearly all nodes until approximately round 1200, its decline becomes more pronounced after approximately round 2500, and its final node dies at round 3574.

UEAD maintains a larger surviving population than UEAD-Uniform over a substantial part of the early and middle stages. This difference is consistent with distance-adaptive coverage delaying some early failures by regulating cluster memberships and the distribution of source heads. The curves intersect near round 2900, after which UEAD-Uniform declines more slowly. Consequently, UEAD reaches LND at round 4049, whereas UEAD-Uniform reaches LND at round 4102, a difference of 53 rounds, or approximately 1.31%. Unequal clustering therefore improves early survival under the tested conditions but does not produce the longest tail lifetime.

#### 5.5.2. Lifetime Metrics of the Protocol Variants

**Figure 18:**
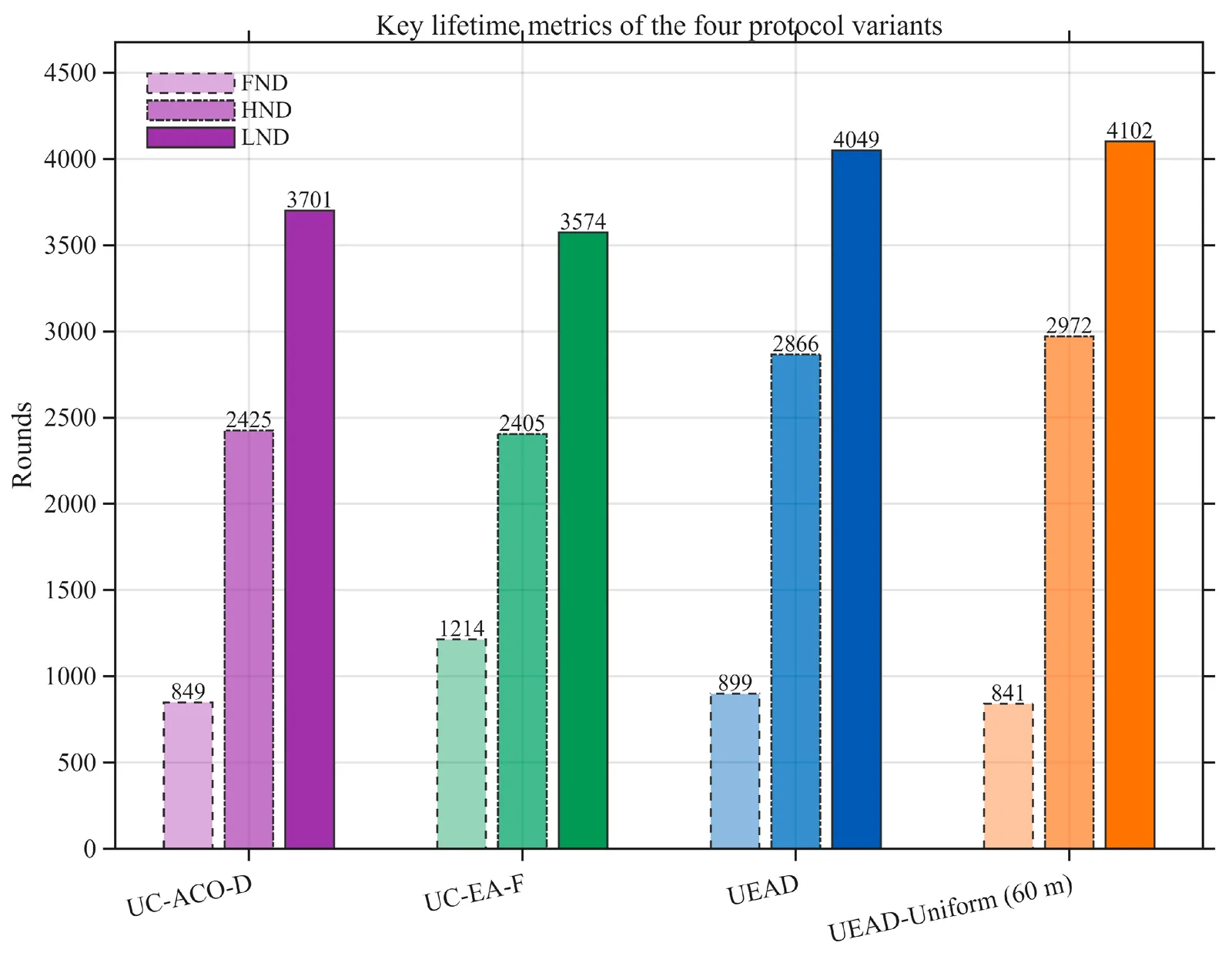
Lifetime metrics of the protocol variants. Purple, green, blue, and orange identify UC-ACO-D, UC-EA-F, UEAD, and UEAD-Uniform (60 m), respectively. Within each group, the bars from left to right represent FND, HND, and LND; the values above the bars give the corresponding rounds.

Figure 18 compares FND, HND, and LND across the four protocol variants, with the corresponding values reported in Table 6.

The ablation results show that EPPR initialization, health-driven maintenance, and unequal clustering affect different stages of network survival.

Replacing EPPR initialization with uniform pheromones reduces all three lifetime metrics. Relative to UC-ACO-D, UEAD improves FND, HND, and LND by 5.89%, 18.19%, and 9.40%, respectively. The largest relative gain occurs in HND, indicating that the route-level savings obtained under a limited ACO iteration budget accumulate most clearly during the middle stage of network operation.

The numerical comparison confirms a stage-dependent trade-off. UC-EA-F reaches FND at round 1214, 315 rounds later than UEAD at round 899. UEAD, however, reaches HND and LND at rounds 2866 and 4049, compared with 2405 and 3574 for UC-EA-F. Thus, the fixed schedule is associated with later first-node death in this experiment, whereas health-driven maintenance is associated with longer middle- and late-stage survival. These results describe the tested setting and do not establish that fixed-period maintenance is generally superior for FND.

UEAD-Uniform isolates the effect of unequal clustering while retaining EPPR–ACO and health-driven maintenance. Its fixed 60 m competition radius produces an FND of 841 rounds, below the 899 rounds achieved by UEAD. However, its HND and LND reach 2972 and 4102 rounds, exceeding UEAD by 3.70% and 1.31%, respectively. Distance-adaptive unequal clustering therefore delays the first failure under the tested conditions but does not improve HND or LND relative to the uniform-clustering variant.

**Figure 19:**
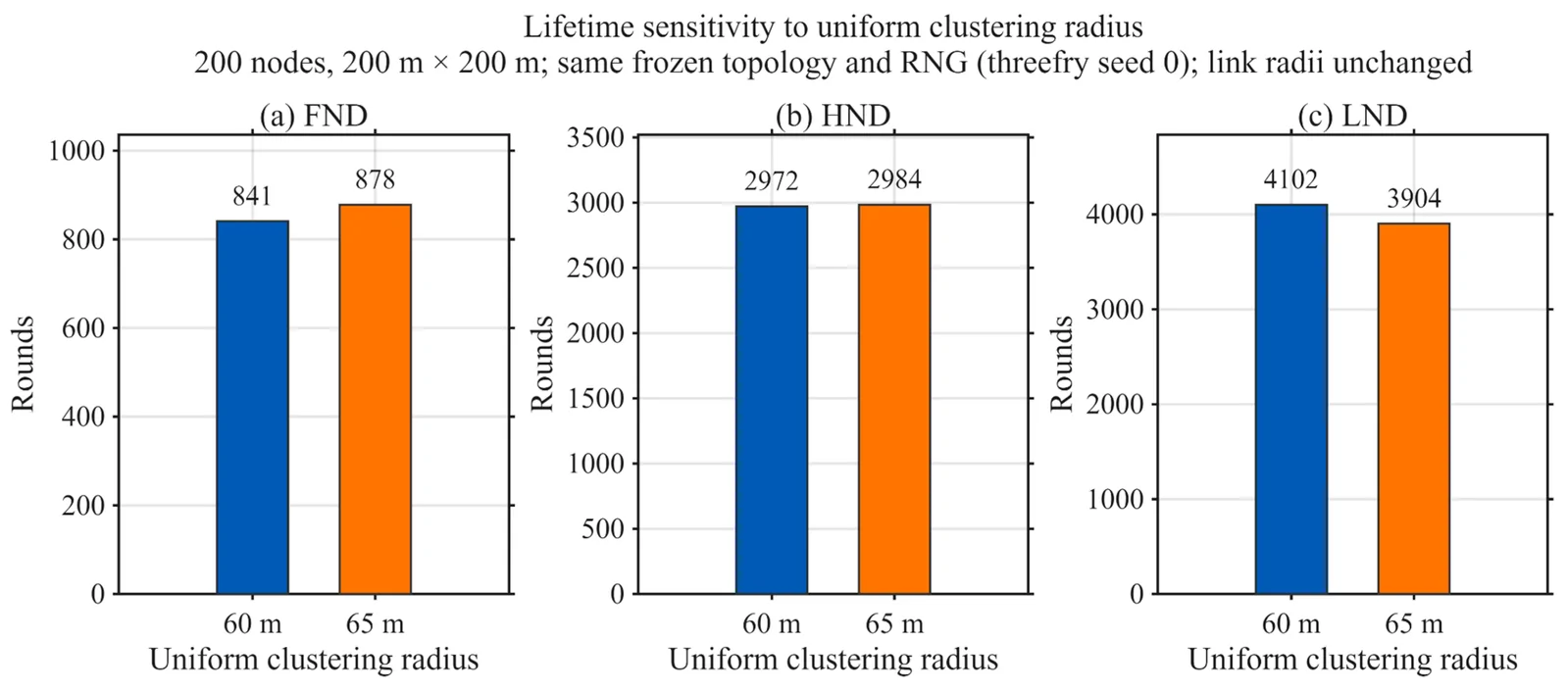
Lifetime sensitivity to the uniform clustering radius.

We additionally examined the uniform competition radius at 55, 60, and 65 m using the same frozen topology and random state. At 60 m, FND, HND, and LND were 841, 2972, and 4102 rounds; at 65 m, they were 878, 2984, and 3904 rounds. The 55 m run failed during route initialization at cluster head 123, so no lifetime values are available for that setting. The 65 m result improves FND and HND relative to 60 m but reduces LND, showing that the observed effect is not monotonic across lifetime metrics. This matched sensitivity check extends the tested range to 65 m, but its single-topology, single-state design does not establish radius-independent performance. The available 60 m and 65 m results are shown in Figure 19.

Overall, the three mechanisms contribute at different stages rather than improving every lifetime metric simultaneously. EPPR initialization benefits FND, HND, and LND relative to uniform pheromone initialization, while health-driven maintenance exchanges part of the early-stage advantage of fixed-period reclustering for stronger middle- and late-stage survival. Unequal clustering primarily improves FND in the present ablation. UEAD consequently provides a tradeoff between early protection and sustained operation rather than a uniform optimum across all metrics.

### 5.6. Overall Comparison with Existing Protocols

Following the module-level evaluation, the complete UEAD protocol is compared with standard LEACH and DUCISCA under matched simulation conditions. Each protocol is run independently on the same node deployments using identical initial energy, radio parameters, packet lengths, and node-death definitions. This design limits differences caused by random topology and allows the observed results to be attributed more directly to protocol behavior.

Standard LEACH represents probabilistic cluster-head election with direct head-to-base-station transmission, whereas DUCISCA provides a comparison with an improved clustering protocol. UEAD combines distance-adaptive unequal clustering, EPPR-initialized ACO routing, and dual-threshold maintenance. Performance is evaluated using surviving-node trajectories, FND, HND, LND, and total residual energy.

#### 5.6.1. Surviving-Node Counts

**Figure 20:**
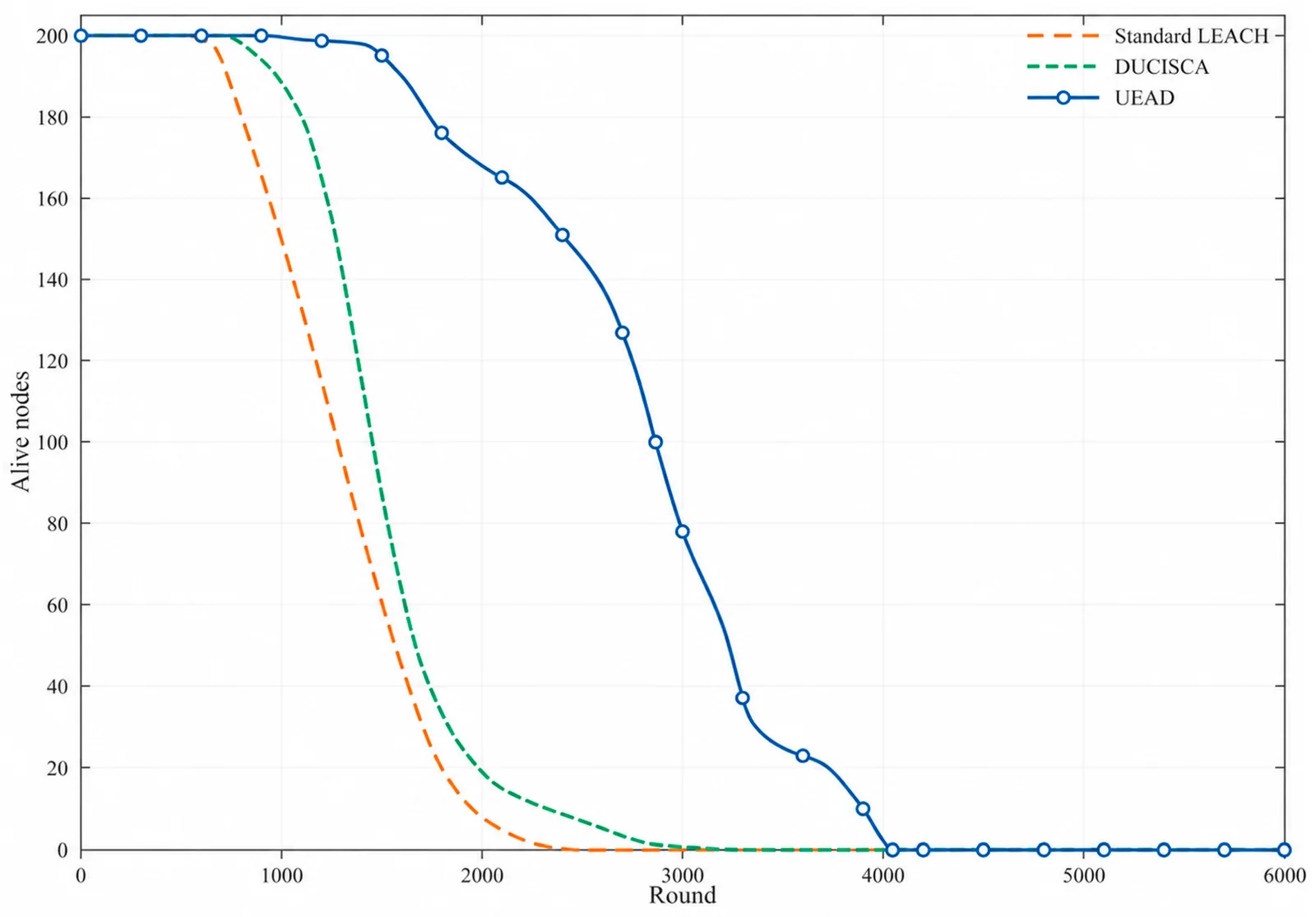
Surviving nodes under the three protocols.

Figure 20 shows the number of surviving nodes over successive rounds. All three protocols begin with 200 nodes but differ in the onset of node death and the subsequent rate of network decline.

Standard LEACH exhibits the earliest node deaths and the most rapid subsequent decline. Its probabilistic rotation does not explicitly coordinate cluster-head locations or cluster sizes with distance to the base station. With the base station at a corner, distant heads incur high direct-transmission costs, while uneven cluster formation can impose larger reception loads on some heads. These effects produce early energy disparities and a relatively short all-node survival period.

The DUCISCA survival curve generally lies to the right of that of standard LEACH. Its clustering and routing mechanisms delay the onset of node death and preserve more nodes during the early stage. The curve nevertheless declines more rapidly during the middle stage than that of UEAD, indicating a shorter period of sustained network-wide survival.

UEAD shifts the survival curve farther to the right. All 200 nodes remain alive until approximately round 940, after which the surviving-node count declines more slowly than for either baseline. UEAD therefore retains a larger active-node population through much of the middle and late stages.

UEAD reaches HND at approximately round 2920 and LND near round 4000. Its advantage is observed in the onset, midpoint, and completion of node loss. Unequal clustering regulates member loads and the distribution of distant source heads, EPPR–ACO reduces route detours and relay operations, and health-driven maintenance updates degraded structures during operation. The survival curve reflects the cumulative effect of these mechanisms on different components of network energy consumption.

#### 5.6.2. Lifetime Metrics of the Compared Protocols

**Figure 21:**
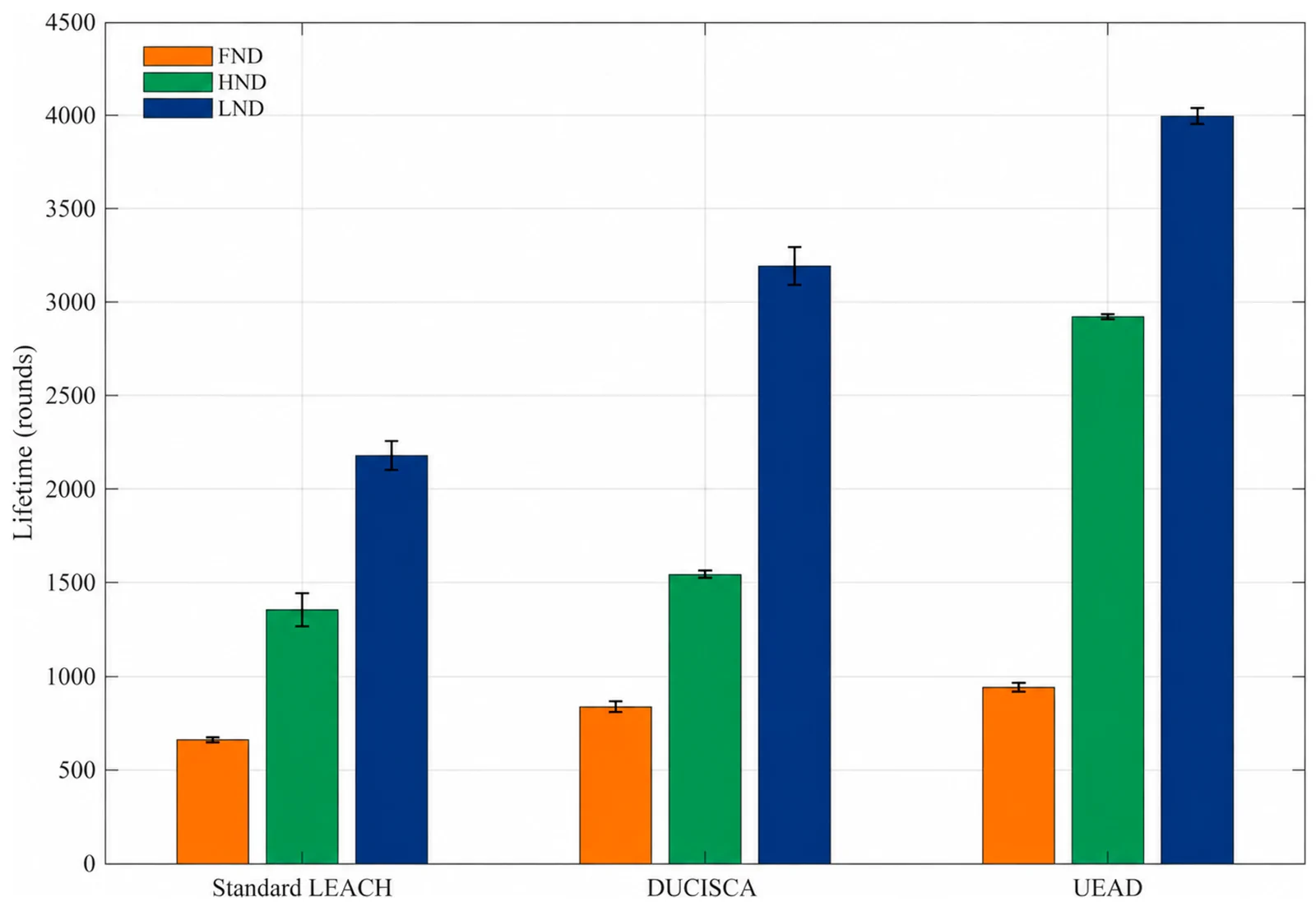
Lifetime metrics of the three protocols.

Figure 21 compares the FND, HND, and LND results of standard LEACH, DUCISCA, and UEAD across matched network deployments. The bars represent the mean values, while the error bars show the variation among deployments.

UEAD provides the longest all-node survival period among the three protocols. Standard LEACH experiences early node deaths associated with probabilistic cluster-head placement and long-distance direct transmission. DUCISCA extends FND relative to standard LEACH but remains below UEAD. This result is consistent with UEAD’s coordination of cluster memberships, intercluster route costs, and health-driven maintenance reducing the early depletion of heavily loaded nodes.

The difference between UEAD and the baselines becomes more pronounced at HND. UEAD reaches HND at approximately round 2920, indicating that its advantage persists as node deaths accumulate. This result is consistent with lower route costs conserving energy over repeated transmissions, while local rotation and global reclustering respond to emerging degradation. UEAD consequently retains a larger active-node population during the middle stage of operation.

UEAD reaches LND at approximately round 4000, later than standard LEACH and DUCISCA. Standard LEACH declines rapidly after the onset of node death, producing comparatively short stable and tail periods. DUCISCA delays node loss relative to standard LEACH but does not maintain the same late-stage survival as UEAD under the common simulation conditions.

Taken together, the FND, HND, and LND results show that UEAD delays the onset, midpoint, and completion of node loss. Its lifetime advantage is therefore not obtained by sacrificing the all-node stable period to preserve only a small number of late-surviving nodes. Variation across the matched deployments is reported by the error bars in Figure 21.

#### 5.6.3. Residual Energy of the Compared Protocols

**Figure 22:**
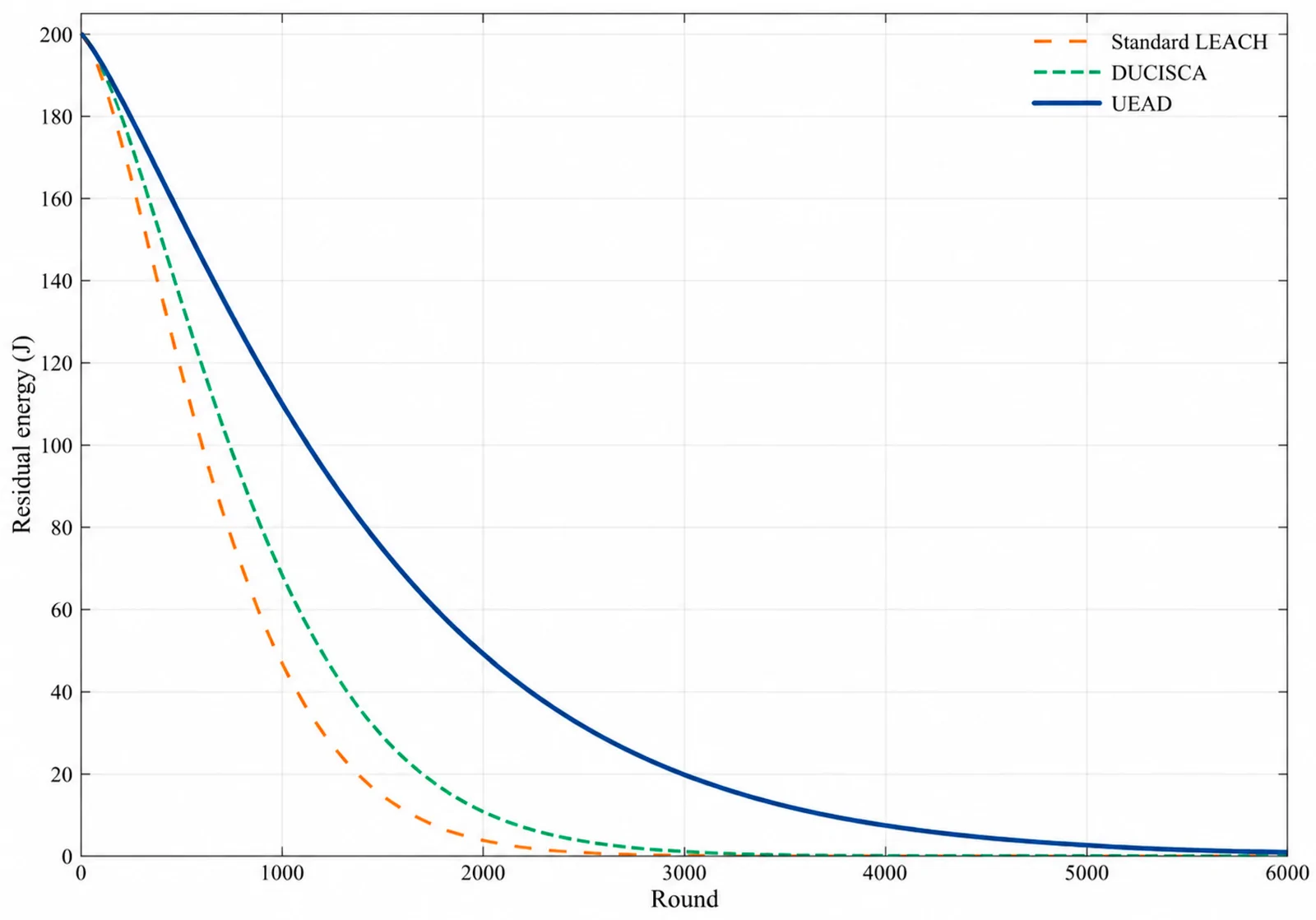
Residual energy under the three protocols.

A longer network lifetime does not necessarily indicate lower overall energy consumption, as it may result from energy remaining in a small number of lightly loaded nodes after earlier failures elsewhere. Figure 22 therefore compares total residual energy over successive rounds to determine whether the lifetime results are accompanied by broader energy conservation across the network.

All three protocols begin with a total network energy of 200 J. The remaining energy decreases as nodes perform intracluster and intercluster data transmission and exchange control messages. Standard LEACH depletes energy most rapidly. Its probabilistic cluster formation produces uneven communication loads, while direct transmission imposes high costs on distant heads when the base station is located at a corner.

DUCISCA retains more energy than standard LEACH over much of the simulation, consistent with its later node deaths. Its residual-energy curve nevertheless remains below that of UEAD and approaches low energy earlier during the middle stage of operation.

UEAD maintains the highest total residual energy at comparable rounds and exhibits a more gradual decline than either baseline. This protocol-level result is consistent with the route-level findings in Section 5.4: shorter routes, fewer relay stages, and fewer reception and retransmission operations reduce communication costs over repeated collection rounds.

Health-driven maintenance also limits the scope of structural updates. Local rotation preserves cluster memberships and replans only the affected routes, whereas global reclustering is performed only when the unhealthy-cluster proportion reaches the specified threshold. This distinction avoids applying network-wide reconstruction to every locally degraded cluster while still allowing broader reorganization when degradation becomes widespread.

Considered together, the survival and residual-energy curves show that UEAD’s lifetime advantage is accompanied by higher energy reserves across the network. The improvement therefore extends beyond the late survival of a small number of lightly loaded nodes and includes longer all-node and midlife service periods.

#### 5.6.4. Overall Performance Discussion

Figure 20, Figure 21 and Figure 22 show progressively better performance from Standard LEACH to DUCISCA to UEAD. Standard LEACH is simple but poorly accommodates distance disparities in large regions with corner base stations. DUCISCA mitigates some energy problems through clustering or routing improvements, but deaths remain concentrated later. UEAD jointly regulates clusters, routes, and lifetime maintenance.

The improvement comes from hierarchical coordination throughout the lifetime rather than selecting the highest-energy node at one instant. Unequal clustering adjusts member loads and distant source-flow counts during formation; EPPR–ACO reduces transmission costs; and dual thresholds update topology from actual degradation. Overall performance reflects these stage-specific mechanisms acting together.

### 5.7. Base-Station Location Sensitivity and Spatial Adaptability

Base-station placement affects node-to-base-station distances, traffic direction, and the spatial distribution of relay loads. Evaluation at a single location cannot show how strongly UEAD depends on the geometry of the collection scenario. We therefore examine three base-station locations:

(36) $${\mathrm{BS}}_{1}=\left(0,\;0\right)$$

(37) $${\mathrm{BS}}_{2}=\left(100,\;100\right)$$

(38) $${\mathrm{BS}}_{3}=\left(100,\;250\right)$$

The base station is placed at (0, 0) in the corner scenario, at (100, 100) in the central scenario, and at (100, 250) in the external scenario. No complete route set can be constructed for the external location using the original communication radius of 65 m. To maintain a common reachability condition, the communication radius is therefore increased by 20%, from 65 to 78 m, in all three scenarios. The node deployment, initial energy, radio model, clustering parameters, and other protocol settings remain unchanged. The three scenarios represent corner-based directional collection, central collection, and external directional collection, respectively, and are used to examine the effects of base-station placement on network lifetime and energy balance. Any feasibility conclusions from this experiment are limited to these three locations under the common 78 m communication radius.

#### 5.7.1. Surviving-Node Evolution by Location

**Figure 23:**
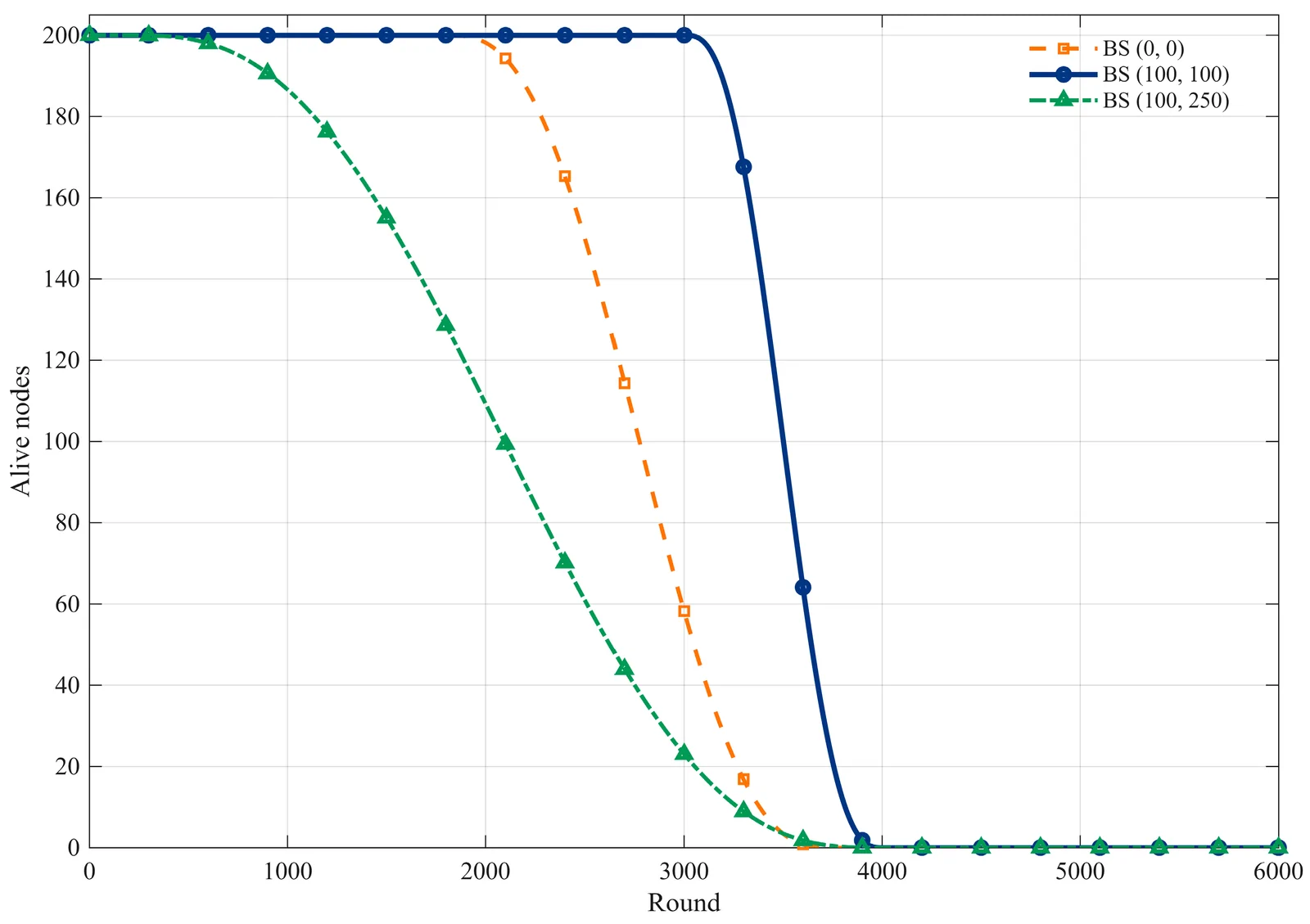
Surviving nodes at different base-station locations.

Figure 23 compares the surviving-node trajectories obtained for the corner, central, and external base-station locations under the common 78 m communication radius.

The central base station at (100, 100) produces the most favorable overall survival trajectory. Its location reduces both the average collection distance and the spatial disparity in source-to-base-station distances. Cluster heads consequently use shorter direct or multihop routes, while forwarding traffic approaches the base station from multiple directions rather than converging on a single boundary region. This configuration retains a larger surviving-node population for longer.

With the base station at the corner position (0, 0), collection traffic is strongly directional. Heads in the upper-right region require longer multihop routes to reach the lower-left base station, and ordinary nodes near the collection destination are eligible for a larger number of forwarding paths. Distance-adaptive clustering regulates nearby cluster coverage, while exclusive relay assignment prevents individual ordinary nodes from serving multiple active routes simultaneously. These mechanisms limit, but do not eliminate, the load concentration imposed by the corner geometry.

At the external position (100, 250), the average collection distance increases and traffic converges toward the upper boundary of the monitoring area. Ordinary nodes nearer that boundary are more likely to participate in forwarding, whereas sources in the lower part of the area require longer relay chains. This directional concentration is accompanied by an earlier onset of node death and a more pronounced decline during the early stage.

#### 5.7.2. Lifetime Metrics by Base-Station Location

**Figure 24:**
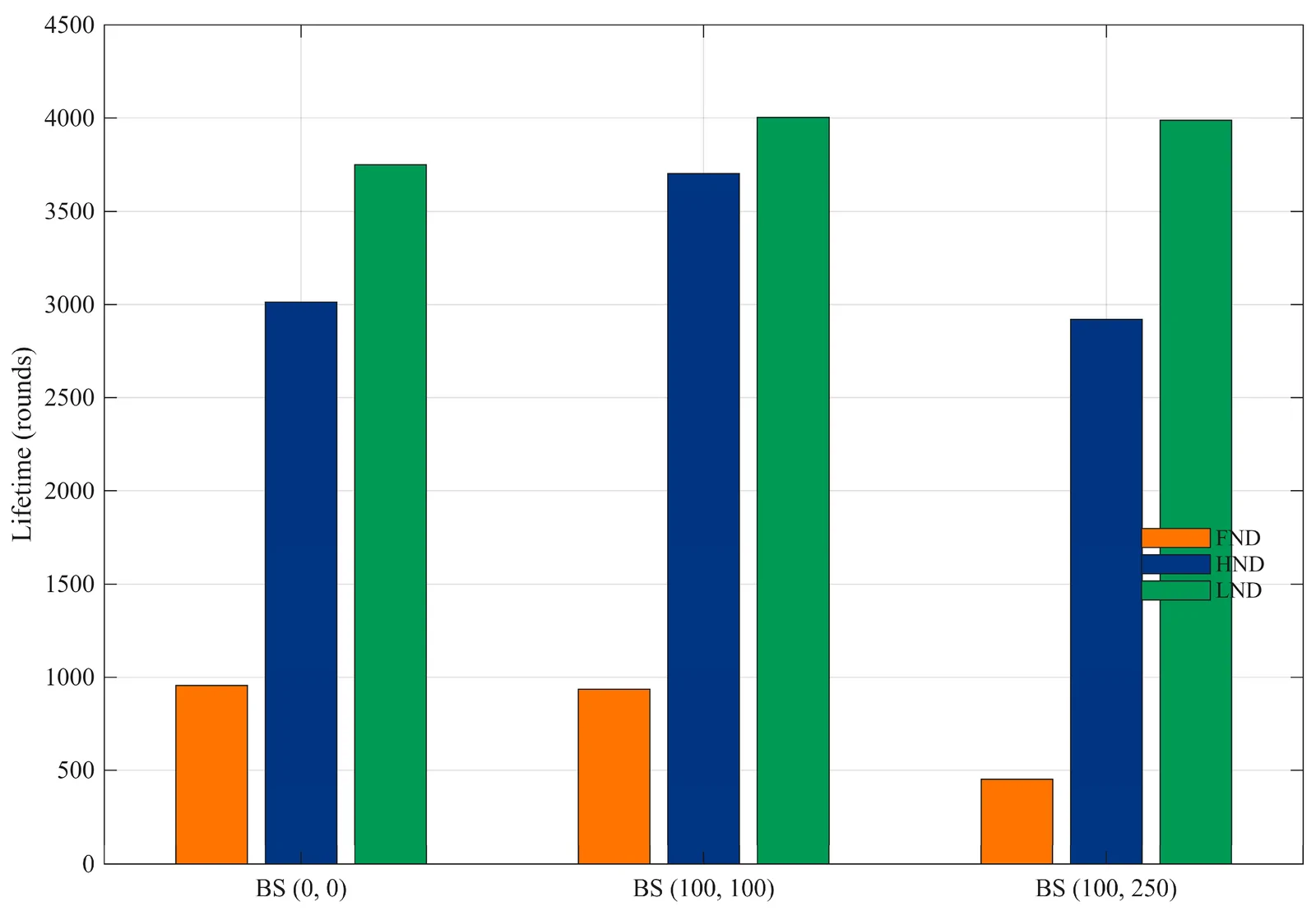
Lifetime metrics at different base-station locations.

Figure 24 compares FND, HND, and LND across the corner, central, and external base-station locations under the common 78 m communication radius.

The corner and central base-station locations provide relatively long all-node survival periods, whereas external placement produces a markedly lower FND. Moving the base station outside the monitoring area increases the collection distances and concentrates traffic toward the upper boundary. Nodes carrying the resulting long-distance transmission or forwarding loads are therefore more likely to deplete early.

The central location provides the strongest HND performance. Shorter average collection distances and less directional convergence distribute communication demands more evenly across the network, allowing a larger proportion of nodes to survive into the middle stage. In contrast, the corner and external locations direct traffic toward particular boundary regions, increasing the communication burden on nodes along those collection paths.

The central location also provides the longest, or nearly the longest, LND. Although the external scenario has a relatively long tail, its substantially lower FND shows that this late survival follows an earlier onset of node loss. The combination of early failures and a persistent tail indicates greater variation in cumulative energy consumption across nodes.

Base-station placement therefore affects the three lifetime stages differently. FND is determined by the earliest depletion of a heavily loaded node, HND reflects the broader distribution of communication costs across the network, and LND is influenced by the small number of nodes that retain energy during the final stage.

#### 5.7.3. Residual Energy by Base-Station Location

**Figure 25:**
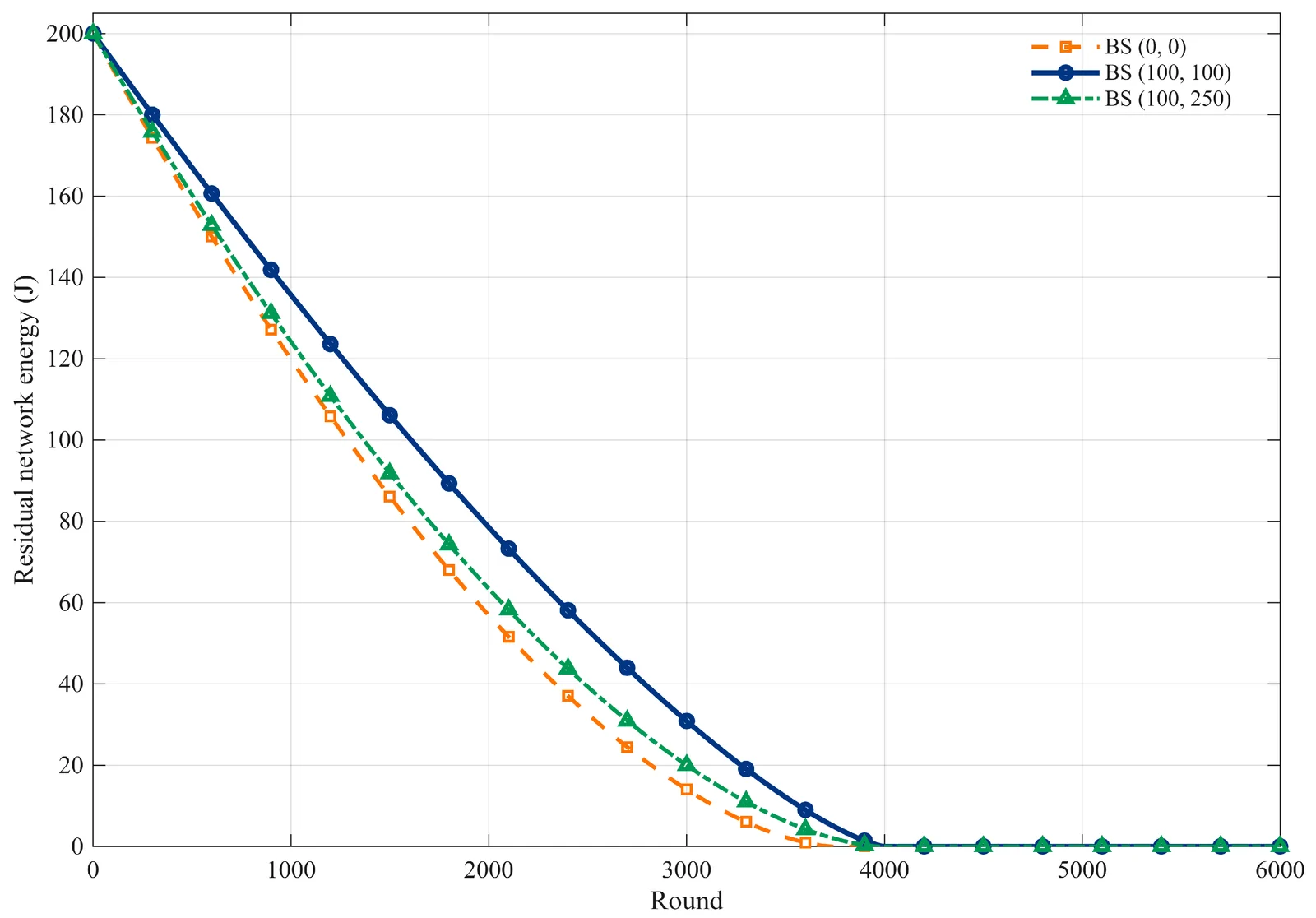
Residual energy at different base-station locations.

Figure 25 compares the evolution of total residual energy for the three base-station locations under the common 78 m communication radius.

At comparable rounds, the central base-station location generally retains the highest total residual energy. Its shorter average collection distances permit shorter routes and fewer relay operations, reducing the cumulative communication cost. The residual-energy curve indicates that this advantage remains after the control costs of cluster-head rotation and global reclustering are included.

Total residual energy declines more rapidly with the base station at the corner. Distant cluster heads require multihop routes through ordinary nodes to reach the collection point. Although EPPR–ACO limits unnecessary detours, it cannot remove the longer source-to-base-station distances imposed by the corner geometry. Repeated transmission, reception, and retransmission therefore produce higher cumulative data-communication costs than in the central scenario.

The residual energy for the external base station generally lies between the central and corner cases. However, this intermediate total does not imply an equally balanced distribution among nodes. Traffic converges toward the external collection direction, causing some nodes along the principal forwarding paths to deplete earlier while nodes outside those paths retain more energy.

#### 5.7.4. Energy Balance Among Surviving Nodes

**Figure 26:**
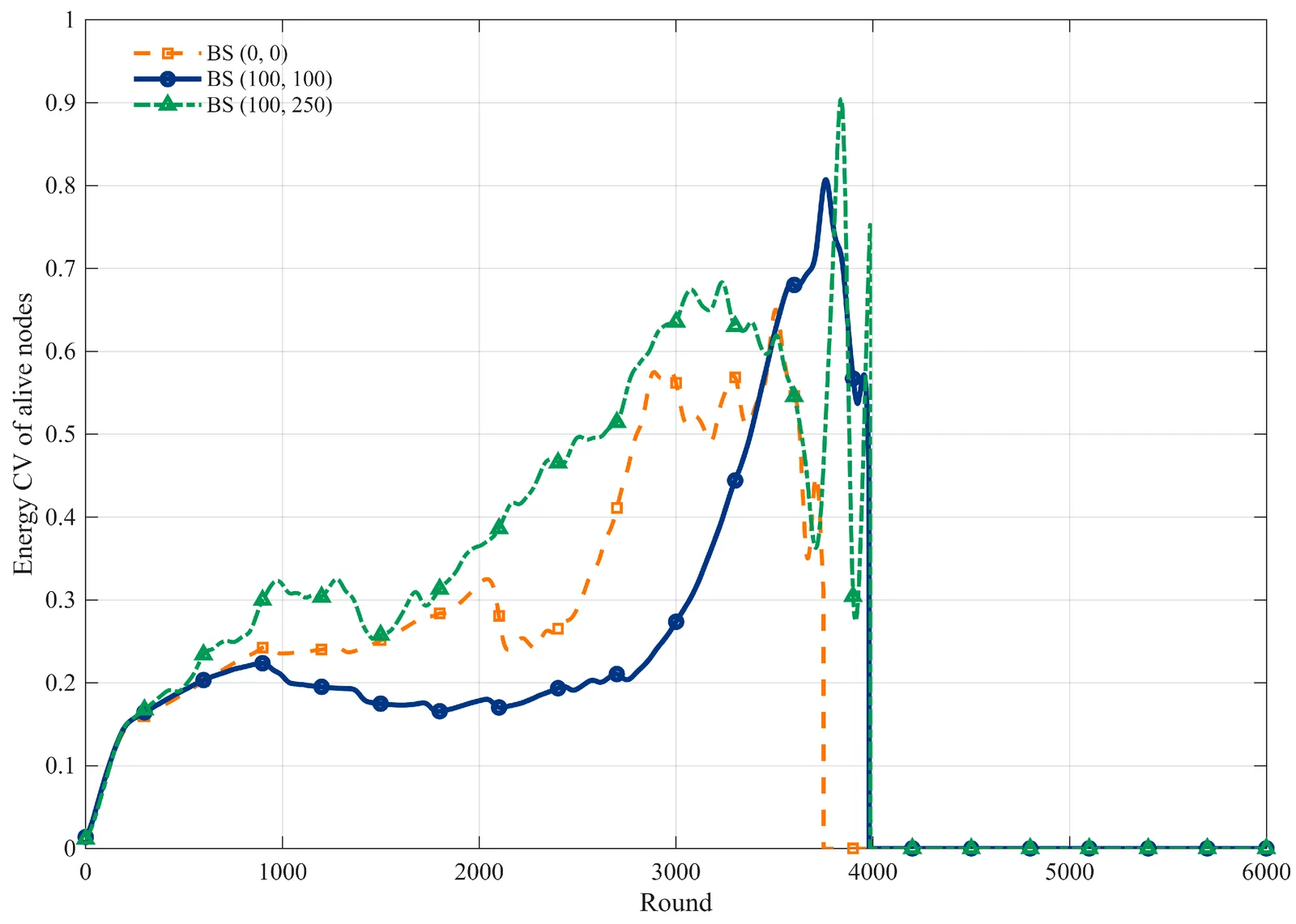
Energy dispersion at different base-station locations.

Figure 26 compares the dispersion of residual energy among surviving nodes over successive rounds for the corner (0, 0), central (100, 100), and external (100, 250) base-station locations. The coefficient of variation is defined as the standard deviation of residual energy among the surviving nodes in a given round divided by their mean residual energy:

(39) $$CV_{E}\left(r\right)=\frac{\sigma_{E}\left(r\right)}{\mu_{E}\left(r\right)},$$

Here, r is the round index, while $\sigma_{E}\left(r\right)$ and $\mu_{E}\left(r\right)$ denote the standard deviation and mean, respectively, of the residual energy among surviving nodes in round r. A lower ${\mathrm{CV}}_{E}\left(r\right)$ indicates less relative dispersion and a more balanced residual-energy distribution, whereas a higher value indicates greater divergence in cumulative energy consumption.

The coefficient of variation initially rises from near zero in all three scenarios. Although the nodes begin with identical energy, they subsequently operate as members, cluster heads, or relays and therefore incur different communication costs. Differences in transmission distance and role frequency accumulate over successive rounds, producing increasing dispersion in residual energy.

The central scenario at (100, 100) maintains the lowest, or nearly the lowest, coefficient of variation over an extended period. Shorter collection paths and multidirectional traffic reduce persistent forwarding concentration in any single region. Residual energy consequently remains more evenly distributed among the surviving nodes.

The corner scenario at (0, 0) exhibits greater midlife energy dispersion than the central case. Traffic converges toward the lower-left corner, creating spatial differences in relay-selection frequency and cumulative forwarding cost. Distance-adaptive clustering regulates member loads, and exclusive relay assignment prevents simultaneous sharing across active routes, but these mechanisms do not eliminate the directional effect of base-station placement.

The external scenario at (100, 250) shows greater dispersion during the early and middle stages. Traffic converges toward the upper boundary, placing larger cumulative communication demands on nodes along the principal collection paths. Other nodes participate less frequently in forwarding and retain more energy. This uneven depletion is consistent with the combination of an earlier FND and a relatively persistent late-survival tail.

Near network termination, the coefficient of variation can fluctuate sharply because the number of surviving nodes becomes small and their mean residual energy approaches zero. Under these conditions, the death or energy change in a single node can substantially alter the ratio. Energy-balance comparisons should therefore emphasize the trajectories before widespread node loss rather than isolated peaks near termination.

#### 5.7.5. Location Sensitivity and Spatial Adaptability

UEAD performance varies with base-station placement. On the fixed node deployment and with the common 78 m communication radius used in this experiment, the protocol maintains feasible clustering, complete route construction, and health-driven maintenance at the corner, central, and external locations. This result demonstrates operation under the three tested geometries but does not establish robustness across arbitrary node deployments, densities, base-station positions, or communication ranges.

Central placement shortens average collection distances and reduces directional traffic convergence, producing the most favorable HND, LND, total residual energy, and energy-balance results. Corner placement lengthens routes from distant regions and concentrates collection traffic toward the lower-left boundary, increasing the potential forwarding burden near the base station. External placement imposes stronger distance and directional constraints, resulting in earlier node deaths and greater energy dispersion despite a persistent late-survival tail.

The comparison shows that UEAD can reconstruct clusters and routes for different collection geometries when reachability is maintained. Distance-adaptive clustering recalculates competition radii relative to the base-station position, EPPR–ACO replans the collection paths, and the maintenance thresholds update the resulting structure as node energy changes. The observed lifetime and energy-balance differences nevertheless confirm that these mechanisms reduce, but do not eliminate, the effects of base-station placement.

### 5.8. Larger-Area Route-Set Feasibility with Relay-Sharing Fallback

**Figure 27:**
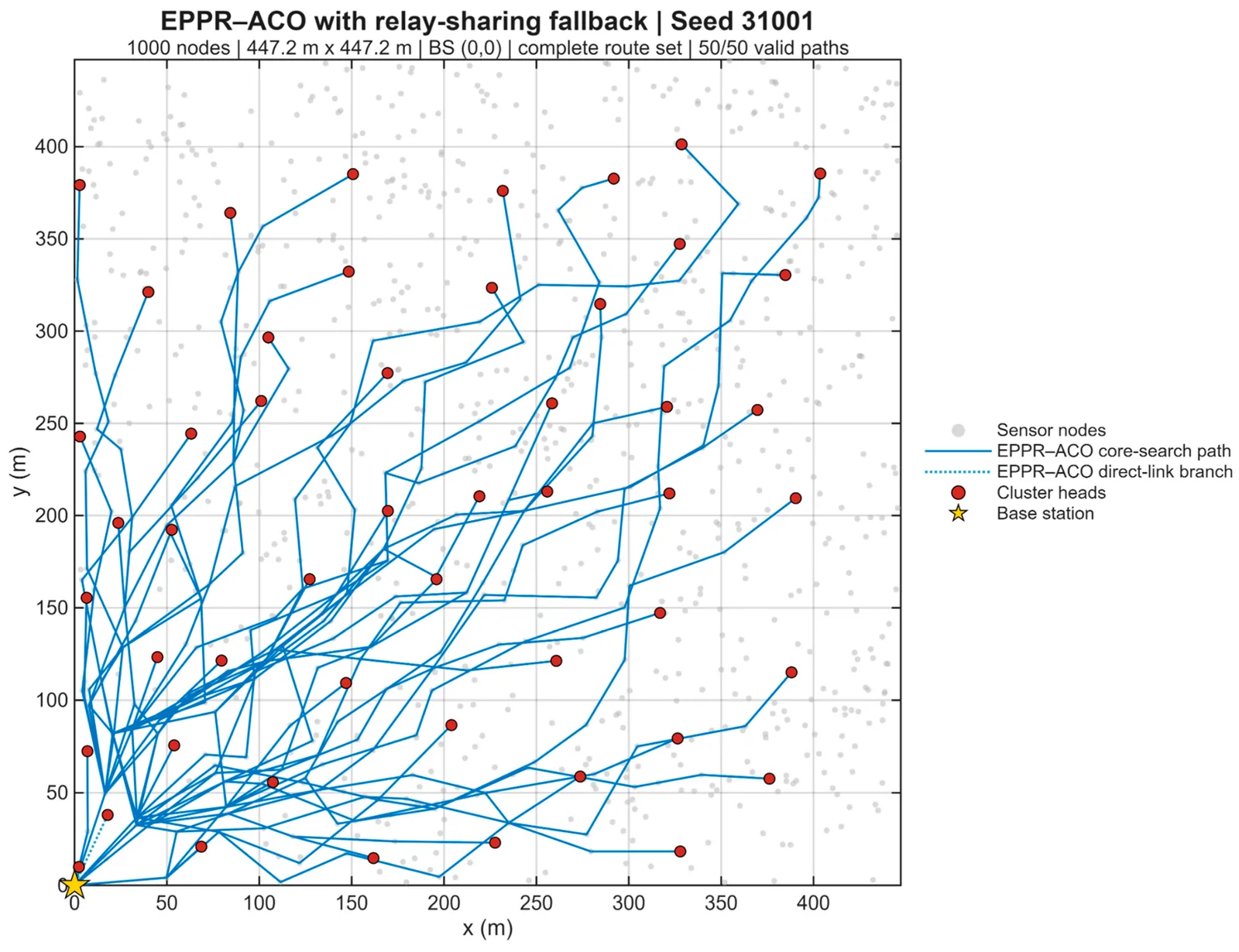
EPPR–ACO routing with relay-sharing fallback.

The larger-area test used 1000 nodes in a 447.2 m × 447.2 m region with the base station at (0, 0), across topology seeds 31001–31005. Under strict relay exclusivity, graph-level joint-feasibility checks supported only 9–15 independent routes, compared with 50–52 source cluster heads. Removing the 12-hop cap alone did not produce a complete exclusive route set. The test therefore retained EPPR–ACO as the primary route constructor and, when strict search returned no feasible path, invoked the BFS fallback described in Section 4.3.4. The fallback used the same communication-range-feasible graph and continued to exclude cluster heads as relays and to require loop-free paths; only relay exclusivity was relaxed. No fixed hop-count cap was applied in this expanded-area test. Figure 27 illustrates the complete fallback-assisted route set for seed 31001. Table 7 reports the complete route-set outcomes for all five topology seeds.

The fallback produced complete route sets in all five tested topologies (50/50–52/52 valid routes). Mean route hop counts ranged from 7.24 to 7.59, maximum hop counts ranged from 14 to 16, mean path communication energy ranged from 3.29 to 3.43 mJ, and planning time ranged from 702 to 817 s per topology. Every reported path satisfied the communication-range, node-role, and loop-free constraints. Relay exclusivity was relaxed only for paths produced by the fallback.

**Figure 28:**
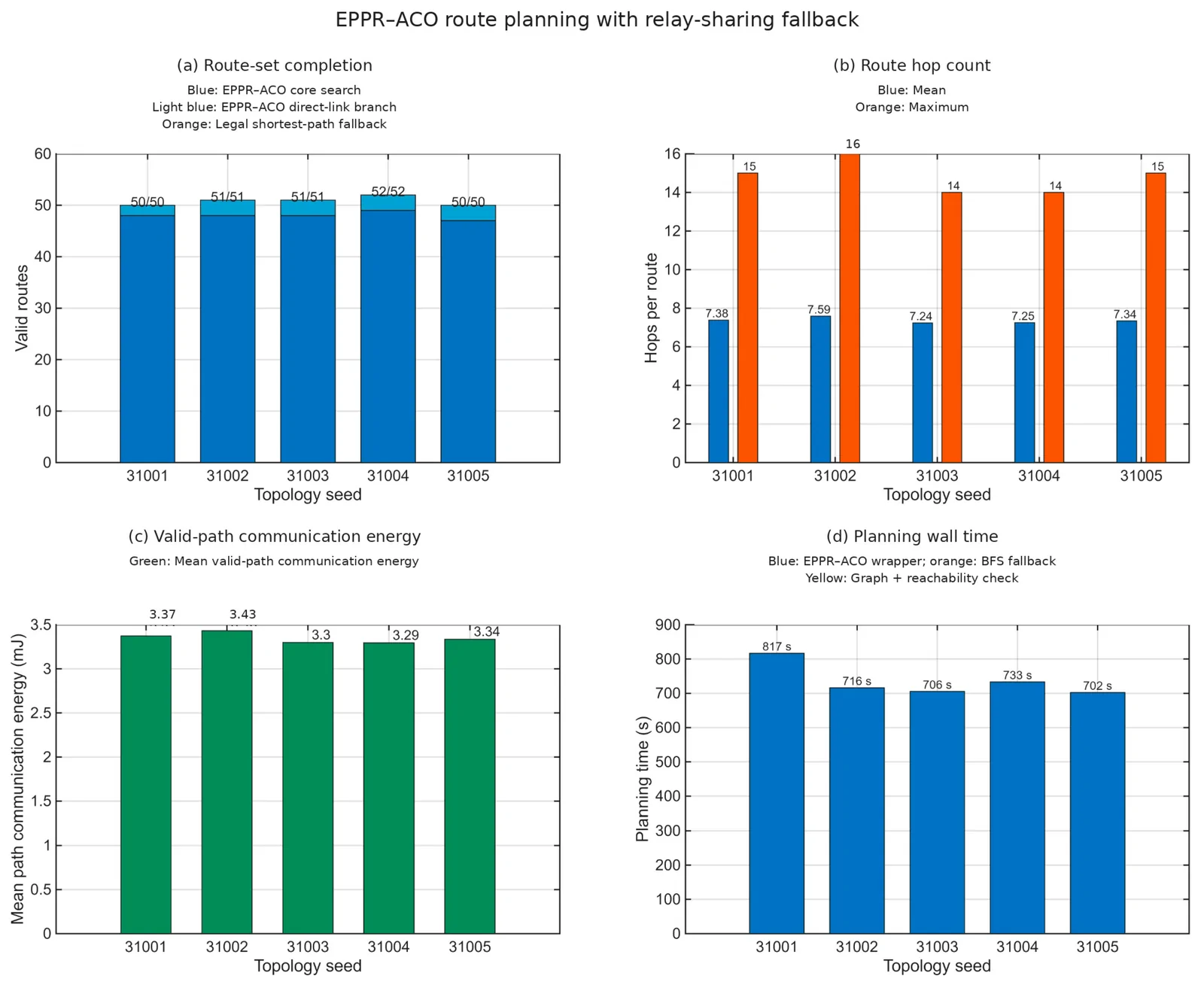
Expanded-area routing results across five topologies.

Planning time covers construction of the complete fallback-assisted route set, not a 50-round or full-lifetime simulation. Figure 28 summarizes the five-topology results.

### 5.9. Network-Size Effects on Control Energy and Runtime

The additional scale test used fixed 200 m × 200 m deployments with 200, 500, and 1000 nodes and measured 50-round operation. For this test, the UEAD maximum-hop cap was disabled while the other route-feasibility rules were retained. Means were calculated from complete paired runs for n = 4, 5, and 3 deployments at the three network sizes, respectively. One 200-node UEAD deployment failed during route initialization and is retained as a failure rather than treated as zero or replaced.

Mean 50-round control energy increased with network size for all three protocols. At 200, 500, and 1000 nodes, the respective values were 3.683, 10.845, and 21.412 mJ for UEAD; 316.060, 981.632, and 2906.137 mJ for Standard LEACH; and 151.431, 533.991, and 1392.632 mJ for DUCISCA. Under the event-accounting models stated above, UEAD had the lowest modeled control energy at each tested size.

The recorded mean initialization times at 200, 500, and 1000 nodes were 2.034, 2.819, and 6.586 s for UEAD; 0.0317, 0.0145, and 0.0264 s for Standard LEACH; and 0.700, 0.869, and 1.143 s for DUCISCA. The corresponding total simulation times through round 50 were 2.707, 6.115, and 283.544 s for UEAD; 0.731, 1.576, and 5.638 s for Standard LEACH; and 1.974, 2.820, and 3.780 s for DUCISCA. Thus, UEAD maintained lower modeled control energy but incurred a substantially longer recorded runtime at 1000 nodes. These timings reflect the current MATLAB implementations and their recorded timer boundaries.

**Figure 29:**
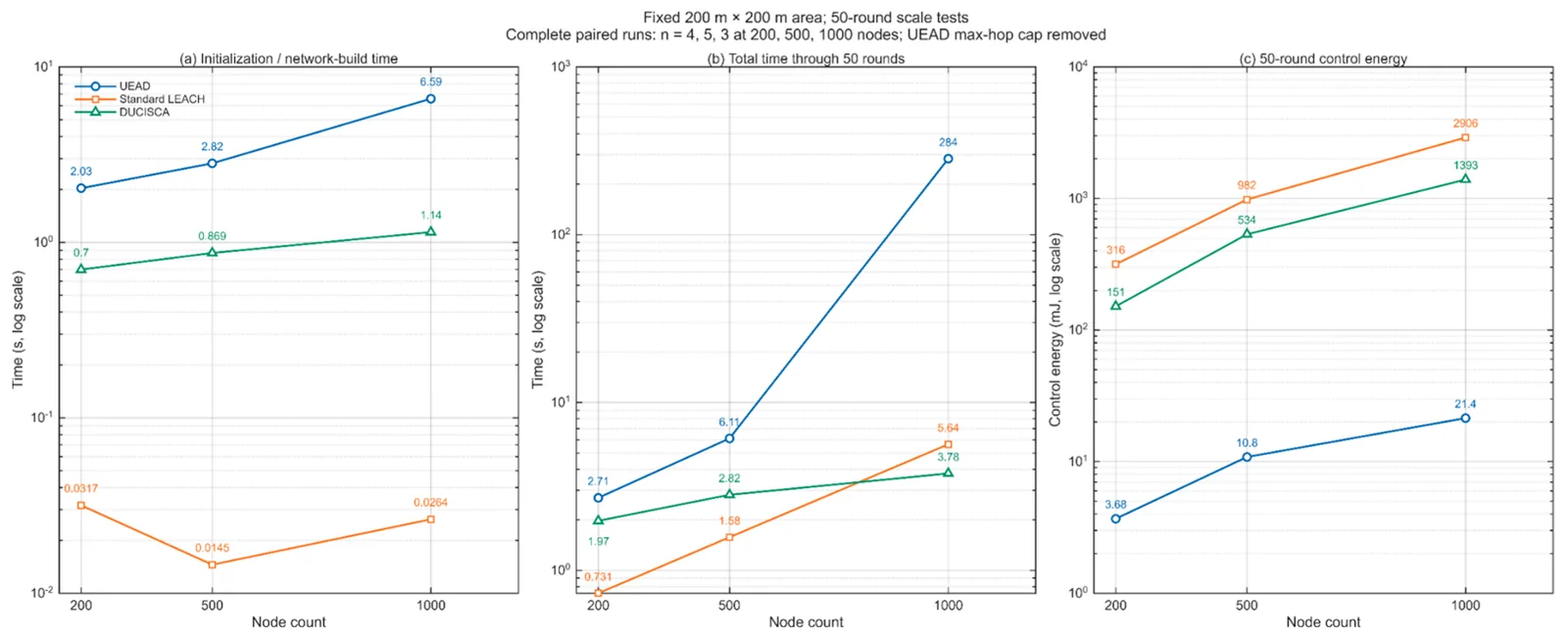
Network-size effects on runtime and control energy.

The total time through round 50 is an end-to-end simulation runtime that includes the operations executed during those rounds; it is not a separately measured duration for global reconstruction alone. Figure 29 summarizes the measured scale effects.

### 5.10. UEAD Aggregation-Level Sensitivity

**Figure 30:**
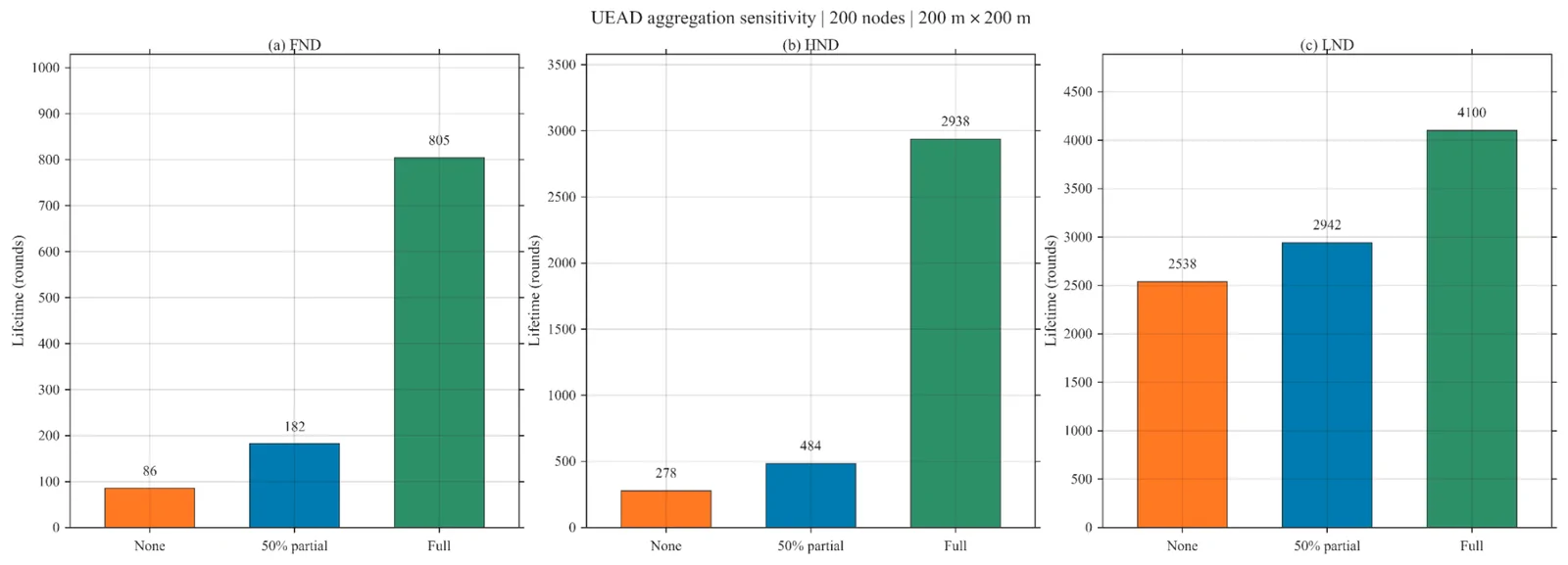
Lifetime sensitivity to data aggregation.

UEAD was evaluated under no aggregation, 50% partial aggregation, and full aggregation using the same five frozen 200-node topologies and saved random states. All other experiment settings were kept the same. Seed 31002 failed during initial route construction at CH 73 under all three aggregation settings and is retained as a failed attempt. Table 8 and Figure 30 report rounded mean lifetime values from the four completed runs per condition (n = 4/5).

Table 8 and Figure 30 show that the rounded mean FND, HND, and LND increased with aggregation level in the four successful deployments: 86/278/2538 rounds with no aggregation, 182/484/2942 with 50% partial aggregation, and 805/2938/4100 with full aggregation. These results indicate a positive association between aggregation level and lifetime in the tested UEAD runs, but the small number of completed topologies and the common initialization failure limit the inference. This experiment assesses aggregation sensitivity within UEAD; it does not compare UEAD with UEAD-Uniform at each aggregation level. The three-protocol comparison uses the common full-aggregation assumption described in Section 5.1.4. The 50% partial-aggregation setting is a fixed payload-reduction ratio and does not explicitly model spatial correlation or heterogeneous sensor readings.

### 5.11. Summary of Results

The results show how routing, clustering, and maintenance affect different aspects of UEAD performance under the evaluated conditions.

Routing-parameter selection prioritizes complete-route construction and then communication energy, giving:

(40) α=0.6,  β=2.0,  ρ=0.6.

The maintenance-threshold comparison balances early survival, midlife survival, tail lifetime, and control overhead. The selected thresholds are:

(41) $$T_{H}=0.8,\;T_{P}=0.5.$$

This combination yields FND, HND, and LND values of approximately 940, 2920, and 4000 rounds, respectively, with 0.2475 J of control energy and 59 global reclustering events. It favors the all-node survival period while retaining strong midlife performance and comparatively low maintenance overhead within the tested range, rather than maximizing LND alone.

The clustering and routing visualizations illustrate the resulting spatial organization and ordinary-node forwarding paths.

EPPR–ACO produces shorter, less circuitous routes in the five-iteration comparison. The extended-budget experiments show lower mean energy for EPPR–ACO at five iterations and at the two shorter time budgets, while ordinary ACO is slightly lower at the longer budgets. These findings concern the complete search implementations; the equal-time evaluation includes EPPR preprocessing.

The ablations show stage-dependent effects. Relative to UC-ACO-D, UEAD improves FND, HND, and LND by 5.89%, 18.19%, and 9.40%, respectively. Compared with fixed-period UC-EA-F, UEAD has lower FND but higher HND and LND. Relative to UEAD-Uniform, unequal clustering increases FND from 841 to 899 rounds, or 6.90%, while HND and LND are 3.57% and 1.29% lower. Unequal clustering therefore improves early survival in this comparison rather than every lifetime metric.

In the overall protocol comparison, UEAD delays FND, HND, and LND relative to standard LEACH and DUCISCA and retains more total residual energy at comparable rounds. Its lifetime advantage thus extends across the onset, midpoint, and completion of node loss rather than relying only on a small number of late-surviving nodes.

Base-station placement changes lifetime and energy balance. Under the common 78 m communication-radius setting, central placement provides the most favorable overall results among the three tested locations, while corner and external placements produce stronger directional traffic concentration.

In the expanded-area experiment, relay-sharing BFS fallback produced complete route sets in all five tested topologies. This establishes route feasibility under the stated fallback; its effects on cumulative relay load and lifetime remain to be evaluated.

Stronger aggregation increases mean FND, HND, and LND across the four completed deployments. The three-protocol lifetime comparison retains the common full-aggregation assumption.

## 6. Limitations and Future Work

This study has several limitations. The results are obtained from MATLAB simulations using a first-order radio energy model and deterministic link-feasibility rules. Although the same topology, energy accounting, and node-death definitions are applied throughout the comparisons, the simulation does not represent channel fading, interference, packet collisions, synchronization errors, or hardware variability. Evaluation in NS-3 and on physical sensor nodes would provide further evidence on communication reliability and measured energy consumption. Full aggregation is an idealized assumption in which each cluster’s readings are represented by one 4000-bit output packet. The sensitivity test is limited to four successful topologies per condition and does not include a separate aggregation-computation cost or establish the UEAD versus UEAD-Uniform ranking under partial aggregation.

UEAD assumes stationary nodes, bidirectional links, complete position and residual-energy information, and centralized planning at the base station. These assumptions are appropriate for the fixed monitoring scenario considered here but limit direct use in mobile, asymmetric-link, or partitioned networks. Node movement can make cluster memberships and routes outdated between updates; maintaining current topology would require more frequent position reporting and route reconstruction, increasing control overhead and planning delay. With asymmetric or lossy links, a path feasible in one direction may not support data or control traffic in the reverse direction, so each direction would need separate link-quality estimation and validation. During a network partition, nodes without a path to the base station cannot report their state or receive updated schedules; centralized planning cannot restore a physically disconnected path. Future work should evaluate mobility-aware route updates, directional link assessment, and local or hierarchical operation during disconnection, followed by route recovery when connectivity returns.

The parameters and thresholds were selected offline and may require adjustment when density, energy, traffic demand, or base-station placement changes. The fixed-area scale test also exposes a computational tradeoff: UEAD has lower modeled control energy but substantially higher runtime at 1000 nodes. Timings depend on the MATLAB implementation and recorded measurement boundaries, while DUCISCA control energy is reconstructed from the adapted implementation’s events. The expanded-area test establishes route feasibility with relay-sharing fallback rather than full-lifetime performance under strict exclusivity.

Because the simulation does not specify a deployment-level data-collection interval or computation deadline, these MATLAB timings cannot determine whether UEAD meets a particular real-time cycle. The expanded-area timing aggregates route-set construction; separate wall-clock durations for individual source-head searches and maintenance-triggered global reconstruction were not recorded.

The equal-time route comparison uses repeated tasks on one topology, and the scale and aggregation tests use a limited number of completed deployments. Future work should extend evaluation across independent topologies and hardware, profile memory and component-level latency, and investigate lower-cost or hierarchical route planning.

## 7. Conclusions

This study presented UEAD, a protocol that combines distance-adaptive unequal clustering, EPPR-initialized ACO multihop routing, and health-driven local and global maintenance. In the tested route tasks, the complete EPPR–ACO implementation had lower mean energy at five iterations, whereas ACO was slightly lower at 20–100 iterations. When total planning time included EPPR preprocessing, EPPR–ACO had lower mean energy at the two shorter tested budgets, while the methods were close and ACO was slightly lower at the longer budgets. These findings describe the tested topology and budgets and do not establish a general advantage of EPPR initialization alone. The ablations revealed distinct contributions from routing, clustering, and maintenance across different lifetime stages. Compared with standard LEACH and DUCISCA, UEAD delayed node loss and retained more residual energy under the tested conditions. These results support coordinated clustering, routing, and maintenance as a practical direction for energy-efficient data collection in static WSNs. In the UEAD-only aggregation-sensitivity test, rounded mean FND, HND, and LND increased with aggregation level across the four successful tested topologies.

## Figures and Tables

**Table 1:** Simulation parameters.

Parameter	Symbol	Value
Monitoring area	*M × M*	200 m × 200 m
Number of sensor nodes	*N*	200
Default base-station position	*BS*	(0, 0)
Initial node energy	*E*_0_	1 J
Electronics energy	*E*_elec_	50 nJ/bit
Free-space amplifier coefficient	*ε_fs_*	10 pJ/(bit·m^2^)
Multipath amplifier coefficient	*ε_mp_*	1.3 × 10^−15^ J/(bit·m^4^)
Crossover distance	*d*_0_	87.706 m
Data packet length	$l_{\mathrm{data}}$	4000 bit
Control packet length	$l_{\mathrm{ctrl}}$	100 bit
Minimum competition radius	$R_{\min}$	55 m
Maximum competition radius	$R_{\max}$	65 m
Pheromone weight	α	0.6
Heuristic weight	β	2.0
Evaporation coefficient	ρ	0.6
EPPR damping factor	δ	0.8
EPPR L1 convergence tolerance	$\varepsilon_{EPPR}$	1 × 10^−9^
Maximum EPPR iterations	$K_{EPPRmax}$	200
Initial pheromone scale	τ_0_	1.0
Ants per ACO search (default)	$n_{ants}$	20
ACO iterations per search (default)	$N_{iter}$	50
Maximum ant route constructions per source CH	$B_{max}$	4000 (4 × 20 × 50; upper bound)
Search attempts per source CH	Nmax	4 (1 initial + up to 3 restarts)
Cluster-health threshold	$T_{H}$	0.8
Unhealthy-cluster proportion threshold	$T_{P}$	0.5
Health-check interval	*T*_check_	50 rounds
UC-EA-F fixed reclustering interval	$T_{F}$	50 rounds
Competition-radius exponent	γ	0.8

**Table 2:** Path structures and communication energy of ACO and EPPR–ACO.

Metric	ACO	EPPR–ACO	Change
Crossing count	38	20	Reduction of 47.37%
Mean path stretch	1.420	1.074	Reduction of 24.37%
Mean hop count	5.17	3.75	Reduction of 27.47%
Total path length	2957.4 m	2191.4 m	Reduction of 25.90%
Total communication energy	30.321 mJ	22.000 mJ	Reduction of 27.44%

**Table 3:** Success counts and mean route communication energy under equal total planning-time budgets.

Budget (ms)	Success ACO/EPPR–ACO	Joint *n*	Energy ACO/EPPR–ACO (mJ)
62	328/330/328/330	326	2.4912/1.9341
149	330/330/329/330	329	2.0341/1.9305
300	330/330/330/330	330	1.8991/1.9312
541	330/330/330/330	330	1.8953/1.9312

**Table 4:** Route-quality metrics across ACO iteration budgets.

ACO Iterations	Mean Route Energy (mJ) ACO/EPPR–ACO	Mean Path Length (m) ACO/EPPR–ACO	Mean Hops ACO/EPPR–ACO	Successful Paths ACO/EPPR–ACO
5	2.348 ± 0.869/1.940 ± 0.580	231.165 ± 84.336/189.758 ± 55.006	5.276 ± 1.757/4.497 ± 1.196	330/330/330/330
20	1.896 ± 0.570/1.931 ± 0.568	198.707 ± 60.189/189.158 ± 54.279	4.267 ± 1.109/4.476 ± 1.167	330/330/330/330
50	1.895 ± 0.568/1.931 ± 0.568	198.690 ± 60.168/189.158 ± 54.279	4.264 ± 1.103/4.476 ± 1.167	330/330/330/330
100	1.895 ± 0.568/1.931 ± 0.568	198.690 ± 60.168/189.158 ± 54.279	4.264 ± 1.103/4.476 ± 1.167	330/330/330/330

**Table 5:** Module ablation configurations.

Protocol	Unequal Clustering	EPPR Initialization	ACO Multihop	Dynamic Reclustering
UC-ACO-D	√	—	√	√
UC-EA-F	√	√	√	—
UEAD-Uniform	—	√	√	√
UEAD	√	√	√	√

Note: √ indicates that the module is included; — indicates that it is not included.

**Table 6:** Lifetime results of the module ablations.

Protocol	FND (Rounds)	HND (Rounds)	LND (Rounds)
UC-ACO-D	849	2425	3701
UC-EA-F	1214	2405	3574
UEAD-Uniform	841	2972	4102
UEAD	899	2866	4049

**Table 7:** Complete route-set outcomes in the expanded-area test using relay-sharing fallback.

Seed	Source CHs/Valid Routes	Mean Hops	Maximum Hops	Mean Path Energy (mJ)	Planning Time (s)
31001	50/50	7.38	15	3.37	817
31002	51/51	7.59	16	3.43	716
31003	51/51	7.24	14	3.30	706
31004	52/52	7.25	14	3.29	733
31005	50/50	7.34	15	3.34	702

**Table 8:** Rounded mean FND, HND, and LND under different aggregation levels (rounds).

Aggregation Level	n (Success/5)	FND (Rounds)	HND (Rounds)	LND (Rounds)
None (γ = 0)	4/5	86	278	2538
Partial 50% (γ = 0.5)	4/5	182	484	2942
Full (γ = 1)	4/5	805	2938	4100

## Data Availability

Data are contained within the article.
